# Quality of Life After Bariatric Surgery—a Systematic Review with Bayesian Network Meta-analysis

**DOI:** 10.1007/s11695-021-05687-1

**Published:** 2021-10-11

**Authors:** Piotr Małczak, Magdalena Mizera, Yung Lee, Magdalena Pisarska-Adamczyk, Michał Wysocki, Małgorzata M. Bała, Jan Witowski, Mateusz Rubinkiewicz, Alicja Dudek, Tomasz Stefura, Grzegorz Torbicz, Piotr Tylec, Natalia Gajewska, Tanawat Vongsurbchart, Michael Su, Piotr Major, Michał Pędziwiatr

**Affiliations:** 1 Department of Medical Education, Jagiellonian University Medical College, Medyczna 7 , 30-688 Cracow, Poland; 2 2nd Department of General Surgery, Jagiellonian University Medical College, Cracow, Poland; 3 Division of General Surgery, McMaster University, Hamilton, ON Canada; 4 Department of General Surgery and Surgical Oncology, Ludwik Rydygier Memorial Hospital in Cracow, Cracow, Poland; 5 Chair of Epidemiology and Preventive Medicine, Department of Hygiene and Dietetics, Jagiellonian University Medical College, Cracow, Poland

**Keywords:** Bariatric surgery, Quality of life, Network meta-analysis

## Abstract

**Objective:**

Comprehensive analysis and comparison of HRQoL following different bariatric interventions through systematic review with network meta-analysis.

**Background:**

Different types of bariatric surgeries have been developed throughout the years. Apart from weight loss and comorbidities remission, improvement of health-related quality of life (HRQoL) is an important outcome of metabolic surgery.

**Methods:**

MEDLINE, EMBASE, and Scopus databases have been searched up to April 2020. Inclusion criteria to the analysis were (1) study with at least 2 arms comparing bariatric surgeries; (2) reporting of HRQoL with a validated tool; (3) follow-up period of 1, 2, 3, or 5 years. Network meta-analysis was conducted using Bayesian statistics. The primary outcome was HRQoL.

**Results:**

Forty-seven studies were included in the analysis involving 26,629 patients and 11 different surgeries such as sleeve gastrectomy (LSG), gastric bypass (LRYGB), one anastomosis gastric bypass (OAGB), and other. At 1 year, there was significant difference in HRQoL in favor of LSG, LRYGB, and OAG compared with lifestyle intervention (SMD: 0.44; 95% CrI 0.2 to 0.68 for LSG, SMD: 0.56; 95% CrI 0.31 to 0.8 for LRYGB; and SMD: 0.43; 95% CrI 0.06 to 0.8 for OAGB). At 5 years, LSG, LRYGB, and OAGB showed better HRQoL compared to control (SMD: 0.92; 95% CrI 0.58 to 1.26, SMD: 1.27; 95% CrI 0.94 to 1.61, and SMD: 1.01; 95% CrI 0.63 to 1.4, respectively).

**Conclusions:**

LSG and LRYGB may lead to better HRQoL across most follow-up time points. Long-term analysis shows that bariatric intervention results in better HRQoL than non-surgical interventions.

**Graphical abstract:**

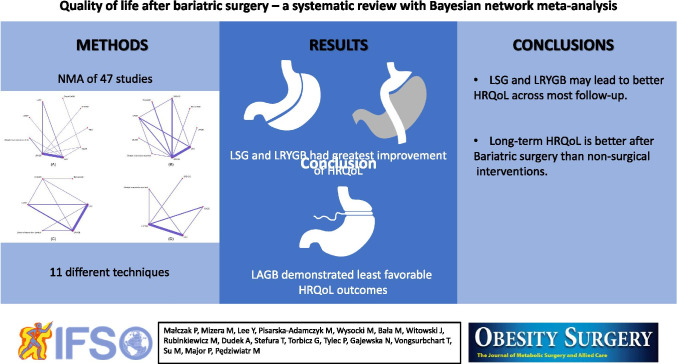

**Supplementary Information:**

The online version contains supplementary material available at 10.1007/s11695-021-05687-1.

## Introduction

Depression, anxiety, and low self-esteem can be significant psychological consequences of obesity [1]. Both physical and psychological consequences can be debilitating and decrease overall Health-Related Quality of Life (HRQoL). Bariatric surgery causes significant changes in patients’ life, including HRQoL. There is a growing body of literature demonstrating an improvement of both the physical and psychological status of the patients following bariatric procedures [2].

Objective assessment of QoL can be made using validated and standardized tools. A variety of those tools have gained popularity among researchers, but HRQoL assessment tools most commonly used in surgical literature are Short Form-36 (SF-36) [3], Moorehead-Ardelt Quality of Life [4], Gastrointestinal Quality of Life (GIQLI) [5], and Impact of Weight on Quality of Life (IWQOL) [6]. Although there are numerous studies on HRQoL after different bariatric procedures, the heterogeneity of different tools used and types of bariatric procedures make the interpretation of these data complex. Considering the paucity of literature on this topic, we aim to conduct a systematic review and a network meta-analysis to produce a comprehensive comparison of HRQoL after different bariatric procedures.

## Methods

### Search Strategy

A search was conducted by five teams, two researchers in each, in April 2020 covering Medical Literature Analysis and Retrieval System Online (MEDLINE), Excerpta Medica dataBASE (EMBASE), and Scopus database. There were no language limitations in the search. A full search strategy for the OVID platform is available in supplement files. This study was reported according to the Preferred Reporting Items for Systematic Reviews (PRISMA) guidelines network meta-analysis extension [7]. The protocol of this study was registered before commencement in the Prospective Register of Systematic Reviews (PROSPERO, CRD42019132975).

### Eligibility Criteria

The analyzed population involved patients with severe obesity who underwent bariatric surgery. Studies were eligible for inclusion if they were randomized controlled trials (RCT) or non-randomized studies with a control group, such as cohort studies (prospective or retrospective). We decided to include non-randomized studies to increase the number of interventions that could be compared. Letters, editorials, case reports, case-series, and review papers were excluded. The included study had to comprise of at least two arms (one of which is bariatric surgery) and the follow-up period was 1 year, 2 years, 3 years, or 5 years. Published abstracts were not included due to limited information available for analysis and the risk of bias assessment. Studies must have reported on health-related HRQoL using any validated tools. The authors of primary studies were contacted in case of missing data.

### Outcome Measures

The primary outcome of this systematic review was health-related quality of life (HRQoL) at 1 year, 2 years, 3 years, and 5 years after bariatric surgery. Secondary outcomes involved specific domains of HRQoL (vitality, physical functioning, bodily pain, general health perceptions, physical role functioning, emotional role functioning, social role functioning, and mental health). Tools which were used for the assessment included The Bariatric Quality of Life (BQL) [8], The Laval Questionnaire [9], GERD-Health-Related Quality of Life Questionnaire (GERD-HRQL) [10], World Health Organization Quality of Life Instruments (WHOQOL-BREF) [11], Short Form 8 Health Survey (SF-8) [12], Bariatric Analysis and Reporting Outcome System (BAROS) [13], The Moorehead-Ardelt Quality of Life Questionnaire (MA) [4], Gastrointestinal Quality of Life Index (GIQLI) [5], The Short Form 36 Health Survey (SF-36) [3], Obesity and Weight-Loss Quality of Life (OWLQOL) [14], Rand 36-Item Health Survey (RAND-36) [3], and The Impact of Weight on Quality of Life-Lite (IWQOL-Lite)[6].

### Study Selection and Data Extraction

Each of the records downloaded from searches was screened by at least two researchers independently. All teams identified and selected citations first on the basis of titles and abstracts and then full texts. In case of disagreement, an attempt was made for reaching a consensus within the group. If no resolution was possible, an arbitrary decision was made by the third reviewer. Data from included studies were extracted independently by two researchers to a prepared Excel sheet. When available, the following data were extracted: first author, year of publication, country, number of operated patients, type of intervention, type of study, HRQoL form, and outcomes of interest (endpoint data). Whenever standard deviation was missing, it was derived as shown by Fu et al. using an average coefficient of variation [15].

### Study Quality

Study quality was assessed by two researchers independently. Observational studies were evaluated using the Newcastle–Ottawa Scale (NOS), which consists of three domains: patient selections, comparability of the study groups, and the assessment of outcomes [16]. Randomized controlled trials were assessed using The Cochrane Collaboration’s Risk of bias tool [17].

### Statistical Analysis

Statistical analysis was performed using WinBUGS 1.4 (BUGS project, MRC Biostatistics Unit, University of Cambridge). Network meta-analysis was conducted using Bayesian statistics according to Markov chain Monte Carlo methods. The model used for calculation was derived from generalized linear models for random effects presented by Dias et al. in *Network Meta-Analysis for Decision Making* and it is shown in Supplement File 2 [18]. To pool data from different HRQoL forms, standardized mean differences (SMD) were used and results on graphs are presented as SMD with 95% credible interval (CrI), while in tables the SMDs are converted to GIQLI scale [19]. Minimal clinically important differences (MCID) for GIQLI were considered at 5 points [20]. The model was run using three chains with an initial burn-in sampling of 10 000 per chain. Initial values for each chain were generated randomly. Statistical heterogeneity between the studies was assessed through the residual deviance of each model. Publication bias was assessed by visually inspecting the asymmetry of the funnel plot for analyses which included at least 10 studies.

## Results

**Fig. 1:**
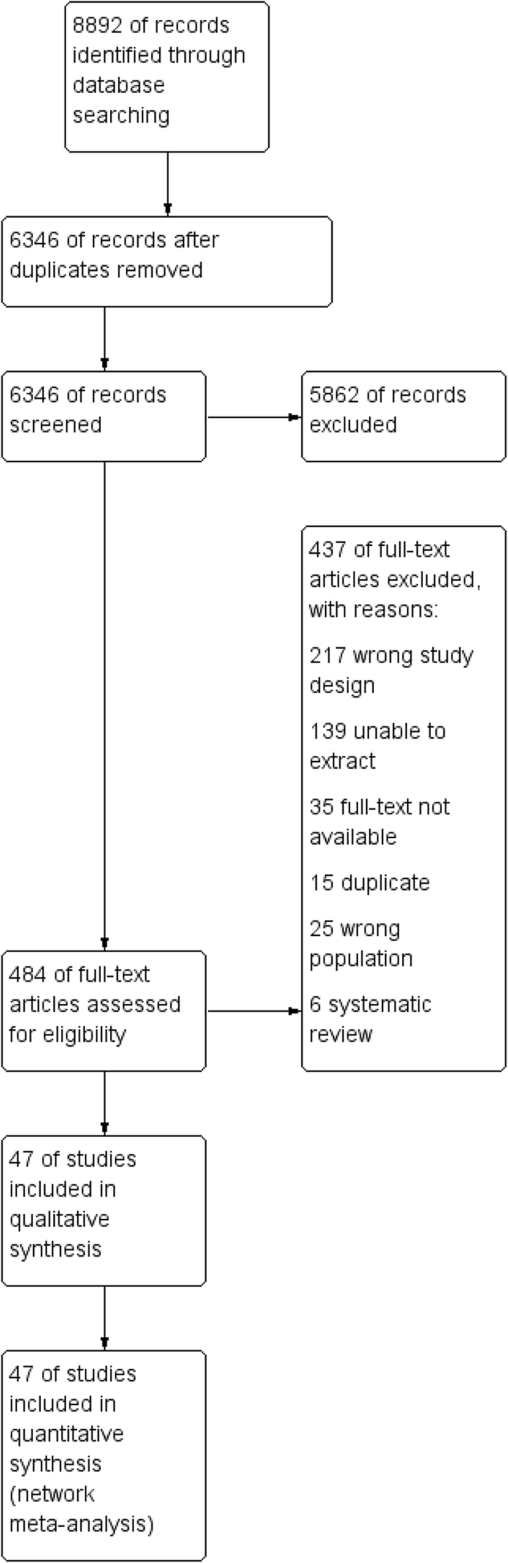
PRISMA flowchart

**Fig. 2:**
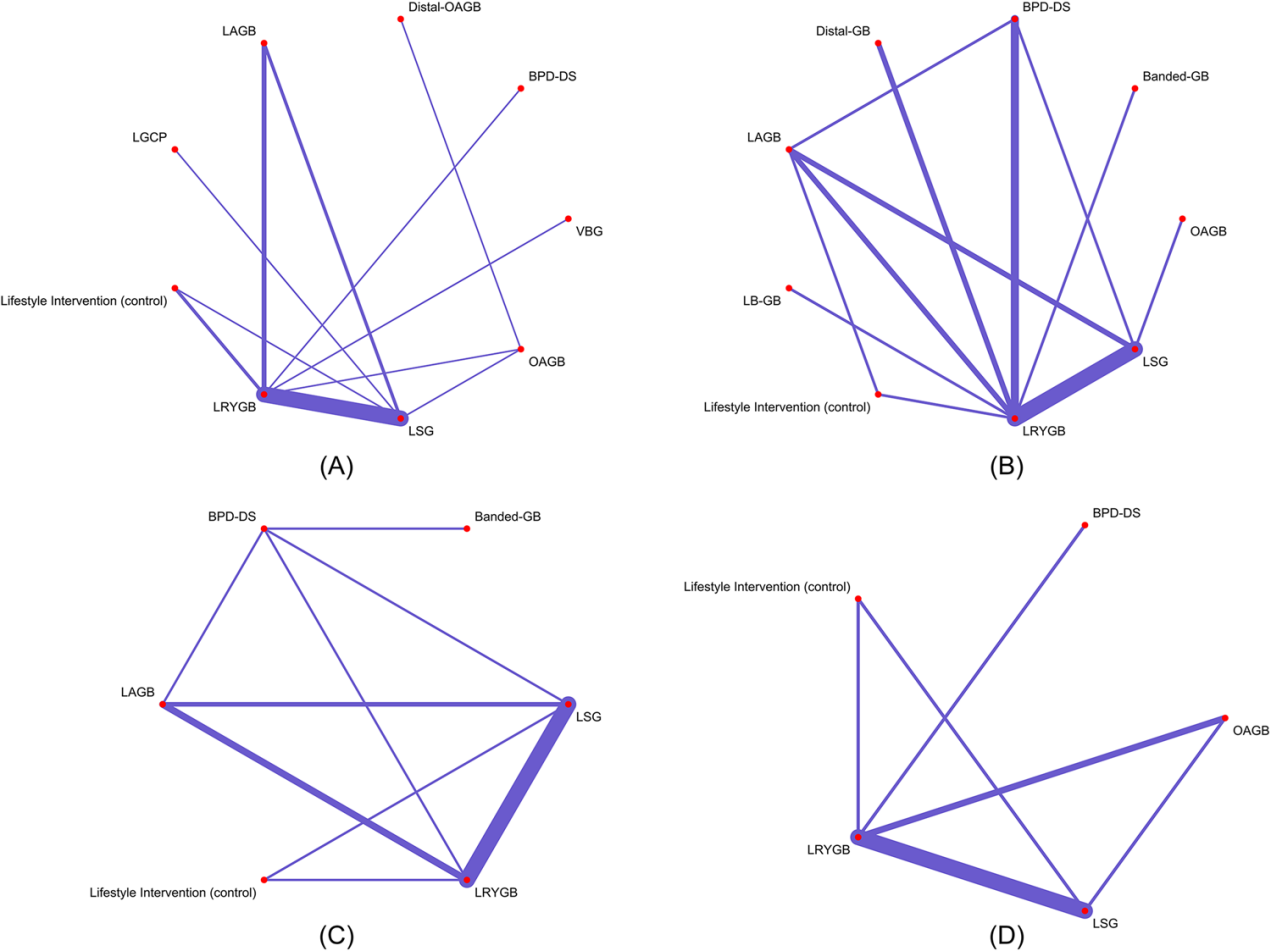
Study network in the meta-analysis **A** at 1 year, **B** at 2 years, **C** at 3 years, **D** at 5 years follow-up

The initial reference search yielded 8892 records. After removing duplicates, 6346 titles and abstracts were reviewed and 484 papers were selected for full-text screening. Finally, 47 studies (17 RCTs and 30 non-RCTs) conducted in 17 countries were included in the network meta-analysis (Fig. 1). The studies included a total of 26,629 patients. A total of 11 surgical procedures were evaluated in the primary studies, which included laparoscopic sleeve gastrectomy (LSG, 25 studies), laparoscopic Roux-en-Y gastric bypass (LRYGB, 37 studies), laparoscopic biliopancreatic diversion with duodenal switch (BPD-DS, 6 studies), laparoscopic vertical banded gastroplasty (VBG, 1 study), laparoscopic adjustable gastric banding (LAGB, 8 studies), laparoscopic banded Roux-en-Y gastric bypass (banded-GB, 2 studies), laparoscopic greater curvature plication (LGCP, 1 study), laparoscopic distal Roux-en-Y gastric bypass (distal-GB, 2 studies), laparoscopic one anastomosis gastric bypass (OAGB, 5 studies), prolonged biliopancreatic limb gastric bypass (LB-GB, 1 study), and distal one anastomosis gastric bypass (distal-OAGB, 1 study). General characteristics of included studies are presented in Table 1 with NOS quality score or Cochrane risk of bias assessments. The analyses are presented separately for each of the pre-specified periods of follow-up: 1, 2, 3, and 5 years. The number of studies included in the analysis was decreasing with the length of follow-up, as was the number of procedures studied. Results for secondary outcomes are available in supplement files. The networks of studies for each follow-up period are presented in Fig. 2.

**Table 1:** Basic characteristics of the included studies

First author	Year of publication	Country	Single/multi-center	Study type	QoL form	Study arms	Intervention type	Quality score/risk of bias
Lee [21]	2004	Taiwan	Single	RCT	GIQLI	2	VBG/LRYGB	High risk
Lee [22]	2005	Taiwan	Single	RCT	GIQLI	2	LRYGB/OAGB	High risk
Müller [23]	2008	Switzerland	Single	Cohort	SF-36, MA	2	LRYGB/LAGB	8
Campos [24]	2011	USA	Single	Cohort	BAROS	2	LAGB/LRYGB	8
Søvik [25]	2011	Norway, Sweden	Multi	RCT	SF-36	2	LRYGB/BPD-S	High risk
Alley [26]	2012	USA	Single	Cohort	BQL	2	LSG/LAGB	8
Lee [27]	2012	Taiwan	Single	Cohort	GIQLI	2	LRYGB/OAGB	8
Carlin [28]	2013	USA	Multi	Cohort	BQL	3	LRYGB/LSG/LAGB	7
Karlsen [29]	2013	Norway	Single	Cohort	SF-36, OWLQOL	2	LRYGB/LI	7
O’Brien [30]	2013	Australia	Single	RCT	SF-36	3	LAGB/crossed-over/LI	High risk
Kaseja [31]	2014	Poland	Single	Cohort	MA	2	LSG/LRYGB	8
Duarte [32]	2014	Brasil	Single	Cohort	SF-36, BAROS	3	BPD-DS/banded-GB/no intervention	6
Strain [33]	2014	USA	Multi	Cohort	IWQOL-Lite, SF-36	4	LRYGB/BPD-DS/LSG/LAGB	7
Schauer [34]	2014	USA	Single	RCT	RAND-36	3	LI/LRYGB/LSG	High risk
Bhandari [35]	2015	India	Single	Cohort	GIQLI	2	Banded-GB/LRYGB	7
Major [36]	2015	Poland	Single	Cohort	SF-36, BAROS	2	LSG/LRYGB	5
Lee [37]	2015	Taiwan	Single	Cohort	GIQLI	3	LSG/LRYGB/OAGB	6
Barr [38]	2016	USA	Single	Cohort	GERD-HRQL	2	LRYGB/LSG	7
Buzgova [39]	2016	Czech Republic	Single	Cohort	WHOQOL-BREF, HADS	2	LSG/LGCP	7
Figura [40]	2016	Germany	Single	Cohort	SF-8	2	LSG/LI	8
Risstad [41]	2016	Sweden	Multi	RCT	SF-36	2	LRYGB/distal-GB	Low risk
Ignat [42]	2016	France	Single	RCT	MA, GIQLI	2	LRYGB/LSG	High risk
Janik [43]	2016	Poland	Single	Cohort	MA	3	LRYGB/LSG/no intervention	8
Nickel [44]	2016	Germany	Single	Cohort	GIQLI	2	LRYGB/LSG	4
Omotosho [45]	2016	USA	Single	Cohort	SF-36	2	LRYGB/LI	8
Panosian [46]	2016	USA	Single	RCT	SF-36, IWQOL-Lite	2	LRYGB/LI	High risk
Accardi [47]	2017	Italy	Multi	Cohort	LQ	2	LRYGB/LAGB	5
Elrefai [48]	2017	Germany	Single	Cohort	BQL	4	LRYGB/LSG/BPD-DS/LAGB	7
Biter [49]	2017	Netherlands	Single	RCT	BAROS, SF-36	2	LSG/LRYGB	High risk
Peterli [50]	2017	Switzerland	Multi	RCT	GIQLI, BAROS	2	LSG/LRYGB	High risk
Svanevik [51]	2017	Norway	Multi	RCT	MA, OWLQOL	2	LRYGB/distal-GB	Low risk
Versteegden DPA [52]	2017	Netherlands	Single	Cohort	RAND-36	2	LSG/LRYGB	6
Schauer [53]	2017	USA	Single	RCT	RAND-36	3	LI/LRYGB/LSG	High risk
Salminen [54]	2018	Finland	Multi	RCT	MA	2	LSG/LRYGB	High risk
Homan [55]	2018	Netherlands	Single	RCT	RAND-36	2	LRYGB/LB-GB	High risk
Peterli [56]	2018	Switzerland	Multi	RCT	GIQLI	2	LSG/LRYGB	High risk
Elias [57]	2018	Sweden	Single	Cohort	SF-36	2	LRYGB/BPD-DS	7
Silva [58]	2018	Portugal	Single	Cohort	RAND-36	2	LRYGB/LSG	6
Catheline [59]	2019	France	Multi	RCT	SF-36	2	LSG/LRYGB	8
Nabil [60]	2019	Egypt	Multi	RCT	GIQLI	2	OAGB/distal-OAGB	High risk
Skogar [61]	2020	Sweden	NR	Cohort	SF-36	2	BPD-DS/LRYGB	8
Lechaux [62]	2020	France	Single	Cohort	MA	2	LSG/OAGB	5
Monpellier [63]	2020	Netherlands	Multi	Cohort	RAND-36	2	LSG/LRYGB	7
Poelemeijer [64]	2020	Netherlands	NR	Cohort	RAND-36	2	LSG/LRYGB	7

*QoL*, Quality of Life; *BQL*, Bariatric Quality of Life; *LQ*, Laval Questionnaire; *GERD-HRQL*, GERD-Health-Related Quality of Life Questionnaire; *WHOQOL-BREF*, World Health Organization Quality of Life Instruments; *SF-8*, Short Form 8 Health Survey; *BAROS*, Bariatric Analysis and Reporting Outcome System; *MA*, Moorehead-Ardelt Quality of Life Questionnaire; *GIQLI*, Gastrointestinal Quality of Life Index; *SF-36*, Short Form 36 Health Survey; *OWLQOL*, Obesity and Weight-Loss Quality of Life; *RAND-36*, Rand 36-Item Health Survey; *IWQOL-Lite*, Impact of Weight on Quality of Life-Lite; *LI*, lifestyle intervention; *LSG*, laparoscopic sleeve gastrectomy; *LRYGB*, laparoscopic Roux-en-Y gastric bypass; *BPD-DS*, laparoscopic biliopancreatic diversion with duodenal switch; *VBG*, vertical banded gastroplasty; *LAGB*, laparoscopic adjustable gastric banding; *LGCP*, laparoscopic greater curvature plication; *OAGB*, one anastomosis gastric bypass; *banded-GB*, banded laparoscopic Roux-en-Y gastric bypass; *Distal-GB*, distal laparoscopic Roux-en-Y gastric bypass; *Distal-OAGB*, distal one anastomosis gastric bypass; *LB-GB*, prolonged biliopancreatic limb gastric bypass; *RCT*, randomized controlled trial; *NR*, national registry

### Results at 1-Year Follow-up

**Fig. 3:**
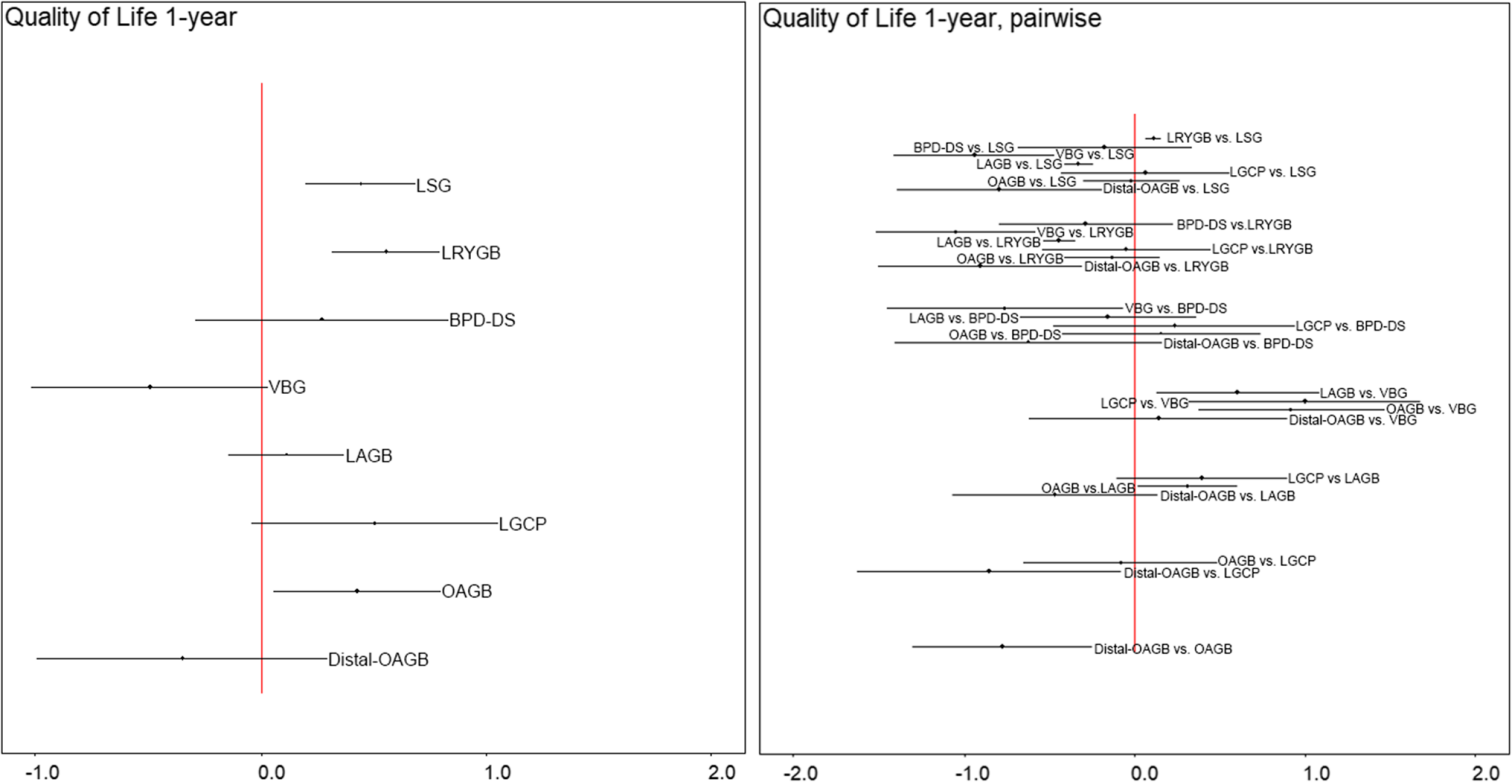
Pooled results of total HRQoL presented as SMD after 1 year **a** in comparison to lifestyle intervention; **b** pairwise comparisons between surgeries

Network meta-analysis was based on 22 studies (15 cohort studies and 7 RCTs) comparing 8 different surgical techniques (LSG, LRYGB, BPD-DS, VBG, LAGB, LGCP, OAGB, and distal-OAGB) (Fig. 3, Table 2). The analysis showed significant difference in HRQoL in favor of LSG, LRYGB, and OAG compared with lifestyle intervention (SMD: 0.44; 95% CrI 0.2 to 0.68 for LSG, SMD: 0.56; 95% CrI 0.31 to 0.8 for LRYGB, and SMD: 0.43; 95% CrI 0.06 to 0.8 for OAGB) and no significant effect for the remaining procedures. Pairwise comparisons showed a significant difference in HRQOL in favor of LRYGB vs. LSG (SMD: 0.11; 95% CrI 0.07 to 0.16), while VBG, LAGB, and distal-OAGB had significantly lower HRQoL than LSG and LRYGB; however, the difference between LRYGB and LSG was insignificant clinically (MCID < 5) (Table 3). In a detailed analysis of the physical aspect, apart from LAGB and LGCP, surgical interventions led to better HRQoL than lifestyle intervention (supplementary file 4). With regard to specific HRQoL domains, pairwise comparisons showed that LAGB was inferior to lifestyle intervention in physical domain and general health perceptions domain of HRQOL after 1 year, while LSG, LRYGB, BPD-DS, and OAGB were associated with better HRQoL in general health perception domain than control (supplementary file 4). Detailed information on the remaining aspects of the QoL is shown in supplementary files. Visual assessment of the funnel plot for LSG vs. LRYGB showed limited publication bias.

**Table 2:**
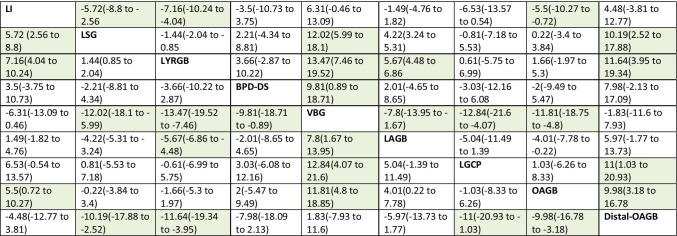
HRQoL after 1 year presented GIQLI scale (0–144)

MD > 0 favors intervention in row; MD < 0 favors intervention in column; MCID > 5 marked green

*HRQoL*, health-related quality of life; *LI*, lifestyle intervention; *LSG*, laparoscopic sleeve gastrectomy; *LRYGB*, laparoscopic Roux-en-Y gastric bypass; *Distal-OAGB*, distal one anastomosis gastric bypass; *BPD-DS*, laparoscopic biliopancreatic diversion with duodenal switch; *VBG*, vertical banded gastroplasty; *LAGB*, laparoscopic adjustable gastric banding; *LGCP*, laparoscopic greater curvature plication; *OAGB*, one anastomosis gastric bypass

**Table 3:**
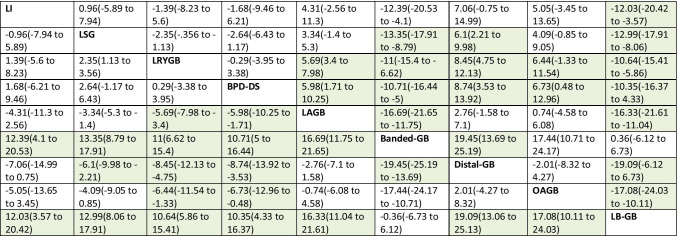
HRQoL after 2 years presented GIQLI scale (0–144)

MD > 0 favors intervention in row; MD < 0 favors intervention in column; MCID > 5 marked green

*HRQoL*, health-related quality of life; *LI*, lifestyle intervention; *LSG*, laparoscopic sleeve gastrectomy; *LRYGB*, laparoscopic Roux-en-Y gastric bypass; *BPD-DS*, laparoscopic biliopancreatic diversion with duodenal switch; *LAGB*, laparoscopic adjustable gastric banding; *OAGB*, one anastomosis gastric bypass; *banded-GB*, banded Roux-en-Y gastric bypass; *Distal-GB*, distal Roux-en-Y gastric bypass; *LB-GB*, prolonged biliopancreatic limb gastric bypass

### Results at 2-Year Follow-up

**Fig. 4:**
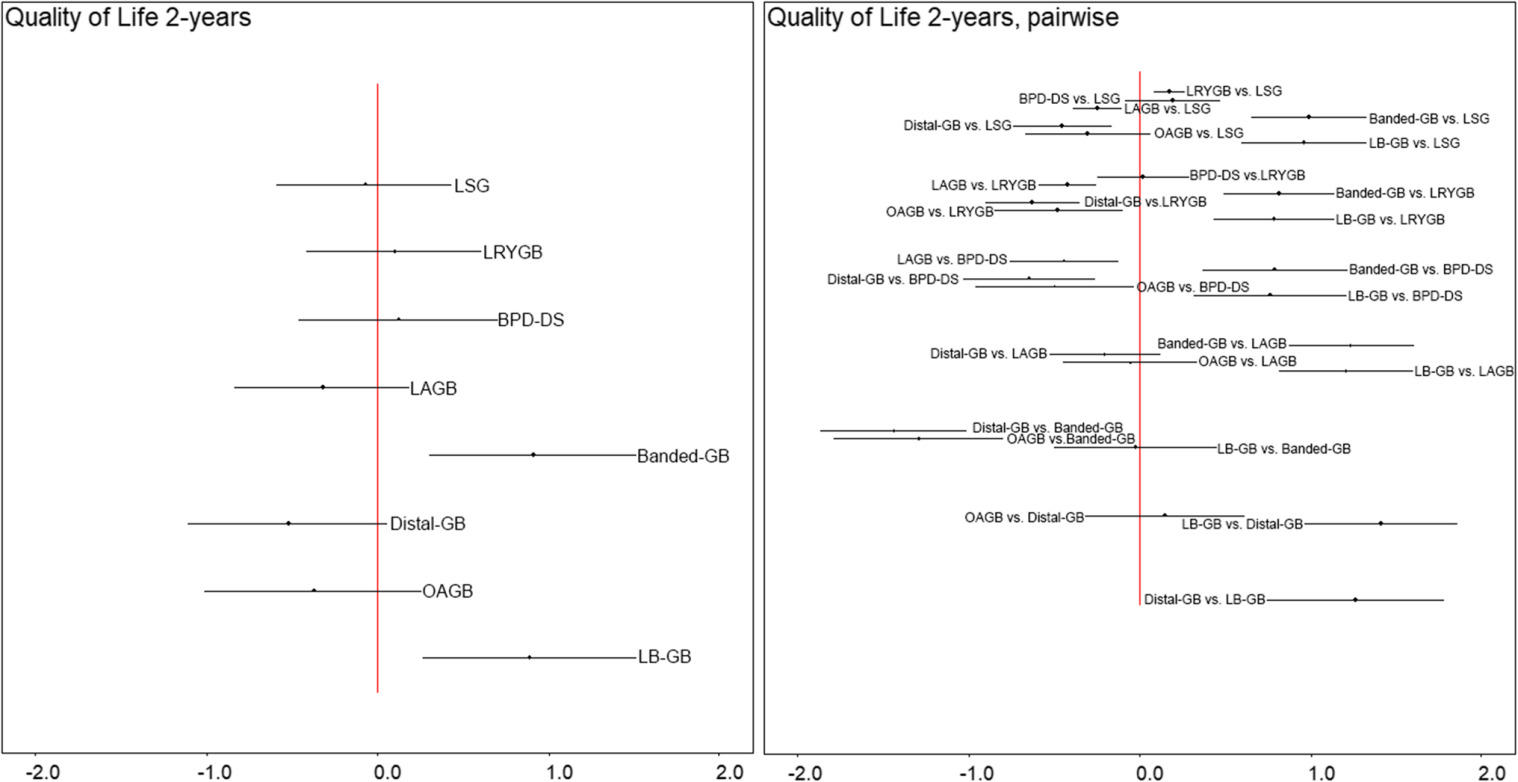
Pooled results of total HRQoL presented as SMD at 2 years **a** in comparison to lifestyle intervention; **b** pairwise comparisons between surgeries

Network meta-analysis was based on 15 studies (7 cohort studies and 8 RCTs), involving 8 bariatric procedures (LSG, LRYGB, BPD-DS, LAGB, banded-GB, distal-GB, OAGB, and LB-GB) (Fig. 4, Table 3). When compared with lifestyle intervention, only banded-GB and LB-GB had significantly better HRQoL at 2 years (SMD: 0.92; 95% CrI 0.3 to 1.52 for banded-GB and SMD: 0.89; 95% CrI 0.26 to 1.51). In pairwise comparisons, LRYGB was associated with better HRQoL than LSG, however, clinically not relevant (MCID < 5). Distal-GB was associated with worse HRQoL compared to standard LRYGB, whereas LB-GB and banded-GB modifications were associated with better. LAGB was associated with worse HRQoL than LSG, LRYGB, BPD-DS, banded-GB, and LB-GB. Detailed information on specific aspects of HRQoL is available in supplementary files.

### Results at 3-Year Follow-up

Network meta-analysis was based on 9 studies (6 cohort studies, 4 RCTs) involving 5 different surgical procedures (LSG, LRYGB, BPD-DS, LAGB, banded-GB). LSG, LRYGB, BPD-DS, and LAGB showed better HRQoL than lifestyle intervention (SMD: 0.9; 95% CrI 0.58 to 1.23, SMD: 0.96; 95% CrI 0.65 to 1.29, SMD: 1.16; 95% CrI 0.45 to 1.87, SMD: 0.78; 95% CrI 0.4 to 1.17, respectively, all with MCID > 5), while no significant differences were found for the remaining procedure in comparison with control (supplementary file 3).

### Results at 5-Year Follow-up

Network meta-analysis was based on 7 studies (3 cohort studies, 4 RCTs) involving 4 different surgical procedures (LSG, LRYGB, BPD-DS, and OAGB). All interventions showed better HRQoL in comparison to control (SMD: 0.92; 95% CrI 0.58 to 1.26, SMD: 1.27; 95% CrI 0.94 to 1.61, SMD: 1.43; 95% CrI 1 to 1.87, and SMD: 1.01; 95% CrI 0.63 to 1.4, respectively) . Pairwise comparisons showed that both LRYGB and BPD-DS had better HRQoL than LSG and OAGB, with no difference between LRYGB and BPD-DS (supplementary file 3).

## Discussion

To our knowledge, this network meta-analysis is the first to attempt to summarize and compare HRQoL after different bariatric procedures in patients with severe obesity. In total, we included 47 studies with 26,629 patients and 11 different surgical techniques covering the follow-up period from 1 to 5 years. Our analysis included both RCTs and observational studies to assess a wide number of different interventions as possible. Short-term results (1 year) showed that only LSG, LRYGB, and OAGB offer better QoL in comparison to non-surgical interventions, with LRYGB showing better results than LSG. Pairwise comparisons showed that LAGB and VBG result in worse HRQoL in comparison to LSG or LRYGB. Medium-term results (2- years) showed that patients who received banded-GB reported better HRQoL improvement than non-surgical patients, while LAGB resulted in worse results than other techniques, excluding OAGB and distal-GB. Long-term results (3 and 5 years) showed that LRYGB and LSG maintain HRQoL after surgery. BPD-DS at 5 years showed significant improvement to control, which is in contrast to previous years.

Previous network meta-analysis by Park et al. compared weight loss and remission of comorbidities following various bariatric procedures; however, this study did not explore the effects of those interventions on HRQoL [65]. The only meta-analysis which focused on HRQoL after bariatric surgery contained only pairwise comparisons and compared bariatric with non-bariatric patients showing an improvement in the HRQoL, mainly in the domain of physical functioning and activity [66]. Previous pairwise comparison by Hu et al. showed no statistically significant differences in HRQoL between LSG and LRYGB [67]. In general, LSG and LRYGB are the most commonly performed types of surgery worldwide, which is also represented in the number of studies in our review comparing these two techniques. Even though both techniques are well established and have been performed for several years, the debate on which method is better is ongoing, with both pros and cons for each one. In recent years, this resulted in conducting several RCTs, including the SLEEVEPASS (5-year results) and SM-BOSS (5-year results) showing no differences in HRQoL between those two procedures [54, 56, 68]. Our analysis found that both LSG and LRYGB in the long term (3–5 years) are associated with better HRQoL than no surgical intervention. Pairwise comparisons show that LAGB and VBG will likely result in worse HRQoL than other techniques, which is consistent with previous literature [69]. The present study suggests that LAGB may worsen HRQoL in comparison to LSG, LRYGB, or BPD-DS. Finally, with the current evidence, it is unclear whether BPD-DS in short term either improves or worsens HRQoL after the surgery. This may be associated with malnutrition and a more demanding diet than other techniques [70]. Results from a 5-year follow-up demonstrate that surgical interventions such as LSG, LRYGB, BPD-DS, and OAGB provide better HRQoL as compared to non-surgical methods.

One of the advantages of this network meta-analysis is the comparison of different variations of gastric bypass. In general, the most commonly performed bypass is LRYGB and OAGB. In our review, we compared other less common versions such as banded gastric bypass (elastic band placed on the pouch), distal gastric bypass (long alimentary limb), long biliopancreatic limb gastric bypass, and distal-OAGB (long biliopancreatic limb). Our results show that banded-GB and LB-GB sometimes are associated with better HRQoL than standard LRYGB, whereas distal-GB did not improve HRQoL. A systematic review by Shoar et al. showed that although banded-GB is associated with higher weight loss, it came with the expense of a higher incidence of food intolerance and postoperative vomiting, which can impact HRQoL [71]. The weight loss outcomes are similar for LRYGB and LB-GB. However, at 2 years, LB-GB was associated with better HRQoL than LRYGB [55, 72–74]. OAGB is still a controversial method with limited literature on long-term effectiveness [75–78]. The main modifications of this technique include a different length of the biliopancreatic limb [60]. The analysis of pooled data in the review showed that variation with the longer biliopancreatic limb in OAGB is associated with worse HRQoL in comparison to standard OAGB.

BPD-DS as a technique that alters the gastrointestinal tract in the greatest fashion requires more time for patients to adjust to new dietary patterns or the need for proper vitamin supplementation. Strain’s et al. study demonstrated that patients’ HRQoL improves after BPD-DS in the long run (9-year follow-up) [79]. BPD-DS in our analysis showed better HRQoL improvement than LSG.

This systematic review is the first comprehensive analysis of the impact of different bariatric procedures on HRQoL. Although a multi-arm RCT would be a preferable choice to establish which method is better, the task at hand would be very difficult to perform as most bariatric surgeons do not perform limited types of bariatric procedures in their elective practice. This network meta-analysis demonstrates that the most commonly performed surgeries, such as LSG and LRYGB, are associated with better HRQoL. It also demonstrates that some novel techniques, such as LB-GB, are worth investigating to a greater extent, whereas others (distal-OAGB) are less promising from an HRQoL standpoint. Finally, this meta-analysis showed that LAGB is associated with worse QoL, which cements LAGB as the least favorable bariatric procedure in all aspects.

### Limitations

The main limitation of this study is the heterogeneity between included papers, such as different study designs including RCT and non-RCT, not homogenous population in terms of BMI or comorbidities, and different HRQoL instruments used. We decided to include non-RCTs, such as cohort studies to enable comparing as many different interventions as possible. We used the standardized mean difference to combine the results from different instruments, but to make the results more friendly we converted them into the GIQLI scale, which is widely used in bariatric surgery studies. The number of studies and number of compared interventions decreased with the longevity of follow-up. Another factor that needs to be considered is the underrepresentation of some of the procedures such as distal LRYGB, whereas LSG and LRYGB are the most common procedures analyzed in this review. Another limitation of the study is the quality of the included studies, although observational studies were considered to be of moderate quality in general, their design is associated with lower confidence in estimates as compared with RCT, while the majority of included RCTs was of high risk of bias. We have not searched for unpublished studies, this may have introduced potential publication bias. Formal testing of pub bias was not feasible due to the low number of studies for many comparisons. Nonetheless, this is a unique comparison of the different bariatric procedures and some compromises were required to achieve it.

## Conclusion

This is the first network meta-analysis comparing HRQoL after different bariatric procedures. It demonstrates LSG and LRYGB may lead to better HRQoL across most follow-up time points. Long-term analysis shows that bariatric intervention results in better HRQoL than non-surgical interventions. Our analysis indicates that some procedures such as VBG or LAGB may lead to worse HRQoL. Future studies comparing different types of bariatric procedures should include HRQoL-related measures to their list of outcomes besides weight loss, comorbidities, and complications to provide a holistic perspective of each procedure.

## Supplementary Information

Below is the link to the electronic supplementary material.

Supplementary file1 (PNG 29 KB)

Supplementary file2 (PDF 290 KB)

Supplementary file3 (PDF 587 KB)

Supplementary file4 (PDF 1468 KB)

## References

[1] Rajan TM, Menon V (2017). Psychiatric disorders and obesity: a review of association studies. J Postgrad Med.

[2] Fiorani C, et al. Long-term quality of life outcomes after laparoscopic sleeve gastrectomy and Roux-en-Y gastric bypass-a comparative study*. *Obes Surg. 2020. 10.1007/s11695-020-05049-3 PMC7920888 33064260

[3] Ware JE, Sherbourne CD (1992). The MOS 36-item short-form health survey (SF-36). I. Conceptual framework and item selection. Med Care.

[4] Moorehead MK (2003). The validation of the Moorehead-Ardelt Quality of Life Questionnaire II. Obes Surg.

[5] Eypasch E (1995). Gastrointestinal Quality of Life Index: development, validation and application of a new instrument. Br J Surg.

[6] Kolotkin RL (1995). Assessing Impact of Weight on Quality of Life. Obes Res.

[7] Hutton B (2015). The PRISMA extension statement for reporting of systematic reviews incorporating network meta-analyses of health care interventions: checklist and explanations. Ann Intern Med.

[8] Weiner S (2005). The Bariatric Quality of Life index: a measure of well-being in obesity surgery patients. Obes Surg.

[9] Therrien F (2011). The Laval Questionnaire: a new instrument to measure quality of life in morbid obesity. Health Qual Life Outcomes.

[10] Velanovich V (2007). The development of the GERD-HRQL symptom severity instrument. Dis Esophagus.

[11] The WHOQOL Group (1998). Development of the World Health Organization WHOQOL-BREF quality of life assessment. Psychol Med.

[12] Yiengprugsawan V, Kelly M, Tawatsupa B, Michalos AC (2014). SF-8TM Health Survey. Encyclopedia of quality of life and well-being research.

[13] Oria HE, Moorehead MK (1998). Bariatric analysis and reporting outcome system (BAROS). Obes Surg.

[14] Niero M (2002). A new approach to multicultural item generation in the development of two obesity-specific measures: the Obesity and Weight Loss Quality of Life (OWLQOL) questionnaire and the Weight-Related Symptom Measure (WRSM). Clin Ther.

[15] Fu R, et al. Handling continuous outcomes in quantitative synthesis. In Methods guide for effectiveness and comparative effectiveness reviews. AHRQ Publication: Oregon; 2013.

[16] Wells G, Shea B, O’Connell J. The Newcastle-Ottawa Scale (NOS) for Assessing The Quality of Nonrandomised Studies in Meta-analyses*.* Ottawa Health Research Institute Web site; 2014. **7**.

[17] Higgins JP (2011). The Cochrane Collaboration’s tool for assessing risk of bias in randomised trials. BMJ.

[18] Dias S, et al. Network meta-analysis for decision making. 2018.

[19] Thorlund K (2011). Pooling health-related quality of life outcomes in meta-analysis-a tutorial and review of methods for enhancing interpretability. Res Synth Methods.

[20] Shi HY (2008). Responsiveness and minimal clinically important differences after cholecystectomy: GIQLI versus SF-36. J Gastrointest Surg.

[21] Lee WJ (2004). Laparoscopic vertical banded gastroplasty and laparoscopic gastric bypass: a comparison. Obes Surg.

[22] Lee W-J (2005). Laparoscopic Roux-en-Y versus mini-gastric bypass for the treatment of morbid obesity: a prospective randomized controlled clinical trial. Ann Surg.

[23] Muller M (2008). Quality of life after bariatric surgery-a comparative study of laparoscopic banding vs. bypass. Obes Surg.

[24] Campos GM (2011). Better weight loss, resolution of diabetes, and quality of life for laparoscopic gastric bypass vs banding: results of a 2-cohort pair-matched study. Arch Surg (Chicago, Ill.: 1960).

[25] Svanevik M (2018). Patient-reported outcome measures 2 years after standard and distal gastric bypass—a double-blind randomized controlled trial. Obes Surg.

[26] Alley JB (2012). Quality of life after sleeve gastrectomy and adjustable gastric banding. Surg Obes Relat Dis.

[27] Lee WJ (2012). Laparoscopic Roux-en-Y vs. mini-gastric bypass for the treatment of morbid obesity: a 10-year experience. Obes Surg.

[28] Carlin AM (2013). The comparative effectiveness of sleeve gastrectomy, gastric bypass, and adjustable gastric banding procedures for the treatment of morbid obesity. Ann Surg.

[29] Karlsen TI, et al. Health related quality of life after gastric bypass or intensive lifestyle intervention: a controlled clinical study*.* Health Qual Life Outcomes. 2013; 11(1). 10.1186/1477-7525-11-17 PMC3599616 23406190

[30] O'Brien PE (2013). Intensive medical weight loss or laparoscopic adjustable gastric banding in the treatment of mild to moderate obesity: long-term follow-up of a prospective randomised trial. Obes Surg.

[31] Kaseja K, Majewski WD, Kolpiewicz B (2014). A comparison of efectiveness, and an assessment of the quality of life of patients after laparoscopic sleeve gastrectomy and Roux-en-Y gastric bypass. Ann Acad Med Stetin.

[32] Duarte MIXDT (2014). Impact on quality of life, weight loss and comorbidities: a study comparing the biliopancreatic diversion with duodenal switch and the banded Roux-en-Y gastric bypass. Arq Gastroenterol.

[33] Strain GW (2014). The effects of weight loss after bariatric surgery on health-related quality of life and depression. Nutr Diabetes.

[34] Schauer P (2014). Bariatric surgery versus intensive medical therapy for diabetes - 3-year outcomes. N Engl J Med.

[35] Bhandari M (2016). Comparison between banded and nonbanded Roux-en-Y gastric bypass with 2-year follow-up: a preliminary retrospective analysis. Obes Surg.

[36] Major P (2015). Quality of life after bariatric surgery. Obes Surg.

[37] Lee WJ (2015). Medium-term results of laparoscopic sleeve gastrectomy: a matched comparison with gastric bypass. Obes Surg.

[38] Barr AC (2017). GERD and acid reduction medication use following gastric bypass and sleeve gastrectomy. Surg Endosc.

[39] Bužgová R (2016). Evaluation of quality of life, clinical parameters, and psychological distress after bariatric surgery: comparison of the laparoscopic sleeve gastrectomy and laparoscopic greater curvature plication. Bariatr Surg Pract Patient Care.

[40] Figura A (2017). Improvement in self-reported eating-related psychopathology and physical health-related quality of life after laparoscopic sleeve gastrectomy: a pre-post analysis and comparison with conservatively treated patients with obesity. Eat Behav.

[41] Risstad H (2016). Standard vs distal Roux-en-Y gastric bypass in patients with body mass index 50 to 60: a double-blind, randomized clinical trial. JAMA Surg.

[42] Ignat M (2017). Randomized trial of Roux-en-Y gastric bypass versus sleeve gastrectomy in achieving excess weight loss. Br J Surg.

[43] Janik MR (2016). Quality of life and bariatric surgery: cross-sectional study and analysis of factors influencing outcome. Obes Surg.

[44] Nickel F (2017). Gastrointestinal quality of life improves significantly after sleeve gastrectomy and Roux-en-Y gastric bypass-a prospective cross-sectional study within a 2-year follow-up. Obes Surg.

[45] Omotosho P (2016). Gastric bypass significantly improves quality of life in morbidly obese patients with type 2 diabetes. Surg Endosc.

[46] Panosian J (2017). Physical activity in obese type 2 diabetes after gastric bypass or medical management. Am J Med.

[47] Accardi R (2017). Different quality of life outcomes between Roux-en-Y gastric bypass and laparoscopic adjustable gastric banding. Bariatr Surg Pract Patient Care.

[48] Elrefai M (2017). Quality of life after bariatric surgery: comparison of four different surgical procedures. Bariatr Surg Pract Patient Care.

[49] Biter LU (2017). Quality of life 1 year after laparoscopic sleeve gastrectomy versus laparoscopic Roux-en-Y gastric bypass: a randomized controlled trial focusing on gastroesophageal reflux disease. Obes Surg.

[50] Peterli R (2017). Laparoscopic sleeve gastrectomy versus Roux-Y-gastric bypass for morbid obesity-3-year outcomes of the prospective randomized Swiss Multicenter Bypass Or Sleeve Study (SM-BOSS). Ann Surg.

[51] Svanevik M (2018). Patient-reported outcome measures 2 years after standard and distal gastric bypass-a double-blind randomized controlled trial. Obes Surg.

[52] Versteegden DPA, Van Himbeeck MJJ, Nienhuijs SW (2018). Improvement in quality of life after bariatric surgery: sleeve versus bypass. Surg Obes Relat Dis.

[53] Schauer PR (2017). Bariatric surgery versus intensive medical therapy for diabetes - 5-year outcomes. N Engl J Med.

[54] Salminen P (2018). Effect of laparoscopic sleeve gastrectomy vs laparoscopic Roux-en-Y gastric bypass on weight loss at 5 years among patients with morbid obesity: the SLEEVEPASS randomized clinical trial. JAMA.

[55] Homan J (2018). A longer biliopancreatic limb in Roux-en-Y gastric bypass improves weight loss in the first years after surgery: results of a randomized controlled trial. Obes Surg.

[56] Peterli R (2018). Effect of laparoscopic sleeve gastrectomy vs laparoscopic Roux-en-Y gastric bypass on weight loss in patients with morbid obesity: the SM-BOSS Randomized Clinical Trial. JAMA.

[57] Elias K (2018). Changes in bowel habits and patient-scored symptoms after Roux-en-Y gastric bypass and biliopancreatic diversion with duodenal switch. Surg Obes Relat Dis.

[58] Silva JN (2018). How is bariatric surgery improving the quality of life of obese patients: a Portuguese cross-sectional study. Acta Med Port.

[59] Catheline JM (2019). Prospective, multicentric, comparative study between sleeve gastrectomy and Roux-en-Y gastric bypass, 277 patients, 3 years follow-up. J Visc Surg.

[60] Nabil TM (2019). Conventional versus distal laparoscopic one-anastomosis gastric bypass: a randomized controlled trial with 1-year follow-up. Obes Surg.

[61] Skogar ML, Sundbom M. Early complications, long-term adverse events, and quality of life after duodenal switch and gastric bypass in a matched national cohort*.* Surg Obes Relat Dis. 2020. 10.1016/j.soard.2020.02.001 32156633

[62] Lechaux D, Le Foll D, Rascle O. Weight loss and quality of life after sleeve gastrectomy or one-anastomosis gastric bypass: results of a prospective study of 120 women with morbid obesity*.* Obes Surg. 2020. 10.1007/s11695-020-04442-2 32034619

[63] Monpellier VM, et al. Health-related quality of life after sleeve gastrectomy equal to Roux-en-Y gastric bypass patients? Qual Life Res. 2020. 10.1007/s11136-020-02449-x 32152816

[64] Poelemeijer YQM, et al. Measuring quality of life in bariatric surgery: a multicentre study*.* Surg Endosc. 2020. 10.1007/s00464-019-07350-4 PMC7644534 31993820

[65] Park CH (2019). Comparative efficacy of bariatric surgery in the treatment of morbid obesity and diabetes mellitus: a systematic review and network meta-analysis. Obes Surg.

[66] Lindekilde N (2015). The impact of bariatric surgery on quality of life: a systematic review and meta-analysis. Obes Rev.

[67] Hu Z (2020). A comprehensive comparison of LRYGB and LSG in obese patients including the effects on QoL, comorbidities, weight loss, and complications: a systematic review and meta-analysis. Obes Surg.

[68] Lee Y, et al. Laparoscopic sleeve gastrectomy versus laparoscopic Roux-en-Y gastric bypass: a systematic review and meta-analysis of weight loss, comorbidities, and biochemical outcomes from randomized controlled trials*.* Ann Surg. 2019. 10.1097/SLA.0000000000003671 31693504

[69] Campos GM (2011). Better weight loss, resolution of diabetes, and quality of life for laparoscopic gastric bypass vs banding: results of a 2-cohort pair-matched study. Arch Surg.

[70] Homan J (2018). Treatment of vitamin and mineral deficiencies after biliopancreatic diversion with or without duodenal switch: a major challenge. Obes Surg.

[71] Shoar S (2019). Banded versus nonbanded Roux-en-Y gastric bypass: a systematic review and meta-analysis of randomized controlled trials. Surg Obes Relat Dis.

[72] Boerboom A (2019). A long biliopancreatic and short alimentary limb results in more weight loss in revisional RYGB surgery. Outcomes of the randomized controlled ELEGANCE REDO trial. Surg Obes Relat Dis.

[73] Boerboom A (2019). A long biliopancreatic and short alimentary limb results in more weight loss in revisional RYGB surgery. Outcomes of the randomized controlled ELEGANCE REDO trial. Surg Obes Relat Dis.

[74] Moon RC (2020). Short-term results of long biliopancreatic limb Roux-en-Y gastric bypass-is it superior?. Surg Obes Relat Dis.

[75] Rutledge R, Kular K, Manchanda N (2019). The mini-gastric bypass original technique. Int J Surg.

[76] Poublon N, et al. One anastomosis gastric bypass vs. Roux-en-Y gastric bypass, remedy for insufficient weight loss and weight regain after failed restrictive bariatric surgery*. *Obes Surg. 2020. 10.1007/s11695-020-04536-x PMC7378100 32307669

[77] Ramos AC (2020). IFSO (International Federation for Surgery of Obesity and Metabolic Disorders) Consensus Conference Statement on One-Anastomosis Gastric Bypass (OAGB-MGB): results of a modified Delphi study. Obes Surg.

[78] Robert M (2019). Efficacy and safety of one anastomosis gastric bypass versus Roux-en-Y gastric bypass for obesity (YOMEGA): a multicentre, randomised, open-label, non-inferiority trial. Lancet.

[79] Strain GW (2017). The impact of biliopancreatic diversion with duodenal switch (BPD/DS) over 9 years. Obes Surg.

