# Supplementary figures and images for: Quality of Life After Bariatric Surgery—a Systematic Review with Bayesian Network Meta-analysis
Source: Obes Surg. 2021 Oct 11;31(12):5213–23. doi: 10.1007/s11695-021-05687-1 (PMC8595157; doi:10.1007/s11695-021-05687-1)

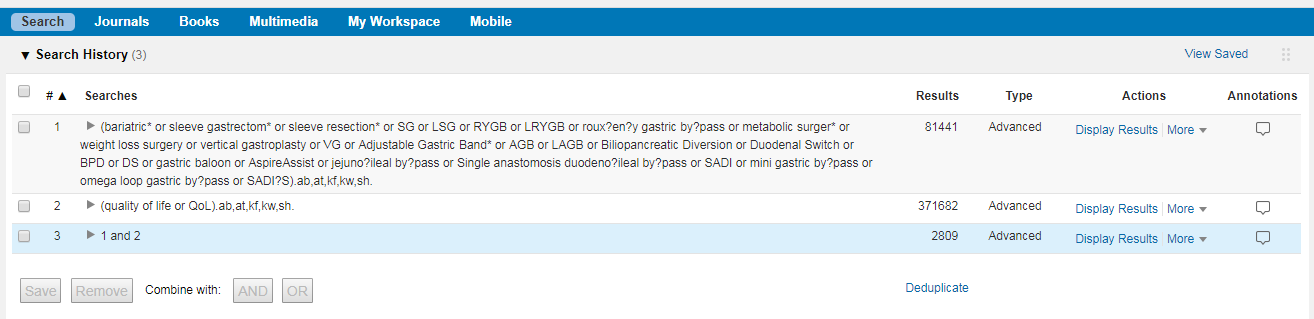

Supplement: Supplementary file 1 — Supplementary file1 (PNG 29 KB) [file 11695_2021_5687_MOESM1_ESM.png]
