# Supplementary material for: Quality of Life After Bariatric Surgery—a Systematic Review with Bayesian Network Meta-analysis
Source: Obes Surg. 2021 Oct 11;31(12):5213–23. doi: 10.1007/s11695-021-05687-1 (PMC8595157; doi:10.1007/s11695-021-05687-1)
Supplement: Supplementary file 2 — Supplementary file2 (PDF 290 KB) [file 11695_2021_5687_MOESM2_ESM.pdf]

This code is part of Dias, Ades, Welton, Jansen and Sutton (2018) Network Meta-Analysis for Decision Making.
This work should be cited whenever the code is used whether in its standard form or adapted.

```
# Normal likelihood, identity link
# Trial-level data given as treatment differences
# Random effects model for multi-arm trials
model{                               # *** PROGRAM STARTS
for(i in 1:ns2) {                    # LOOP THROUGH 2-ARM STUDIES
    y[i,2] ~ dnorm(delta[i,2],prec[i,2]) # normal likelihood for 2-arm
trials
#Deviance contribution for trial i
    resdev[i] <- (y[i,2]-delta[i,2])*(y[i,2]-delta[i,2])*prec[i,2]
  }
for(i in (ns2+1):(ns2+ns3)) {        # LOOP THROUGH THREE-ARM STUDIES
    for (k in 1:(na[i]-1)) {    # set variance-covariance matrix
        for (j in 1:(na[i]-1)) {
            Sigma[i,j,k] <- V[i]*(1-equals(j,k)) + var[i,k+1]*equals(j,k)
          }
      }
    Omega[i,1:(na[i]-1),1:(na[i]-1)] <- inverse(Sigma[i,,])  #Precision
matrix
# multivariate normal likelihood for 3-arm trials
    y[i,2:na[i]] ~ dmnorm(delta[i,2:na[i]],Omega[i,1:(na[i]-1),1:(na[i]-
1)])
#Deviance contribution for trial i
    for (k in 1:(na[i]-1)){  # multiply vector & matrix
        ydiff[i,k]<- y[i,(k+1)] - delta[i,(k+1)]
        z[i,k]<- inprod2(Omega[i,k,1:(na[i]-1)], ydiff[i,1:(na[i]-1)])
              }
    resdev[i]<- inprod2(ydiff[i,1:(na[i]-1)], z[i,1:(na[i]-1)])
  }
for(i in 1:(ns2+ns3)){                         #   LOOP THROUGH ALL STUDIES
    w[i,1] <- 0    # adjustment for multi-arm trials is zero for control
arm
    delta[i,1] <- 0             # treatment effect is zero for control arm
    for (k in 2:na[i]) {             #  LOOP THROUGH ARMS
        var[i,k] <- pow(se[i,k],2)   # calculate variances
        prec[i,k] <- 1/var[i,k]      # set precisions
      }
    for (k in 2:na[i]) {             # LOOP THROUGH ARMS
# trial-specific RE distributions
        delta[i,k] ~ dnorm(md[i,k],taud[i,k])
# mean of random effects distributions, with multi-arm trial correction
        md[i,k] <-  d[t[i,k]] - d[t[i,1]] + sw[i,k]
# precision of random effects distributions (with multi-arm trial
correction)
        taud[i,k] <- tau *2*(k-1)/k
# adjustment, multi-arm RCTs
        w[i,k] <- (delta[i,k] - d[t[i,k]] + d[t[i,1]])
# cumulative adjustment for multi-arm trials
        sw[i,k] <- sum(w[i,1:k-1])/(k-1)
      }
  }
totresdev <- sum(resdev[])            #Total Residual Deviance
d[1]<-0       # treatment effect is zero for reference treatment
# vague priors for treatment effects
for (k in 2:nt){  d[k] ~ dnorm(0,.0001) }
sd ~ dunif(0,5)     # vague prior for between-trial SD
tau <- pow(sd,-2)   # between-trial precision = (1/between-trial variance)
# pairwise mean differences for all possible pair-wise comparisons
for (c in 1:(nt-1)) {
   for (k in (c+1):nt)  { diff[c,k] <- d[k]-d[c] }
```

```
 }
# ranking on relative scale
for (k in 1:nt) {
#  rk[k] <- nt+1-rank(d[],k)  # assumes higher values are "good"
  rk[k] <- rank(d[],k)        # assumes higher values are "bad"
   best[k] <- equals(rk[k],1) #calculate probability that treat k is best
# calculates probability that treat k is h-th best
  for (h in 1:nt){  prob[h,k] <- equals(rk[k],h)  }
 }
# Provide estimates of treatment effects T[k] on the natural scale
# Given a Mean Effect, meanA, for 'standard' treatment A,
# with precision (1/variance) precA
A ~ dnorm(meanA,precA)
for (k in 1:nt) { T[k] <- A + d[k]  }
}                                      # *** PROGRAM ENDS
```
