# Supplementary material for: Quality of Life After Bariatric Surgery—a Systematic Review with Bayesian Network Meta-analysis
Source: Obes Surg. 2021 Oct 11;31(12):5213–23. doi: 10.1007/s11695-021-05687-1 (PMC8595157; doi:10.1007/s11695-021-05687-1)
Supplement: Supplementary file 3 — Supplementary file3 (PDF 587 KB) [file 11695_2021_5687_MOESM3_ESM.pdf]

Basic characteristics of the patients in the studies

First Author	Intervention A/B/C/D	BMI A	BMI B	BMI C	BMI D	DM A	DM B	DM C	DM D	OSAS A	OSAS B	OSAS C	OSAS D	HT A	HT B	HT C	HT D
Lee WJ [41]	VBG/LRYGB	43.14	43.18	-	-	ND	ND	-	-	ND	ND	-	-	ND	ND	-	-
Lee WJ [42]	LRYGB/OAGB	43.8	44.8	-	-	ND	ND	-	-	ND	ND	-	-	ND	ND	-	-
Müller MK [43]	LRYGB/LAGB	45.7	45.3	-	-	17.31%	13.46%	-	-	ND	ND	-	-	ND	ND	-	-
Campos GM [44]	LAGB/LYRGB	45.7	46	-	-	40%	42%	-	-	12%	19%	-	-	57%	63%	-	-
Søvik TT [45]	LRYGB/BPD-DS.	54.8	55.2	-	-	16.13%	20.69%	-	-	ND	ND	-	-	25.81%	27.59%	-	-
Alley JB [46]	LSG/LAGB	42.7	41.9	-	-	ND	ND	-	-	ND	ND	-	-	ND	ND	-	-
Lee WJ [47]	LRYGB/OAGB	40.5	41.1	-	-	ND	ND	-	-	ND	ND	-	-	ND	ND	-	-
Carlin AM [48]	LRYGB/LSG/LAGB	47	48	48	-	0.0102	0.0105	0.0102	-	0.0149	0.0149	0.0149	-	0.0176	0.018	0.018	-
Karlsen TI [49]	LRYGB/LI	46	43	-	-	ND	ND	-	-	ND	ND	-	-	ND	ND	-	-
O'Brien PE [50]	LAGB/LI	33.62	33.19	-	-	100%	100%	-	-	ND	ND	-	-	ND	ND	-	-
Kaseja K [51]	LRYGB/LSG	45.1	46.5	-	-	ND	ND	-	-	ND	ND	-	-			-	-
Duarte MIX [52]	BPD-DS/banded-GB	138.8 kg	121.65 kg	-	-	47.06%	55%	-	-	52.90%	65%	-	-	64.71%	85%	-	-
Strain GW [53]	LRYGB/BPD-DS/LAGB/LSG	46.6	56.9	45.3	58	ND	ND	ND	ND	ND	ND	ND	ND	ND	ND	ND	ND
Schauer PR	LI/LRYGB/LSG	36.4	37.1	36.1	-	97.50%	97.92%	97.96%	-	ND	ND	ND	-	ND	ND	ND	-

[54]																	
Bhandari M [55]	LRYGB/banded-GB	42.16	44.13	-	-	55.45%	60.94%	-	-	63.37%	57.81%	-	-	61.39%	67.19%	-	-
Major P [56]	LSG/LRYGB	49.98	50.72	-	-	ND	ND	-	-	ND	ND	-	-	ND	ND	-	-
Lee WJ [57]	LSG/LRYGB/OAGB	37.5	37.5	37.4	-	ND	ND	ND	-	ND	ND	ND	-	ND	ND	ND	-
Barr AC [58]	LRYGB/LSG	47	49.1	-	-	ND	ND	-	-	ND	ND	-	-	ND	ND	-	-
Buzgova R [59]	LSG/LGCP	42.8	42.4	-	-	ND	ND	-	-	ND	ND	-	-	ND	ND	-	-
Figura A [60]	LSG/LI	51.5	40.3	-	-	ND	ND	-	-	ND	ND	-	-	ND	ND	-	-
Risstad H [61]	LRYGB/dista-GB	54.5	55.3	-	-	16.13%	20.68%	-	-	16.13%	20.68%	-	-	25.81%	27.59%	-	-
Ignat M [62]	LRYGB/LSG	47	45.5	-	-	ND	ND	-	-	ND	ND	-	-	ND	ND	-	-
Janik MR [63]	LSG/LRYGB	47.1	38.1	-	-	7.14%	30%	-	-	ND	ND	-	-	32.14%	33.30%	-	-
Nickel F [64]	LSG/LRYGB	ND	ND	-	-	ND	ND	-	-	ND	ND	-	-	ND	ND	-	-
Omotosho P [65]	LRYGB/LI	40.1	43.4	-	-	ND	ND	-	-	ND	ND	-	-	ND	ND	-	-
Panosian J [66]	LRYGB/LI	36	36.5	-	-	ND	ND	-	-	ND	ND	-	-	ND	ND	-	-
Accardi R [67]	LRYGB/LAGB	45	41	-	-	ND	ND	-	-	ND	ND	-	-	ND	ND	-	-
Elrefai M [68]	LRYGB/LSG/BPD-DS./LAGB	46.3	56.1	56.1	46.3	ND	ND	ND	ND	ND	ND	ND	ND	ND	ND	ND	ND
Biter LU [69]	LRYGB/LSG	44.03	44.17	-	-	20.27%	25%	-	-	12.16%	6.58%	-	-	17.57%	25%	-	-
Peterli R [70]	LSG/LRYGB	43.4	44	-	-	24.30%	26.36%	-	-	47.66%	41.82%	-	-	62.62%	59.09%	-	-

Svanevik M [71]	LRYGB/distal-GB	53.3	53.6	-	-	ND	ND	-	-	ND	ND	-	-	ND	ND	-	-
Versteegden DPA [72]	LSG/LRYGB	44.5	42.3	-	-	17.57%	17.76%	-	-	16.07%	10.81%	-	-	39.34%	34.17%	-	-
Schauer PR [73]	LI/LRYGB/LSG	36.4	37.1	36.1	-	97.50%	97.92%	97.96%	-	ND	ND	ND	-	ND	ND	ND	-
Salminen P [24]	LSG/LRYGB	47.3	48.4	-	-	42.98%	41.18%	-	-	ND	ND	-	-	68.6%	73.11%	-	-
Homan J [31]	LRYGB/LB-GB	45	43	-	-	31.08%	34.33%	-	-	ND	ND	-	-	32.43%	49.25%	-	-
Peterli R [25]	LSG/LRYGB	43.6	44.2	-	-	24.3%	26.36%	-	-	47.66%	41.82%	-	-	62.62%	59.09%	-	-
Elias K [74]	LRYGB/BPD-DS.	42.7	57.1	-	-	ND	ND	-	-	ND	ND	-	-	ND	ND	-	-
Silva JN [75]	LRYGB/LSG	41.41	43.73	-	-	ND	ND	-	-	ND	ND	-	-	ND	ND	-	-
Catheline JM [76]	LSG/LRYGB	45.2	45.3	-	-	ND	ND	-	-	ND	ND	-	-	ND	ND	-	-
Nabil TM [39]	OAGB/distal-OAGB	52.2	54.9	-	-	46.67%	53.33%	-	-	ND	ND	-	-	33.33%	30%	-	-
Skogar M [77]	BPD-DS./LRYGB	55	54.7	-	-	20.12%	14.49%	-	-	ND	ND	-	-	38.14%	35.96%	-	-
Lechaux D [78]	LSG/OAGB	45.8	45.3	-	-	ND	ND	-	-	ND	ND	-	-	ND	ND	-	-
Monpellier VM [79]	LSG/LRYGB	48.1	44.6	-	-	12.33%	24.43%	-	-	15.07%	11.09%	-	-	34.7%	39.41%	-	-
Poelemeijer	LSG/LRYGB	45.8	43	-	-	17.62%	29.75%	-	-	20.73%	20.83%	-	-	28.91%	40.86%	-	-

YQM [80]																	

LI – lifestyle intervention

LSG - laparoscopic sleeve gastrectomy

LRYGB - laparoscopic Roux-en-Y gastric bypass

BPD-DS - laparoscopic biliopancreatic diversion with duodenal switch

VBG - vertical banded gastroplasty

LAGB - laparoscopic adjustable gastric banding

LGCP - laparoscopic greater curvature plication

OAGB – one anastomosis gastric bypass

Banded-GB – banded laparoscopic Roux-en-Y gastric bypass

Distal-GB – distal laparoscopic Roux-en-Y gastric bypass

Distal-OAGB – distal one anastomosis gastric bypass

LB-GB – prolonged biliopancreatic limb gastric bypass

BMI – body mass index

DM – diabetes mellitus

OSAS – obstructive sleep apnea syndrome

HT – hypertension

ND- no data

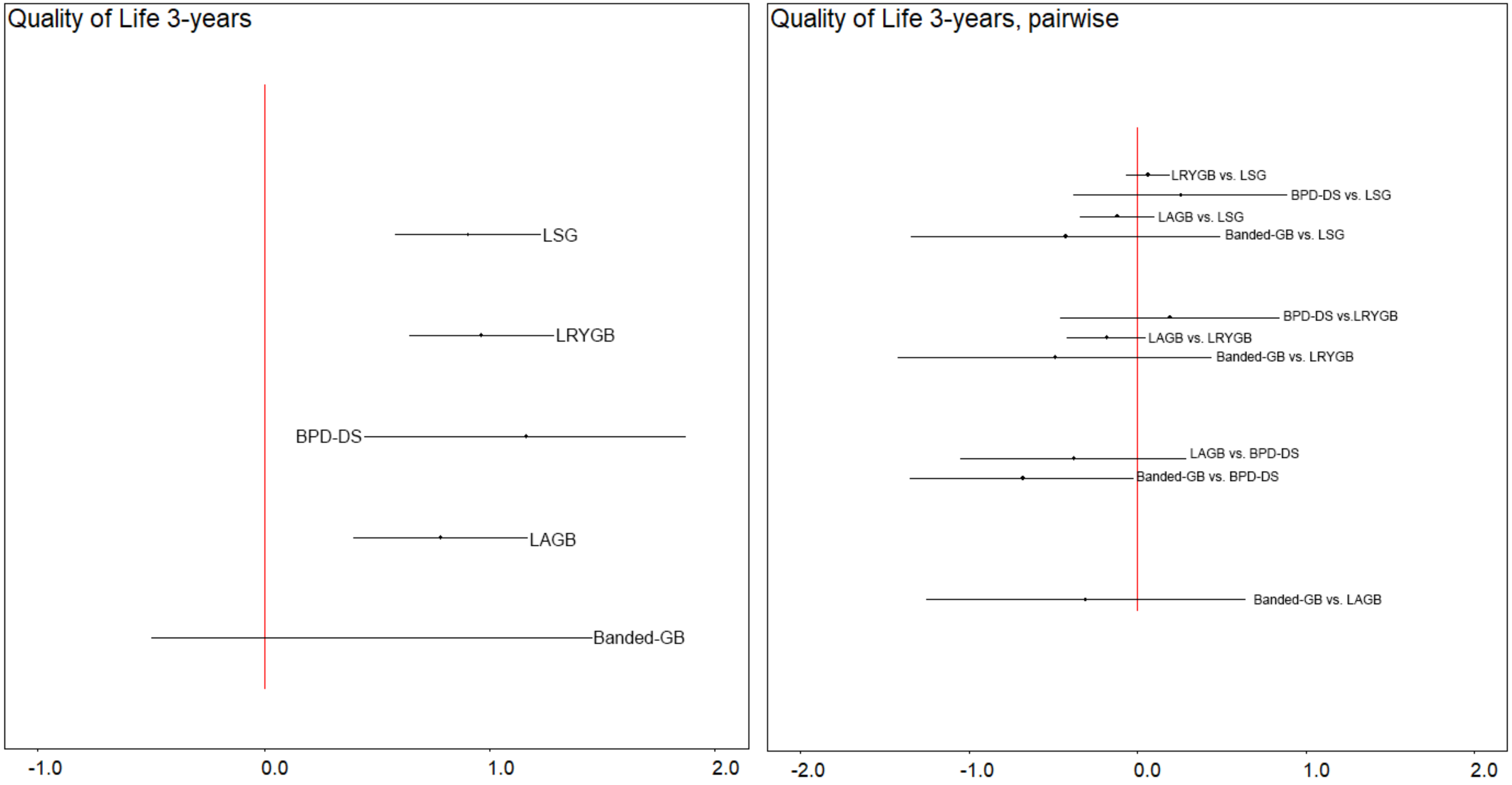

Pooled results of total HRQoL presented as SMD at 3 years a) in comparison to lifestyle intervention b)pairwise comparisons between surgeries

HRQoL after 3 years presented GIQLI scale (0-144)						
	**LI**	**LSG**	**LRYGB**	**BPD-DS**	**LAGB**	**Banded-GB**
**LSG**	20.05(12.89 to 27.29)	-	-1.35(-4.26 to 1.55)	-5.72(-19.83 to 8.44)	2.68(-2.23 to 7.58)	9.42(-11.05 to 29.89)
**LRYGB**	21.4(14.37 to 28.51)	1.35(-1.55 to 4.26)	-	-4.37(-18.78 to 10.08)	4.03(-1.17 to 9.24)	10.77(-9.9 to 31.44)
**BPD-DS**	25.76(9.88 to 41.58)	5.72(-8.44 to 19.83)	4.37(-10.08 to 18.78)	-	8.39(-6.54 to 23.34)	15.14(0.35 to 29.95)
**LAGB**	17.38(8.87 to 25.92)	-2.68(-7.58 to 2.23)	-4.03(-9.24 to 1.17)	-8.39(-23.34 to 6.54)	-	6.75(-14.28 to 27.74)
**Banded-GB**	10.63(-11.08 to 32.28)	-9.42(-29.89 to 11.05)	-10.77(-31.44 to 9.9)	-15.14(-29.95 to -0.35)	-6.75(-27.74 to 14.28)	**-**
MD >0 favors intervention in row; MD<0 favors intervention in column<br>MCID >5 marked green						

HRQoL – health-related quality of life
LI – lifestyle intervention
LSG – laparoscopic sleeve gastrectomy
LRYGB – laparoscopic Roux-en-Y gastric bypass
LAGB - laparoscopic adjustable gastric banding
Banded-GB – banded Roux-en-Y gastric bypass
BPD-DS – laparoscopic biliopancreatic diversion with duodenal switch

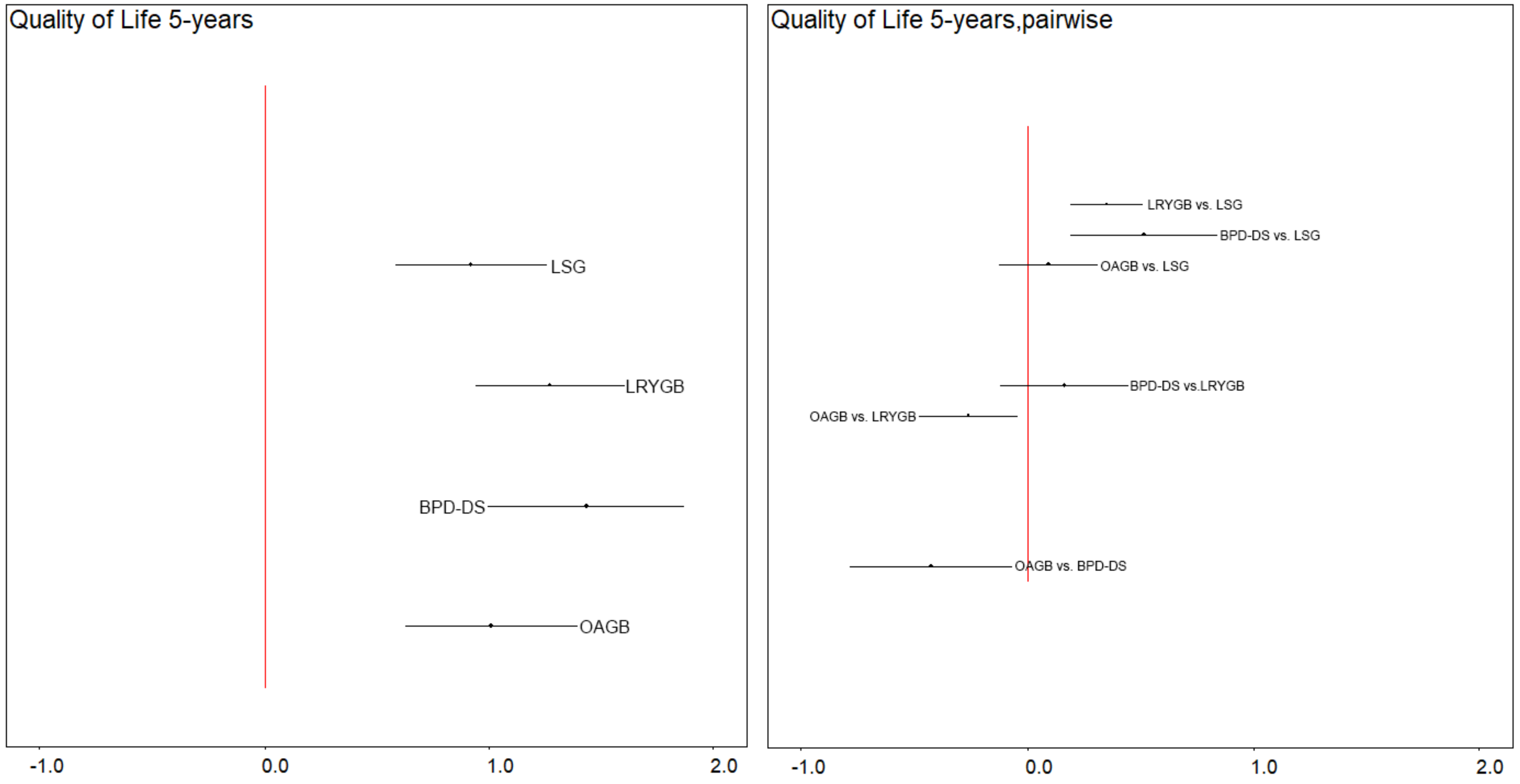

Pooled results of total HRQoL presented as SMD at 5 years a) in comparison to lifestyle intervention b)pairwise comparisons between surgeries

HRQoL after 5 years presented GIQLI scale (0-144)					
	**LI**	**LSG**	**LRYGB**	**BPD-DS**	**OAGB**
**LSG**	11.83(7.53 to 16.18)	-	-4.54(-6.61 to -2.46)	-6.66(-10.86 to -2.46)	-1.19(-4 to 1.63)
**LRYGB**	16.36(12.08 to 20.69)	4.54(2.46 to 6.61)	-	-2.12(-5.78 to 1.55)	3.35(0.53 to 6.17)
**BPD-DS**	17.49(12.85 to 24.15)	6.66(2.46 to 10.86)	2.12(-1.55 to 5.78)	-	5.47(0.86 to 10.1)
**OAGB**	13.01(8.11 to 17.98)	1.19(-1.63 to 4)	-3.35(-6.17 to -0.53)	-5.47(-10.1 to -0.86)	-

MD >0 favors intervention in row; MD<0 favors intervention in column
MCID >5 marked green

HRQoL – health-related quality of life
LI – lifestyle intervention
LSG – laparoscopic sleeve gastrectomy
BPD-DS – laparoscopic biliopancreatic diversion with duodenal switch
OAGB – one anastomosis gastric bypass
LRYGB – laparoscopic Roux-en-Y gastric bypass
