# Supplementary material for: Quality of Life After Bariatric Surgery—a Systematic Review with Bayesian Network Meta-analysis
Source: Obes Surg. 2021 Oct 11;31(12):5213–23. doi: 10.1007/s11695-021-05687-1 (PMC8595157; doi:10.1007/s11695-021-05687-1)
Supplement: Supplementary file 4 — Supplementary file4 (PDF 1468 KB) [file 11695_2021_5687_MOESM4_ESM.pdf]

Table of contents

Total QoL 1 year 2
QoL 1-year physical 5
Qol 1-year emotional 8
QoL 1-year general health 11
QoL 1-year vitality 13
QoL 1-year physical role 15
QoL 1-year social 17
QoL 1-year mental 20
QoL 1-year bodily pain 22
Total QoL 2-years 24
QoL 2-years physical 27
Qol 2-years emotional 30
QoL 2-years general health 32
QoL 2-years vitality 34
QoL 2-years physical role 36
QoL 2-years social 38
QoL 2-years mental 40
QoL 2-years bodily pain 42
Total QoL 3-years 44
Total QoL 5-years 47

Total QoL 1 year

22 studies

Legend:

1 LI
2 LSG
3 LRYGB
4 BPD-DS
5 VBG
6 LAGB
7 LGCP
8 OAGB
9 Distal OAGB

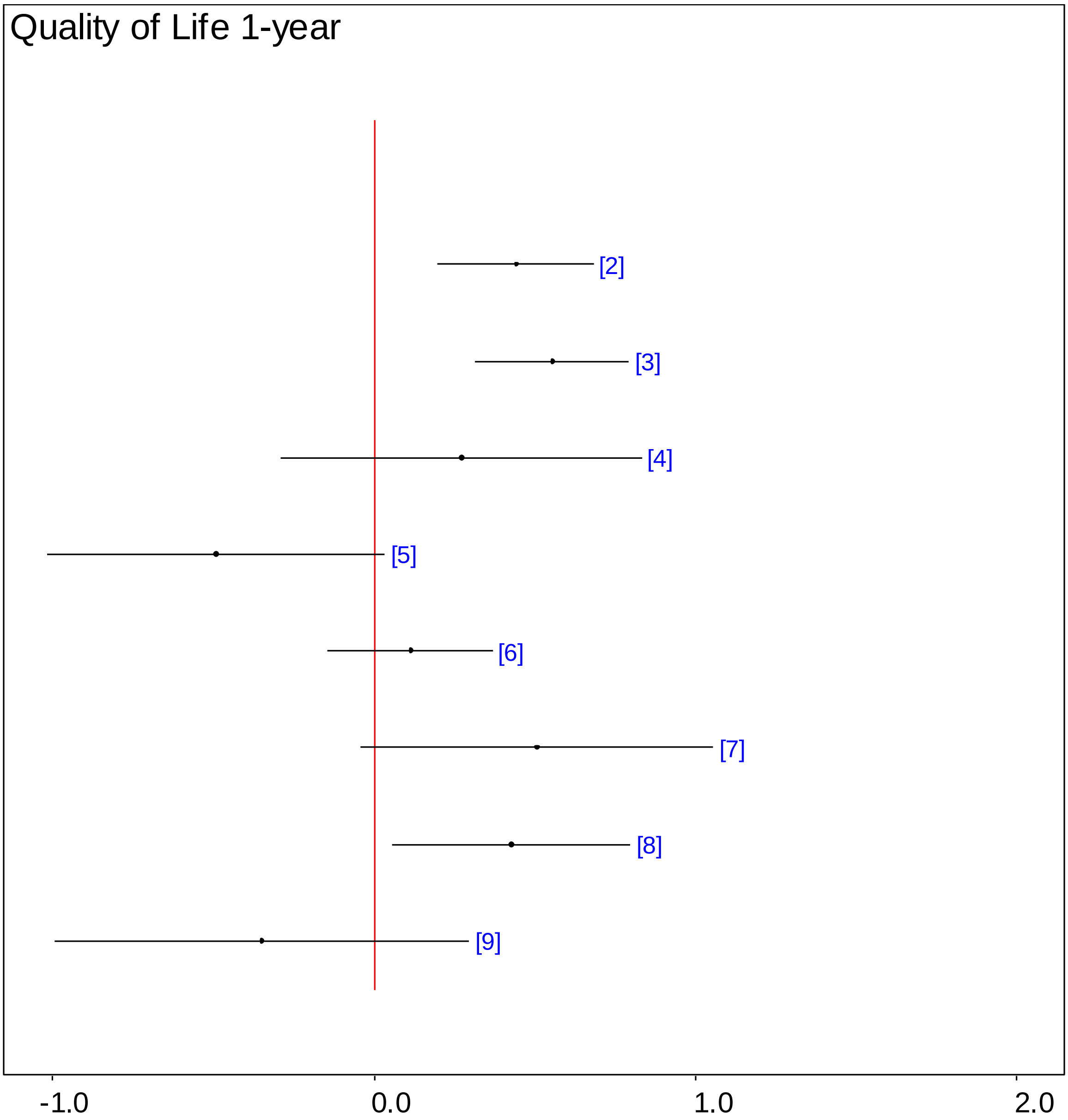

node	mean	sd	MC error	2.5%	median	97.5%

d[2]	0.4443	0.1237	0.001649	0.2002	0.4451	0.684
d[3]	0.5566	0.123	0.001635	0.3141	0.5572	0.7955
d[4]	0.2723	0.2874	0.001708	-0.2912	0.2728	0.8336
d[5]	-0.4902	0.269	0.001711	-1.017	-0.4901	0.03589
d[6]	0.116	0.1306	0.001673	-0.1418	0.1165	0.3696
d[7]	0.5075	0.2802	0.001734	-0.04188	0.5073	1.055
d[8]	0.4275	0.189	0.001699	0.05596	0.4276	0.7978
d[9]	-0.3478	0.3286	0.001816	-0.9926	-0.3478	0.2959
totresdev	23.86	7.025	0.01287	12.14	23.18	39.5

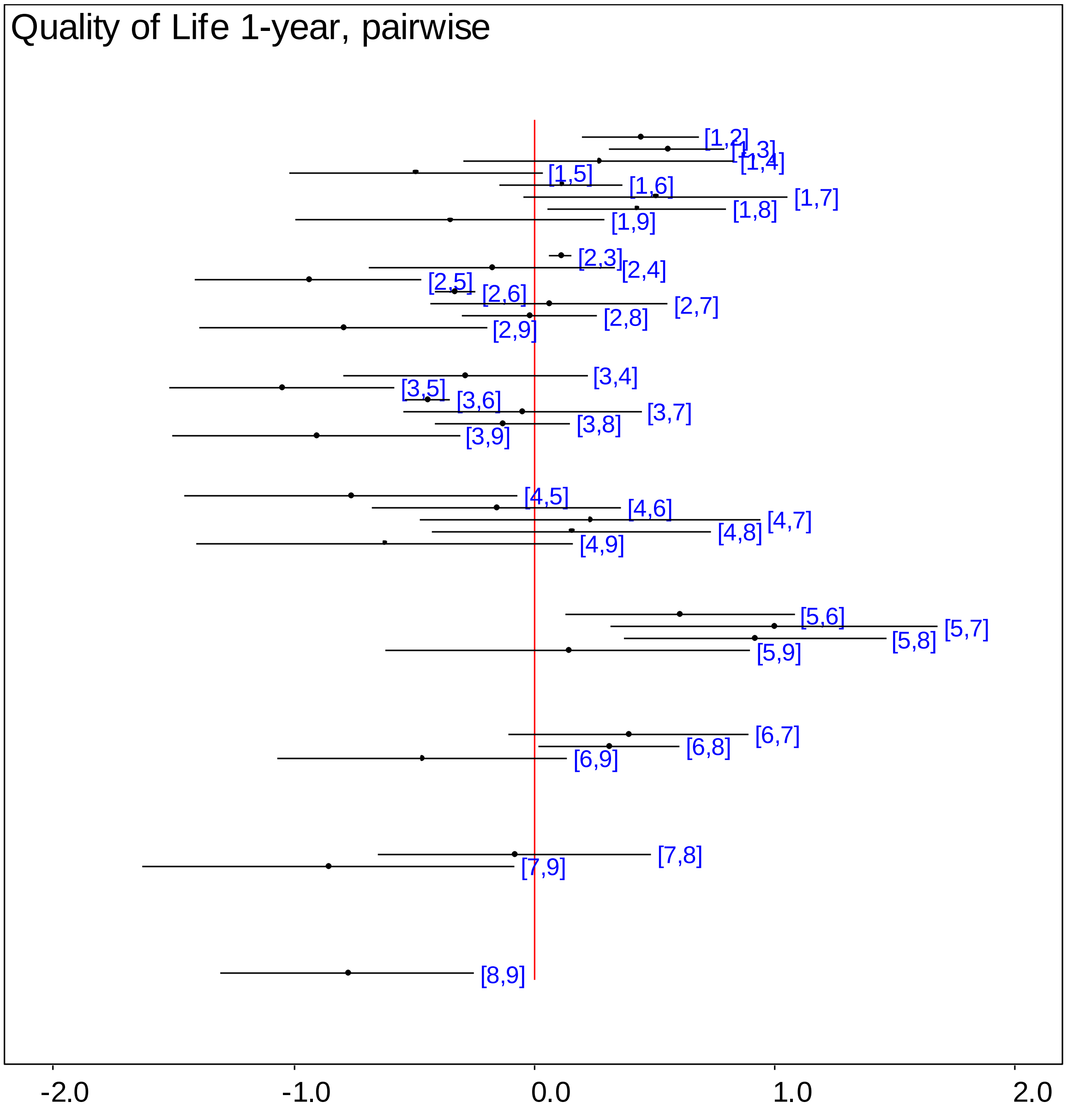

node	mean	sd	MC error	2.5%	median	97.5%
diff[1,2]	0.4443	0.1237	0.001649	0.2002	0.4451	0.684
diff[1,3]	0.5566	0.123	0.001635	0.3141	0.5572	0.7955

diff[1,4]	0.2723	0.2874	0.001708	-0.2912	0.2728	0.8336
diff[1,5]	-0.4902	0.269	0.001711	-1.017	-0.4901	0.03589
diff[1,6]	0.116	0.1306	0.001673	-0.1418	0.1165	0.3696
diff[1,7]	0.5075	0.2802	0.001734	-0.04188	0.5073	1.055
diff[1,8]	0.4275	0.189	0.001699	0.05596	0.4276	0.7978
diff[1,9]	-0.3478	0.3286	0.001816	-0.9926	-0.3478	0.2959
diff[2,3]	0.1123	0.02353	3.436E-5	0.06607	0.1122	0.1584
diff[2,4]	-0.172	0.2609	4.35E-4	-0.6845	-0.1716	0.3371
diff[2,5]	-0.9345	0.2401	4.065E-4	-1.407	-0.9341	-0.4653
diff[2,6]	-0.3284	0.04306	7.293E-5	-0.4126	-0.3283	-0.244
diff[2,7]	0.06321	0.2513	4.307E-4	-0.4296	0.06307	0.5578
diff[2,8]	-0.01682	0.1434	2.979E-4	-0.2984	-0.01674	0.2643
diff[2,9]	-0.7921	0.3044	6.255E-4	-1.39	-0.7921	-0.1959
diff[3,4]	-0.2843	0.2598	4.33E-4	-0.7944	-0.2837	0.2228
diff[3,5]	-1.047	0.2389	4.035E-4	-1.517	-1.047	-0.5797
diff[3,6]	-0.4406	0.04739	8.713E-5	-0.5333	-0.4407	-0.3478
diff[3,7]	-0.04907	0.2523	4.333E-4	-0.5435	-0.04921	0.4469
diff[3,8]	-0.1291	0.1438	3.007E-4	-0.4116	-0.1291	0.1528
diff[3,9]	-0.9044	0.3046	6.291E-4	-1.503	-0.9043	-0.3073
diff[4,5]	-0.7625	0.3534	5.999E-4	-1.454	-0.763	-0.06941
diff[4,6]	-0.1563	0.2641	4.455E-4	-0.6724	-0.157	0.3614
diff[4,7]	0.2352	0.3618	5.998E-4	-0.4726	0.2357	0.9451
diff[4,8]	0.1552	0.2968	5.276E-4	-0.4249	0.1549	0.7377
diff[4,9]	-0.6201	0.4002	7.805E-4	-1.406	-0.6201	0.1654
diff[5,6]	0.6062	0.2436	4.119E-4	0.1296	0.6062	1.084
diff[5,7]	0.9977	0.3474	5.961E-4	0.3167	0.9977	1.679
diff[5,8]	0.9177	0.2788	4.93E-4	0.3734	0.9172	1.465
diff[5,9]	0.1424	0.3875	7.366E-4	-0.6163	0.1423	0.9019
diff[6,7]	0.3916	0.255	4.417E-4	-0.108	0.3914	0.8933
diff[6,8]	0.3115	0.1495	3.079E-4	0.01743	0.3114	0.6043
diff[6,9]	-0.4638	0.3073	6.315E-4	-1.067	-0.4641	0.1379
diff[7,8]	-0.08002	0.2889	5.235E-4	-0.6474	-0.08071	0.4864
diff[7,9]	-0.8553	0.3946	7.569E-4	-1.627	-0.8551	-0.07992
diff[8,9]	-0.7753	0.2685	4.386E-4	-1.304	-0.7748	-0.2474

QoL 1-year physical

10 studies

Legend:

1 LI
2 LSG
3 LRYGB
4 BPD-DS
5 LAGB
6 LGCP
7 OAGB

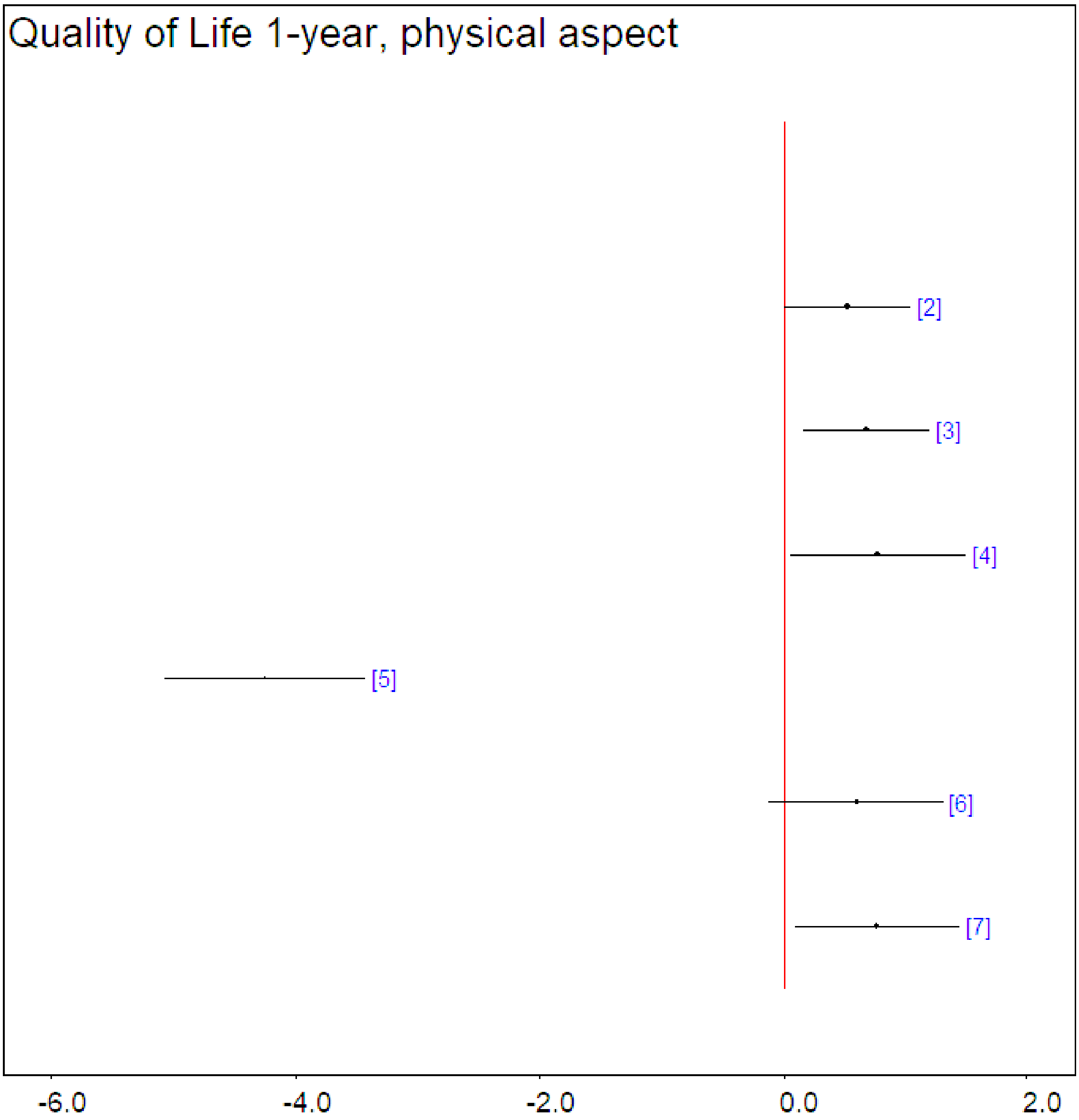

node	mean	sd	MC error	2.5%	median	97.5%
d[2]	0.5272	0.2654	0.001323	0.007936	0.5266	1.047
d[3]	0.6814	0.2636	0.001315	0.1662	0.6811	1.198
d[4]	0.7755	0.3685	0.001506	0.05266	0.7761	1.497
d[5]	-4.247	0.4174	0.001561	-5.067	-4.247	-3.428
d[6]	0.601	0.3658	0.00151	-0.1171	0.6011	1.317
d[7]	0.77	0.3459	0.001479	0.09315	0.7698	1.447

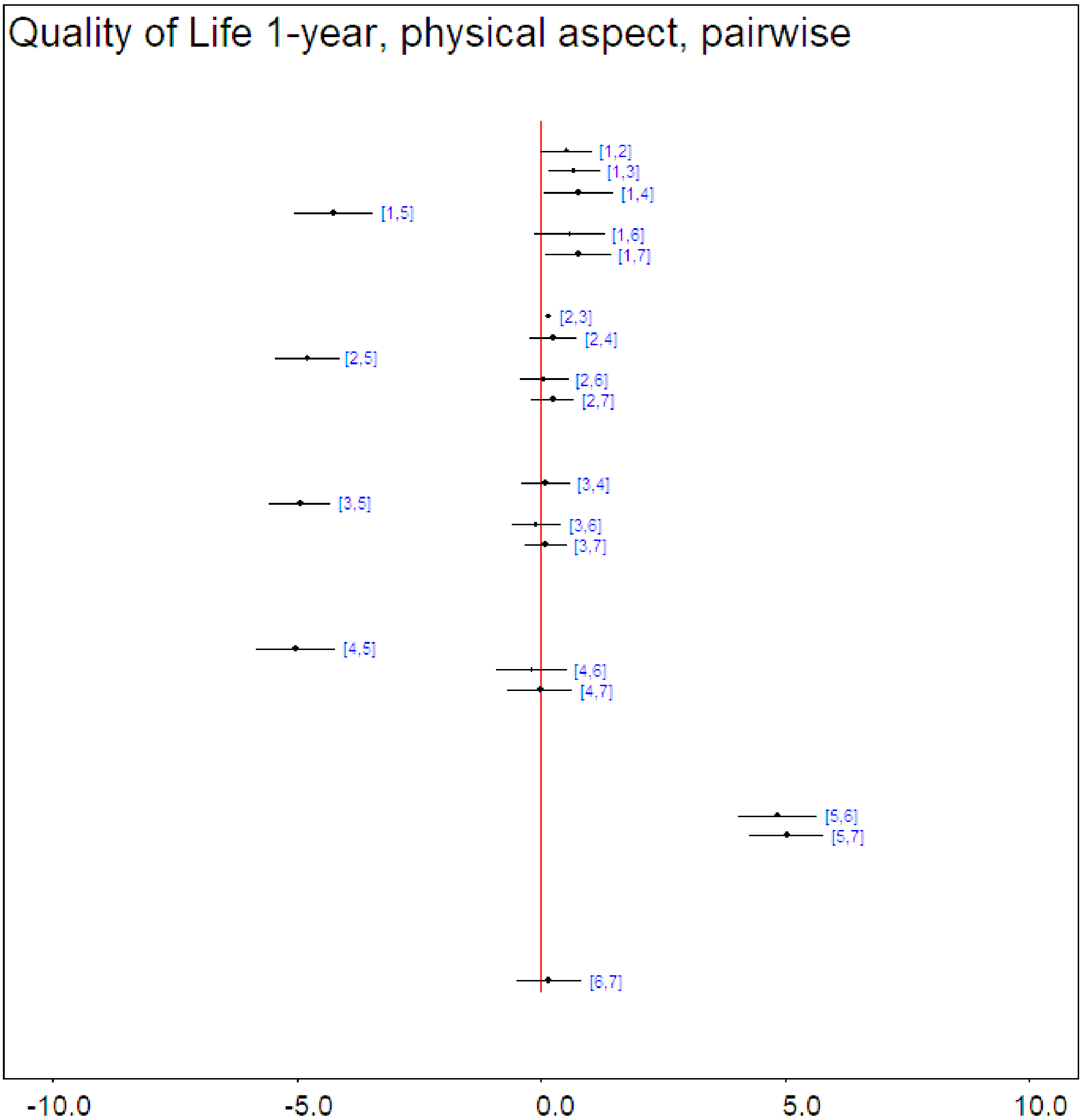

node	mean	sd	MC error	2.5%	median	97.5%
diff[1,2]	0.5272	0.2654	0.001323	0.007936	0.5266	1.047
diff[1,3]	0.6814	0.2636	0.001315	0.1662	0.6811	1.198
diff[1,4]	0.7755	0.3685	0.001506	0.05266	0.7761	1.497
diff[1,5]	-4.247	0.4174	0.001561	-5.067	-4.247	-3.428
diff[1,6]	0.601	0.3658	0.00151	-0.1171	0.6011	1.317
diff[1,7]	0.77	0.3459	0.001479	0.09315	0.7698	1.447
diff[2,3]	0.1542	0.03014	5.166E-5	0.09524	0.1541	0.2133
diff[2,4]	0.2483	0.2599	4.454E-4	-0.2605	0.2484	0.7598
diff[2,5]	-4.774	0.3251	5.393E-4	-5.413	-4.774	-4.137
diff[2,6]	0.07379	0.2517	4.109E-4	-0.4221	0.0743	0.5687
diff[2,7]	0.2428	0.2256	3.712E-4	-0.1996	0.2431	0.6847
diff[3,4]	0.09415	0.2582	4.421E-4	-0.4122	0.09401	0.6024
diff[3,5]	-4.928	0.3237	5.39E-4	-5.564	-4.928	-4.294
diff[3,6]	-0.08037	0.2534	4.206E-4	-0.5786	-0.08011	0.4177
diff[3,7]	0.08859	0.2237	3.702E-4	-0.3496	0.08874	0.5265

diff[4,5]	-5.022	0.4136	6.969E-4	-5.834	-5.022	-4.211
diff[4,6]	-0.1745	0.3621	6.067E-4	-0.8835	-0.1743	0.5376
diff[4,7]	-0.005558	0.3411	5.81E-4	-0.6737	-0.005835	0.6625
diff[5,6]	4.848	0.4111	6.77E-4	4.043	4.847	5.655
diff[5,7]	5.017	0.3935	6.483E-4	4.245	5.017	5.788
diff[6,7]	0.169	0.3385	5.609E-4	-0.4964	0.1693	0.8325

Qol 1-year emotional

9 studies

Legend:

1	LI
2	LSG
3	LRYGB
4	BPD-DS
5	LAGB
6	LGCP
7	OAGB

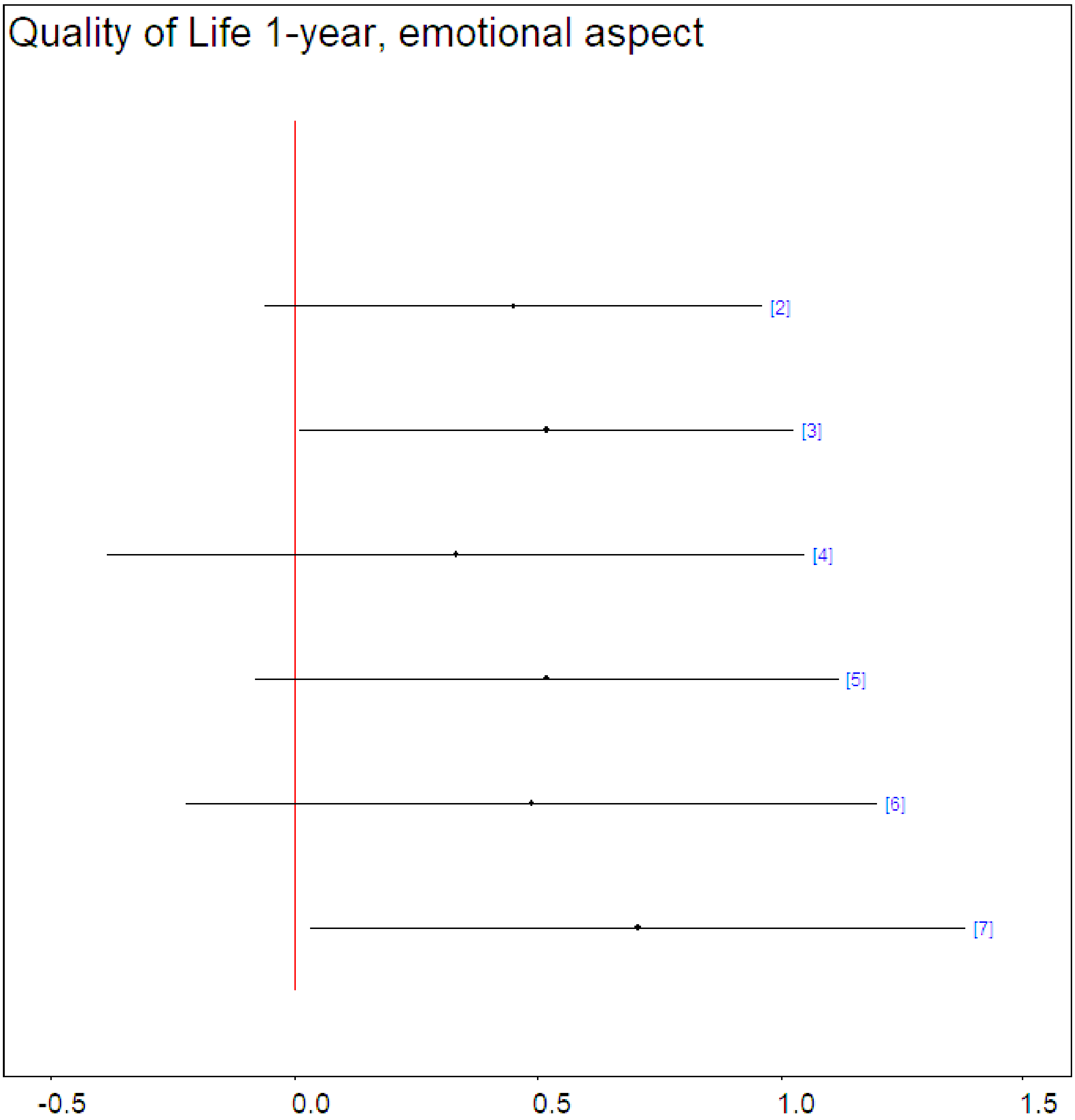

node	mean	sd	MC error	2.5%	median	97.5%
d[2]	0.4499	0.2624	0.001548	-0.06173	0.4498	0.9645
d[3]	0.519	0.2606	0.001541	0.009829	0.5188	1.03

d[4]	0.3326	0.3667	0.001701	-0.3853	0.3325	1.052
d[5]	0.5186	0.3073	0.001684	-0.08157	0.5181	1.121
d[6]	0.4884	0.3636	0.001708	-0.2248	0.4882	1.201
d[7]	0.7056	0.3439	0.001679	0.03247	0.7055	1.381

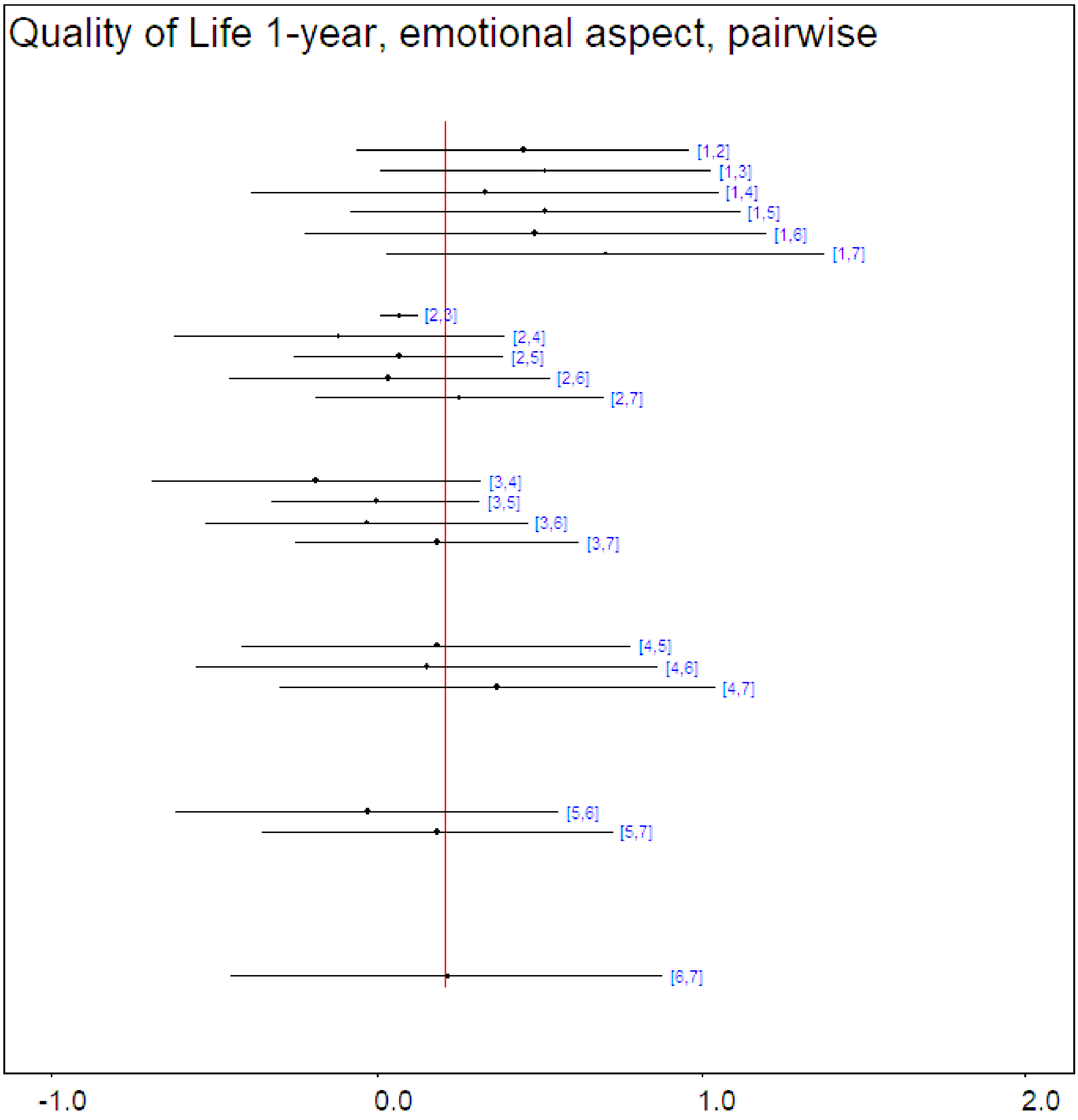

node	mean	sd	MC error	2.5%	median	97.5%
diff[1,2]	0.4499	0.2624	0.001548	-0.06173	0.4498	0.9645
diff[1,3]	0.519	0.2606	0.001541	0.009829	0.5188	1.03
diff[1,4]	0.3326	0.3667	0.001701	-0.3853	0.3325	1.052
diff[1,5]	0.5186	0.3073	0.001684	-0.08157	0.5181	1.121
diff[1,6]	0.4884	0.3636	0.001708	-0.2248	0.4882	1.201
diff[1,7]	0.7056	0.3439	0.001679	0.03247	0.7055	1.381
diff[2,3]	0.06908	0.0303	5.194E-5	0.009818	0.06904	0.1286
diff[2,4]	-0.1173	0.2604	4.462E-4	-0.6271	-0.1173	0.395
diff[2,5]	0.06871	0.1656	2.739E-4	-0.2567	0.06883	0.393
diff[2,6]	0.03852	0.2517	4.108E-4	-0.4572	0.03902	0.5333

diff[2,7]	0.2557	0.226	3.718E-4	-0.1875	0.256	0.6985
diff[3,4]	-0.1864	0.2586	4.429E-4	-0.6936	-0.1866	0.3227
diff[3,5]	-3.616E-4	0.1628	2.711E-4	-0.32	-1.04E-4	0.3185
diff[3,6]	-0.03056	0.2534	4.206E-4	-0.5286	-0.03028	0.4673
diff[3,7]	0.1866	0.2241	3.709E-4	-0.2524	0.1867	0.6253
diff[4,5]	0.1861	0.3053	5.191E-4	-0.4145	0.1856	0.7847
diff[4,6]	0.1559	0.3623	6.072E-4	-0.5537	0.156	0.8684
diff[4,7]	0.373	0.3416	5.82E-4	-0.2963	0.3727	1.042
diff[5,6]	-0.03019	0.3012	4.958E-4	-0.6218	-0.03035	0.5588
diff[5,7]	0.1869	0.2769	4.553E-4	-0.3557	0.1867	0.7294
diff[6,7]	0.2171	0.3387	5.613E-4	-0.4486	0.2175	0.8812

QoL 1-year general health

8 studies

Legend:

1	LI
2	LSG
3	LRYGB
4	BPD-DS
5	LAGB
6	OAGB

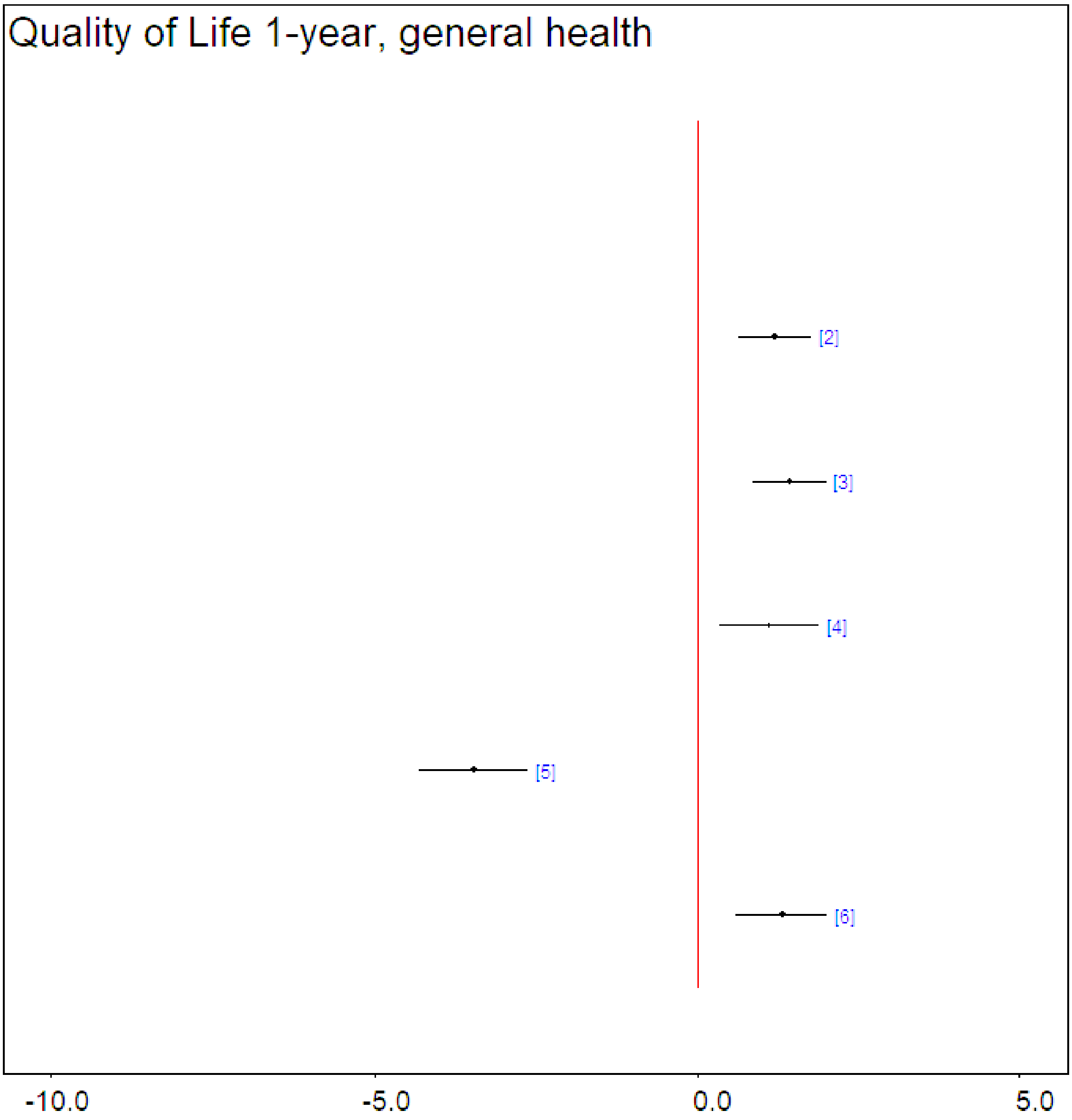

node	mean	sd	MC error	2.5%	median	97.5%
d[2]	1.199	0.2911	0.001457	0.6263	1.2	1.767
d[3]	1.436	0.2894	0.001459	0.866	1.437	2.0
d[4]	1.115	0.3891	0.001685	0.3491	1.117	1.875
d[5]	-3.452	0.4314	0.001738	-4.297	-3.451	-2.607
d[6]	1.311	0.3659	0.001656	0.5918	1.313	2.027

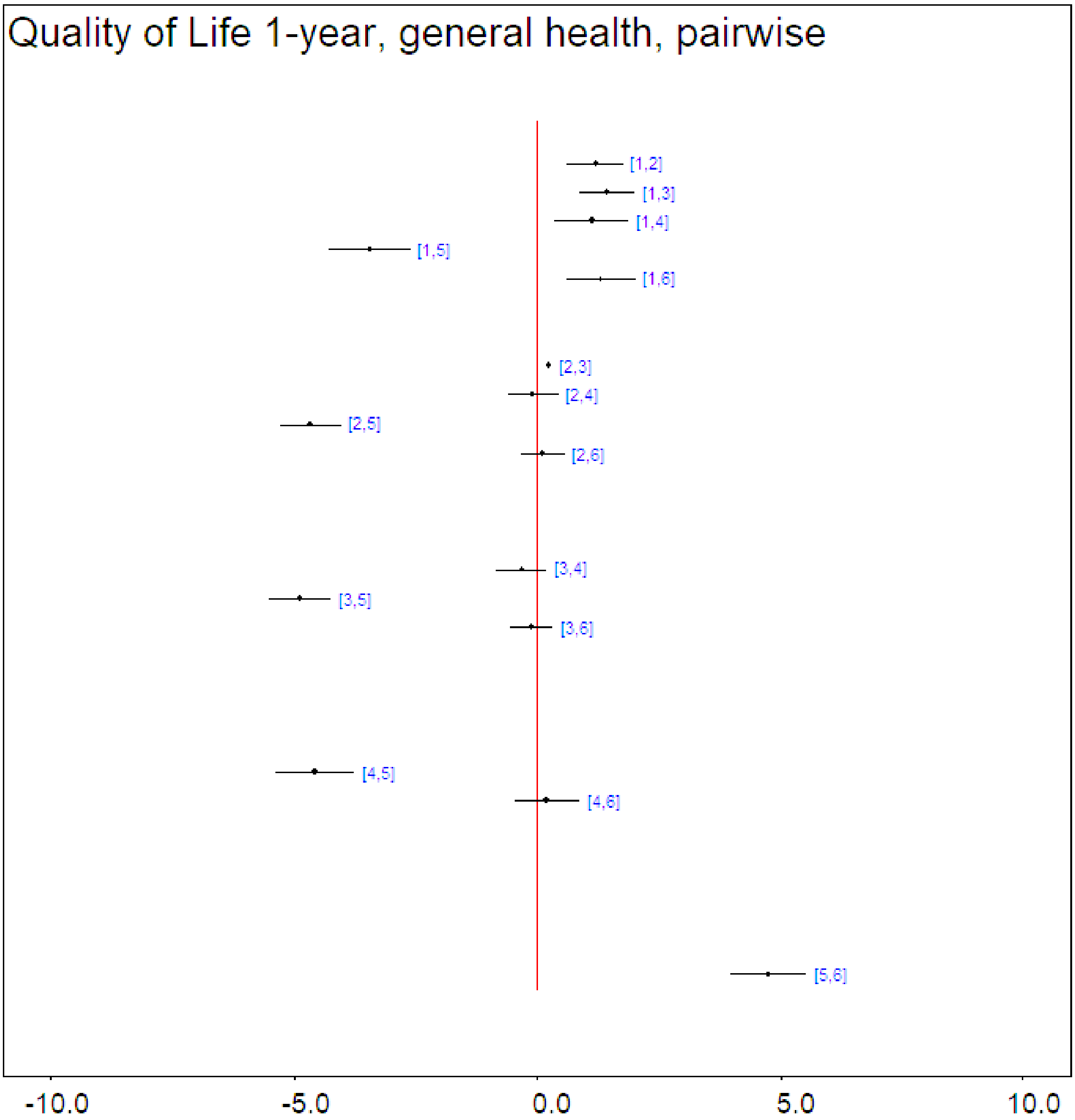

node	mean	sd	MC error	2.5%	median	97.5%
diff[1,2]	1.199	0.2911	0.001457	0.6263	1.2	1.767
diff[1,3]	1.436	0.2894	0.001459	0.866	1.437	2.0
diff[1,4]	1.115	0.3891	0.001685	0.3491	1.117	1.875
diff[1,5]	-3.452	0.4314	0.001738	-4.297	-3.451	-2.607
diff[1,6]	1.311	0.3659	0.001656	0.5918	1.313	2.027
diff[2,3]	0.2364	0.03042	5.083E-5	0.177	0.2364	0.2962
diff[2,4]	-0.08415	0.2616	4.368E-4	-0.598	-0.08363	0.4293
diff[2,5]	-4.651	0.3217	5.561E-4	-5.283	-4.651	-4.023
diff[2,6]	0.1119	0.2264	3.751E-4	-0.3315	0.1123	0.5554
diff[3,4]	-0.3205	0.2598	4.321E-4	-0.8313	-0.3201	0.1883
diff[3,5]	-4.888	0.3202	5.554E-4	-5.517	-4.887	-4.261
diff[3,6]	-0.1245	0.2243	3.701E-4	-0.5637	-0.1239	0.3153
diff[4,5]	-4.567	0.4127	6.923E-4	-5.377	-4.567	-3.76
diff[4,6]	0.1961	0.3435	5.588E-4	-0.4774	0.1961	0.87

diff[5,6]	4.763	0.3914	6.639E-4	3.998	4.763	5.534

QoL 1-year vitality

7 studies

Legend:

1 LI
2 LSG
3 LRYGB
4 BPD-DS
5 LAGB

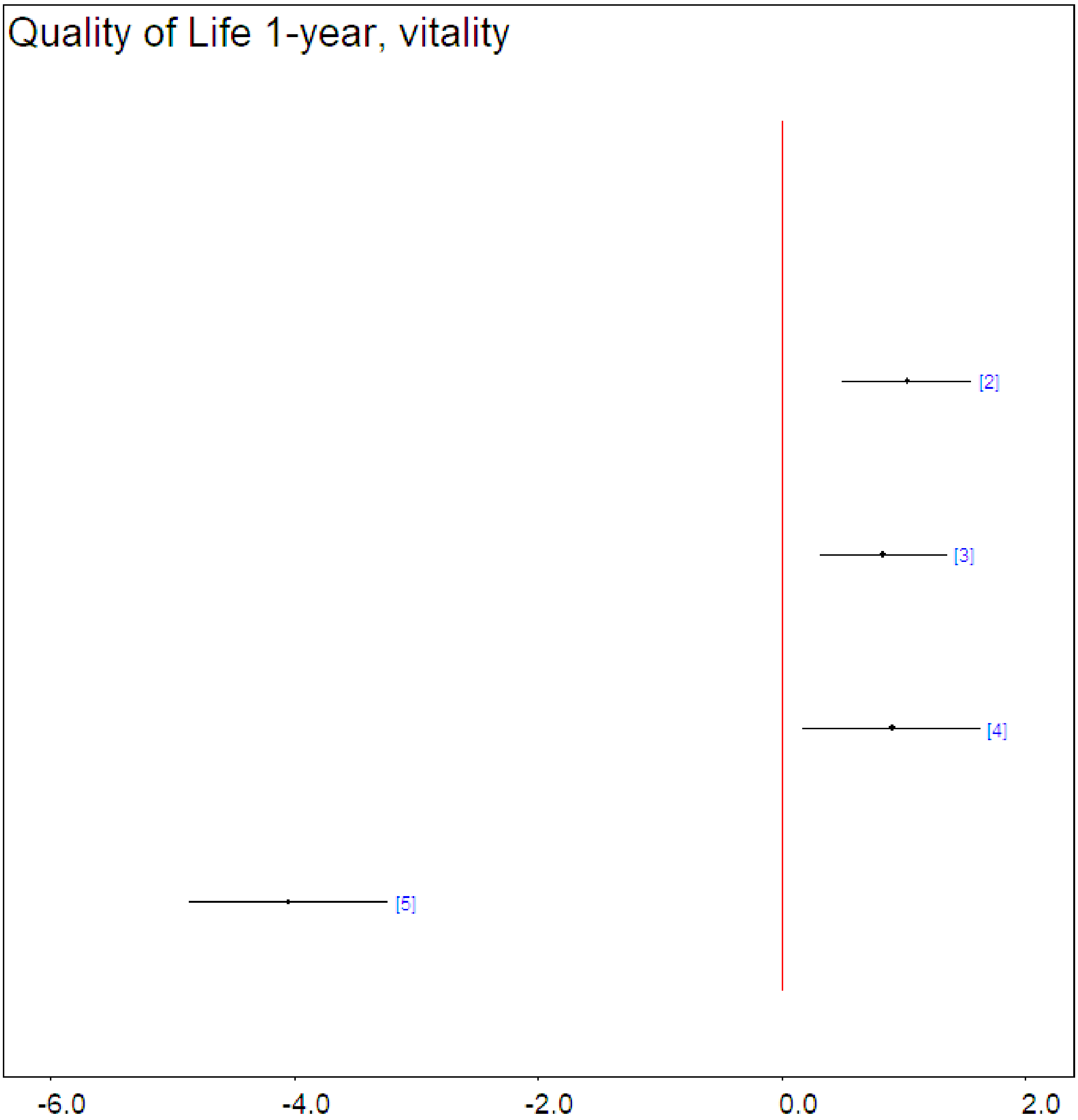

node	mean	sd	MC error	2.5%	median	97.5%
d[2]	1.023	0.2694	9.449E-4	0.4944	1.023	1.553
d[3]	0.8329	0.2676	9.416E-4	0.3079	0.8329	1.358

d[4]	0.8987	0.3715	0.001216	0.1711	0.8988	1.627
d[5]	-4.055	0.4185	0.001262	-4.875	-4.054	-3.237

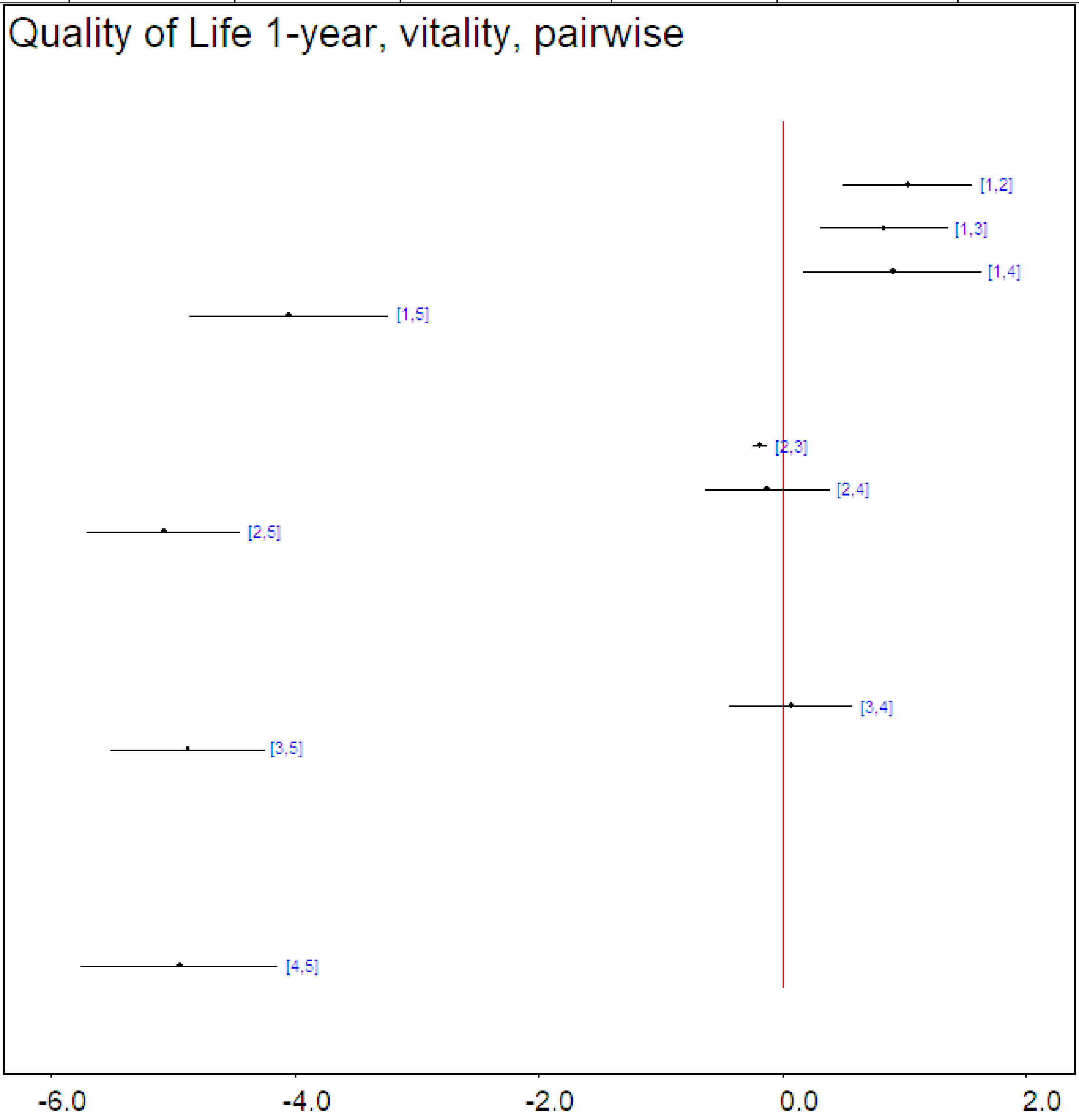

node	mean	sd	MC error	2.5%	median	97.5%
diff[1,2]	1.023	0.2694	9.449E-4	0.4944	1.023	1.553
diff[1,3]	0.8329	0.2676	9.416E-4	0.3079	0.8329	1.358
diff[1,4]	0.8987	0.3715	0.001216	0.1711	0.8988	1.627
diff[1,5]	-4.055	0.4185	0.001262	-4.875	-4.054	-3.237
diff[2,3]	-0.1903	0.03078	5.134E-5	-0.2508	-0.1903	-0.1301
diff[2,4]	-0.1245	0.2599	4.404E-4	-0.6337	-0.1242	0.3861
diff[2,5]	-5.078	0.3226	5.456E-4	-5.71	-5.078	-4.448
diff[3,4]	0.06583	0.2581	4.405E-4	-0.4407	0.06619	0.5725
diff[3,5]	-4.888	0.3211	5.445E-4	-5.518	-4.888	-4.26
diff[4,5]	-4.954	0.4124	7.081E-4	-5.763	-4.953	-4.143

QoL 1-year physical role

7 studies
Legend:
1 Lifestyle Intervention
2 LSG
3 LRYGB
4 Laparoscopic BPD-DS.

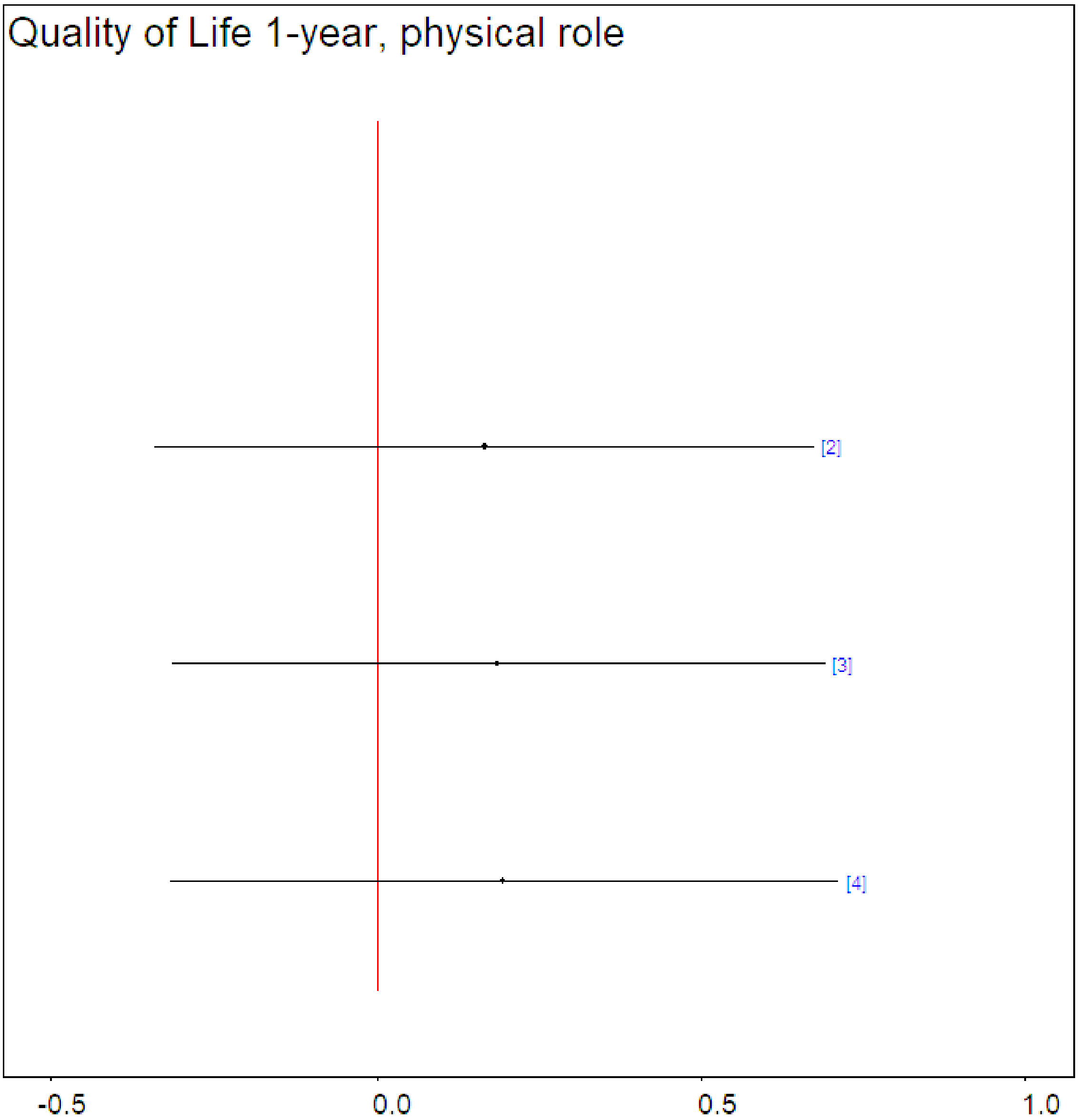

node	mean	sd	MC error	2.5%	median	97.5%
d[2]	0.166	0.2581	0.002594	-0.3395	0.165	0.6738
d[3]	0.1867	0.2558	0.002592	-0.3133	0.186	0.6908
d[4]	0.1953	0.2622	0.002657	-0.3175	0.1946	0.7116

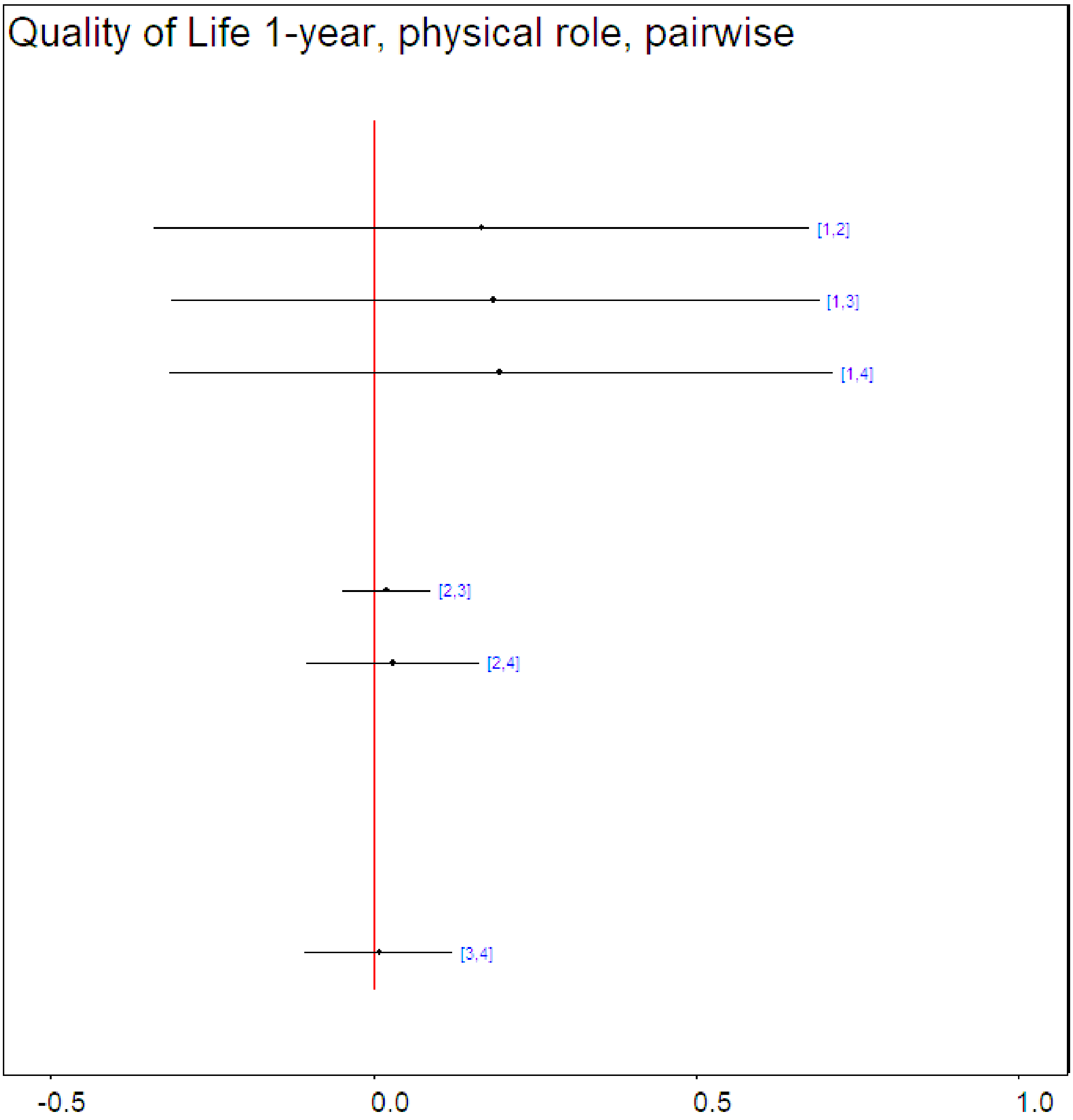

**node**	**mean**	**sd**	**MC error**	**2.5%**	**median**	**97.5%**
diff[1,2]	0.166	0.2581	0.002594	-0.3395	0.165	0.6738
diff[1,3]	0.1867	0.2558	0.002592	-0.3133	0.186	0.6908
diff[1,4]	0.1953	0.2622	0.002657	-0.3175	0.1946	0.7116
diff[2,3]	0.02076	0.03479	5.869E-5	-0.04733	0.02068	0.08897
diff[2,4]	0.0293	0.06815	1.134E-4	-0.1041	0.0294	0.1627
diff[3,4]	0.008535	0.05857	9.795E-5	-0.1062	0.008548	0.1233

QoL 1-year social

9 studies
Legend:
1 LI
2 LSG
3 LRYGB
4 BPD-DS
5 LGCP
6 OAGB

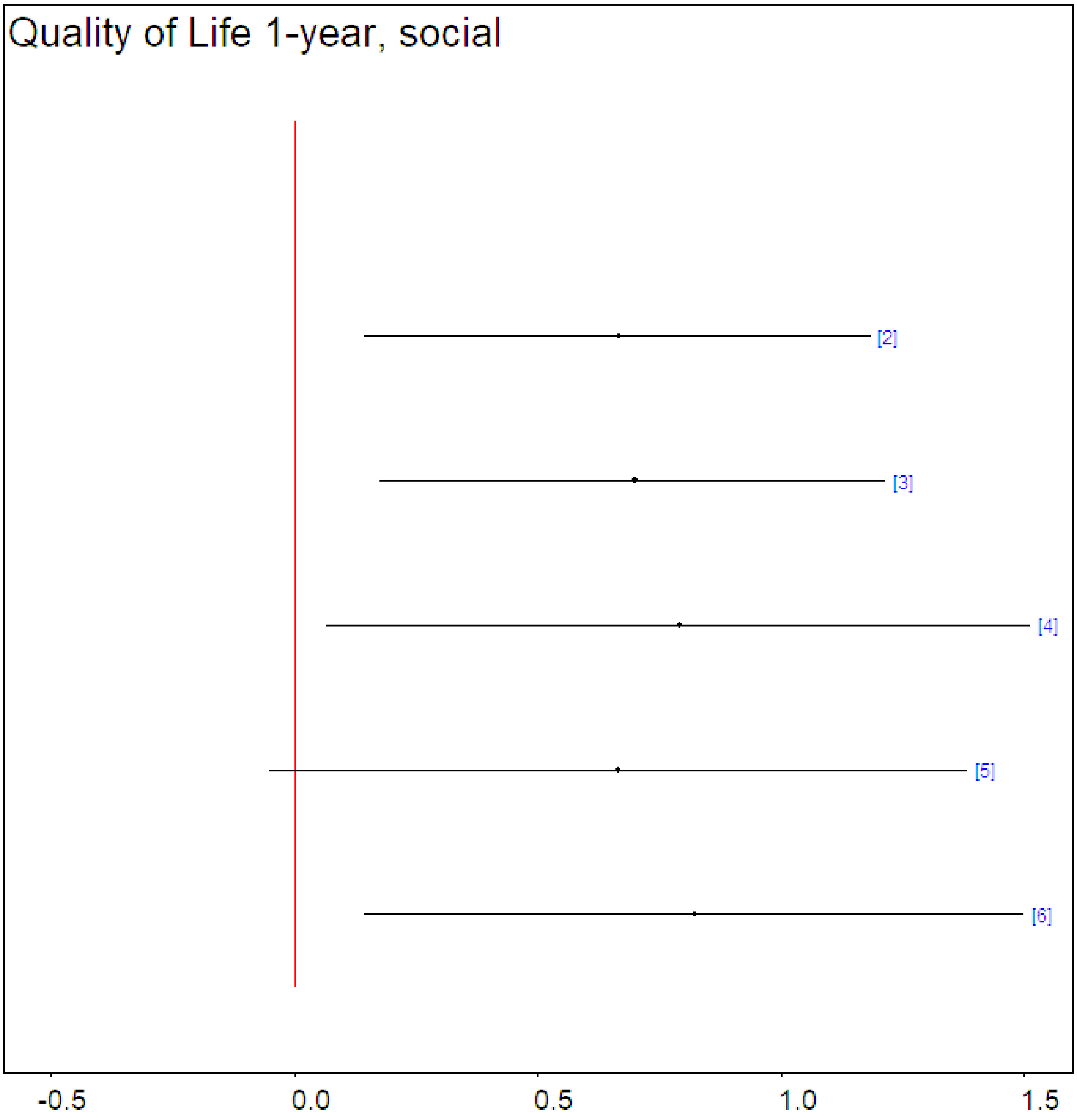

node	mean	sd	MC error	2.5%	median	97.5%
d[2]	0.6654	0.2662	0.001309	0.1415	0.6666	1.184
d[3]	0.6964	0.2645	0.001306	0.1758	0.6976	1.213
d[4]	0.7913	0.3697	0.001526	0.06337	0.7926	1.513
d[5]	0.6648	0.3658	0.001544	-0.05115	0.665	1.381

d[6]	0.8224	0.3465	0.001497	0.1419	0.824	1.5

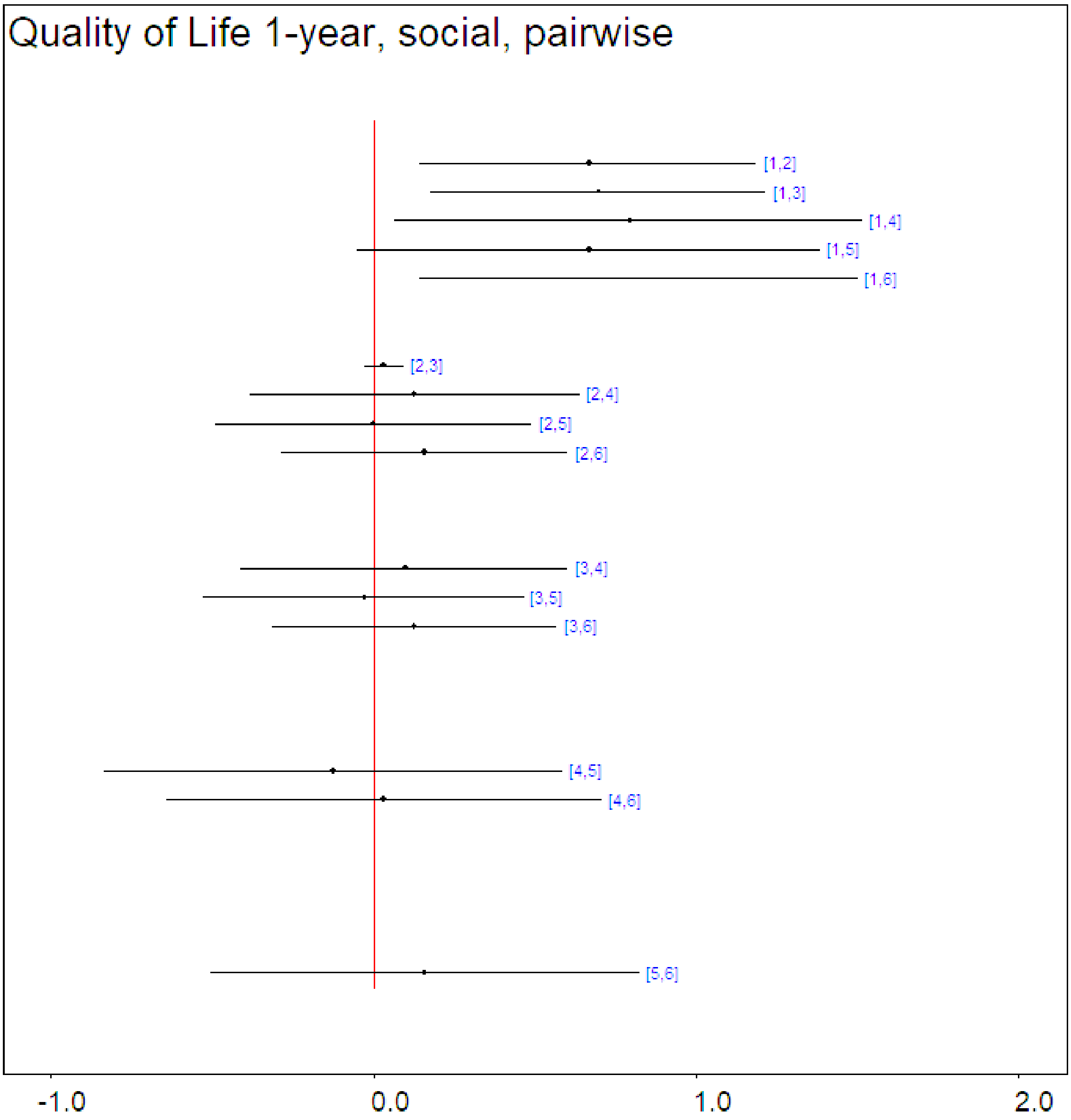

node	mean	sd	MC error	2.5%	median	97.5%
diff[1,2]	0.6654	0.2662	0.001309	0.1415	0.6666	1.184
diff[1,3]	0.6964	0.2645	0.001306	0.1758	0.6976	1.213
diff[1,4]	0.7913	0.3697	0.001526	0.06337	0.7926	1.513
diff[1,5]	0.6648	0.3658	0.001544	-0.05115	0.665	1.381
diff[1,6]	0.8224	0.3465	0.001497	0.1419	0.824	1.5
diff[2,3]	0.03099	0.03018	5.138E-5	-0.028	0.03092	0.09019
diff[2,4]	0.1259	0.26	4.34E-4	-0.3849	0.1264	0.6364
diff[2,5]	-5.959E-4	0.2512	4.356E-4	-0.4938	-2.579E-4	0.4907
diff[2,6]	0.157	0.2264	3.751E-4	-0.2868	0.1573	0.6004
diff[3,4]	0.09489	0.2582	4.295E-4	-0.4127	0.09531	0.6006
diff[3,5]	-0.03158	0.253	4.464E-4	-0.5289	-0.03144	0.4637

diff[3,6]	0.126	0.2243	3.701E-4	-0.3133	0.1266	0.5658
diff[4,5]	-0.1265	0.3618	6.104E-4	-0.8363	-0.1263	0.5834
diff[4,6]	0.03113	0.3423	5.568E-4	-0.64	0.03109	0.7025
diff[5,6]	0.1576	0.3385	5.776E-4	-0.5048	0.1572	0.8217

QoL 1-year mental

7 studies
Legend:
1 LI
2 LSG
3 LRYGB
4 BPD-DS.

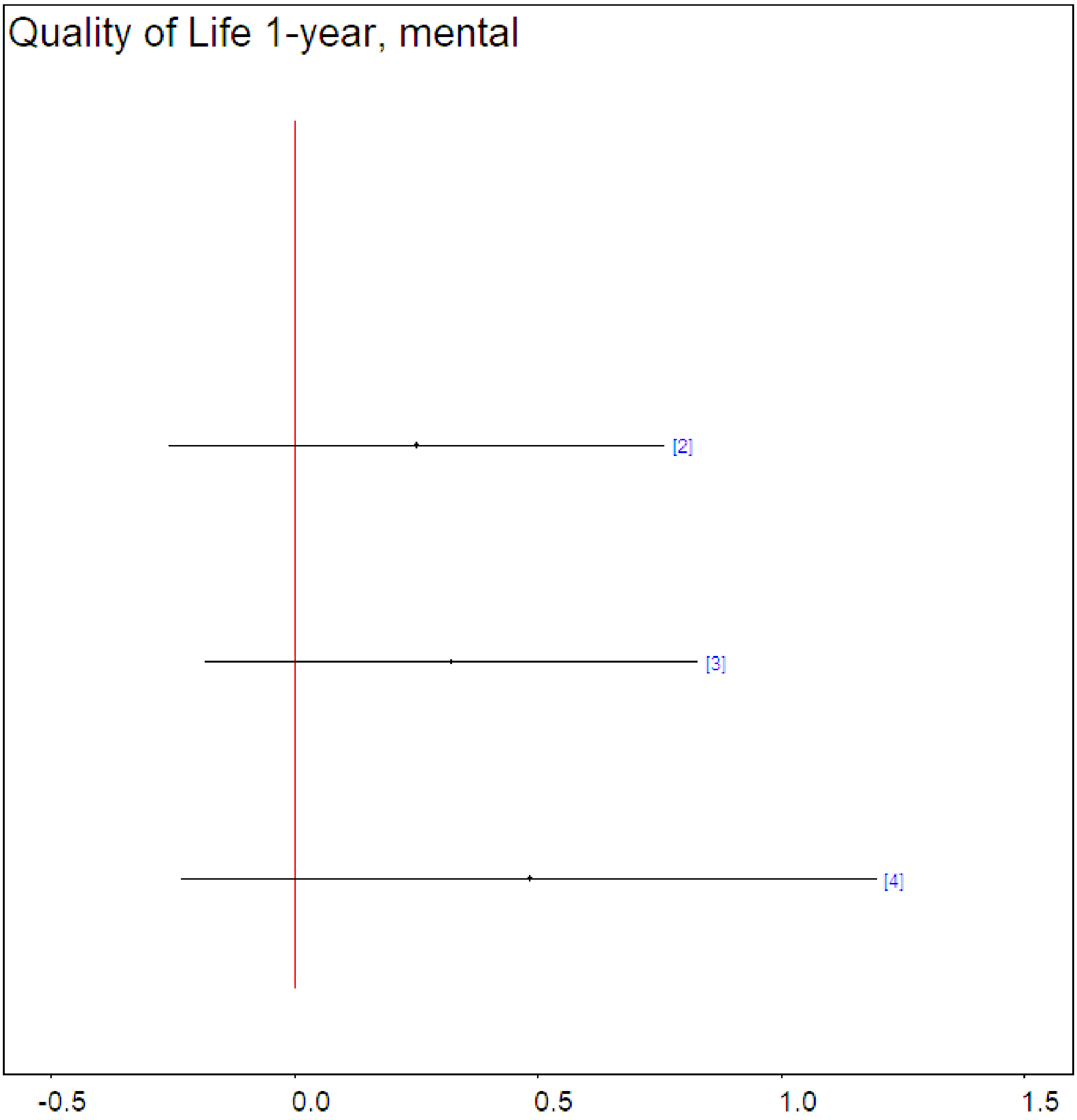

node	mean	sd	MC error	2.5%	median	97.5%
d[2]	0.2512	0.2597	7.392E-4	-0.2583	0.251	0.7603
d[3]	0.3224	0.258	7.369E-4	-0.1834	0.3223	0.8285
d[4]	0.4825	0.3649	0.001039	-0.2356	0.4825	1.197

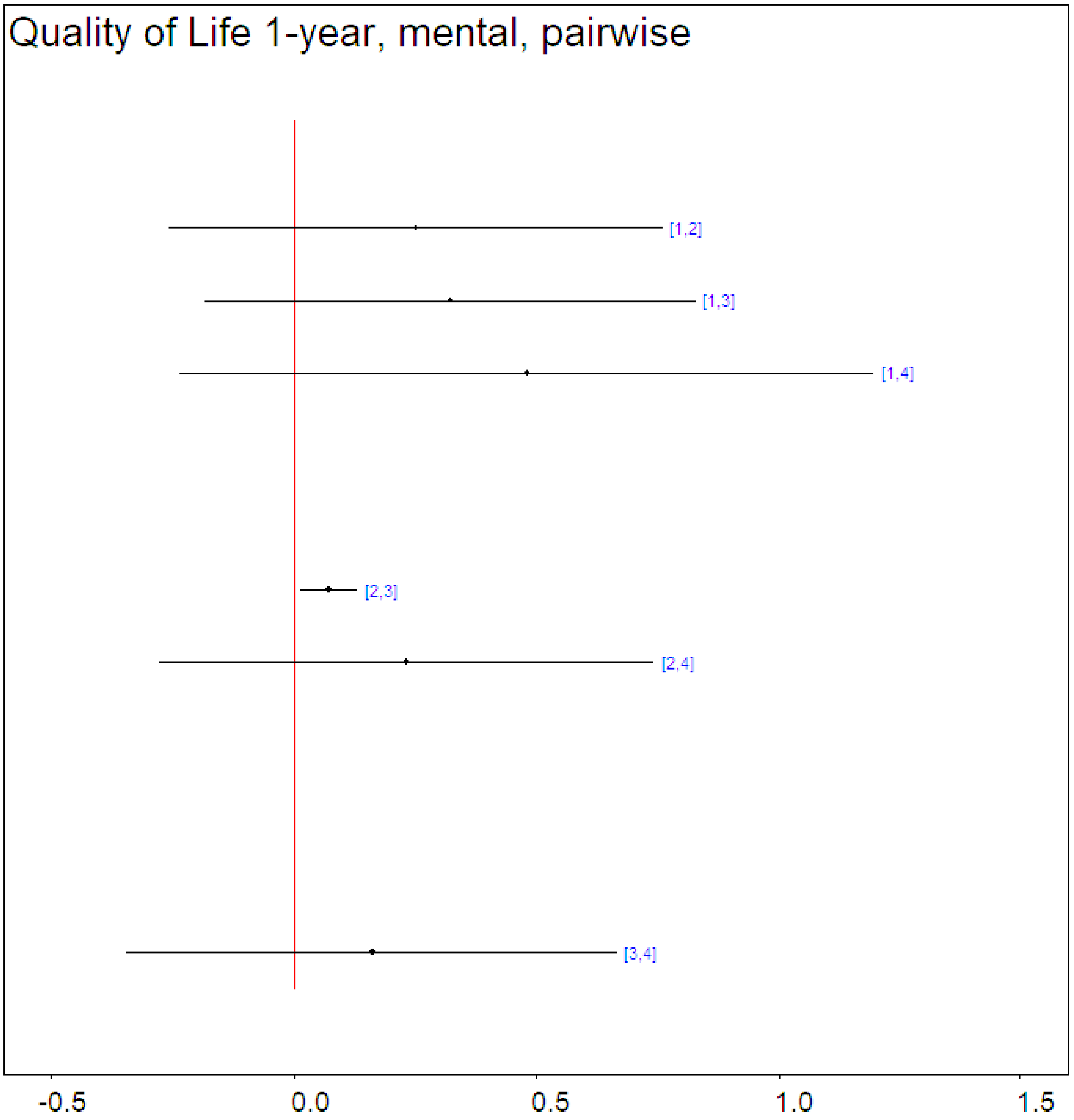

node	mean	sd	MC error	2.5%	median	97.5%
diff[1,2]	0.2512	0.2597	7.392E-4	-0.2583	0.251	0.7603
diff[1,3]	0.3224	0.258	7.369E-4	-0.1834	0.3223	0.8285
diff[1,4]	0.4825	0.3649	0.001039	-0.2356	0.4825	1.197
diff[2,3]	0.07117	0.03008	5.075E-5	0.01229	0.0711	0.1301
diff[2,4]	0.2313	0.2605	4.349E-4	-0.2789	0.2316	0.7422
diff[3,4]	0.1601	0.2587	4.327E-4	-0.3469	0.1602	0.6673

QoL 1-year bodily pain

6 studies

Legend:

1 LI
2 LSG
3 LRYGB
4 BPD-DS

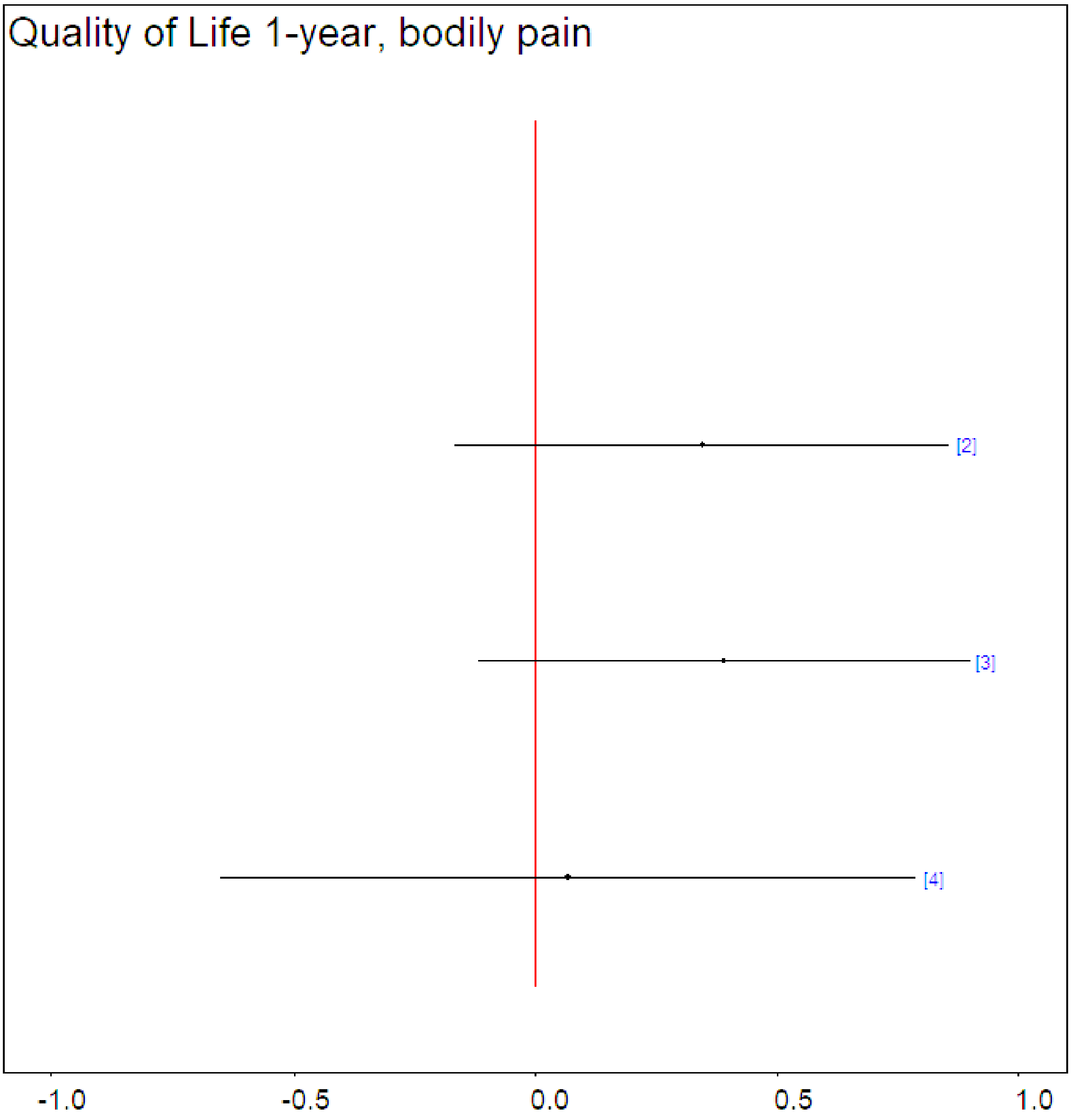

node	mean	sd	MC error	2.5%	median	97.5%
d[2]	0.3451	0.2606	7.406E-4	-0.166	0.3449	0.8558
d[3]	0.3899	0.2589	7.383E-4	-0.1174	0.3898	0.8977
d[4]	0.06905	0.3664	0.001042	-0.652	0.06909	0.786

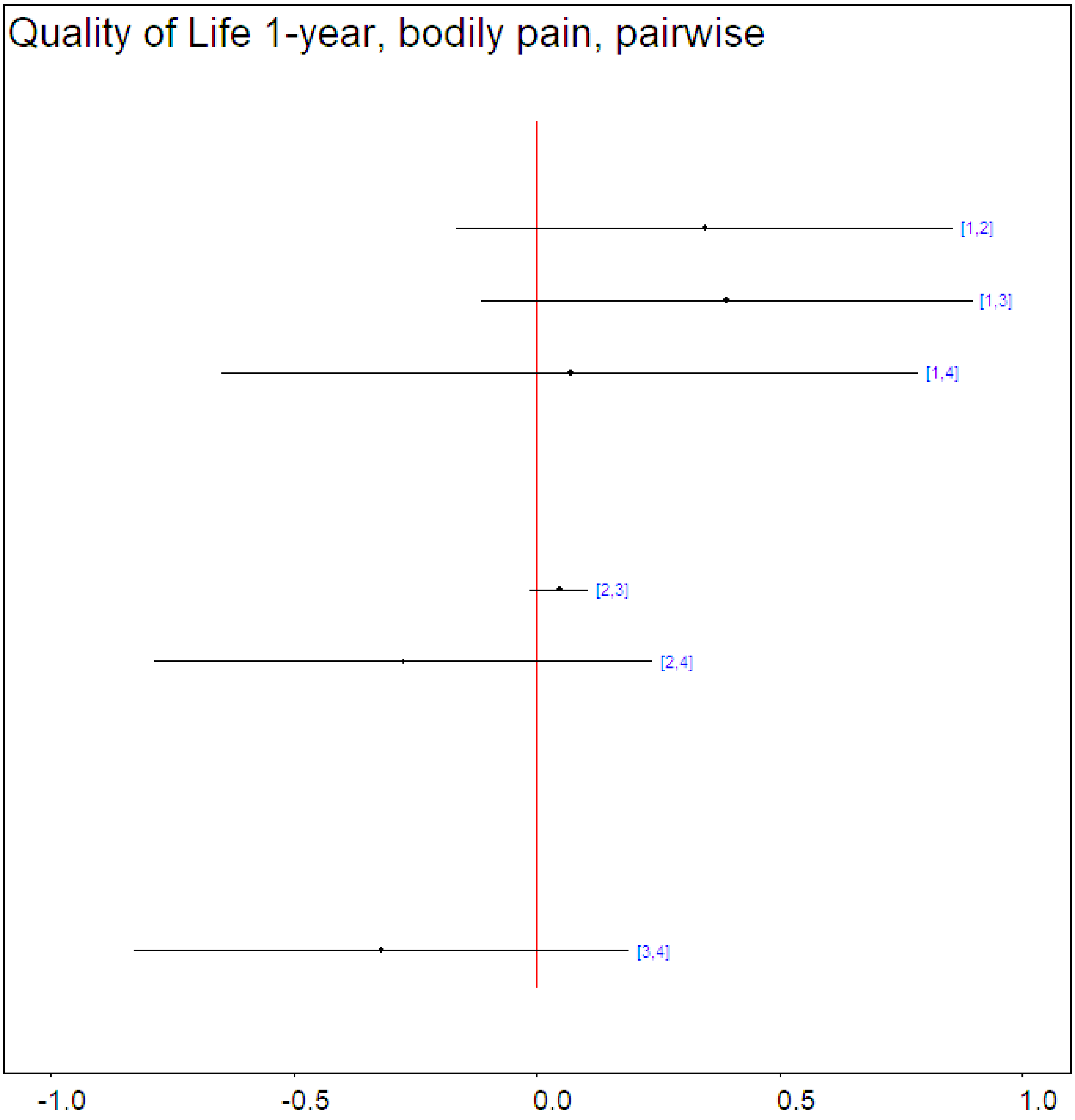

node	mean	sd	MC error	2.5%	median	97.5%
diff[1,2]	0.3451	0.2606	7.406E-4	-0.166	0.3449	0.8558
diff[1,3]	0.3899	0.2589	7.383E-4	-0.1174	0.3898	0.8977
diff[1,4]	0.06905	0.3664	0.001042	-0.652	0.06909	0.786
diff[2,3]	0.04483	0.03029	5.111E-5	-0.01446	0.04477	0.1042
diff[2,4]	-0.2761	0.2618	4.371E-4	-0.7889	-0.2757	0.2375
diff[3,4]	-0.3209	0.2601	4.349E-4	-0.8305	-0.3208	0.1889

Total QoL 2-years

15 studies

Legend:

1	LI
2	LSG
3	LRYGB
4	BPD-DS
5	LAGB
6	Banded-GB
7	Distal-GB
8	OAGB
9	LB-GB

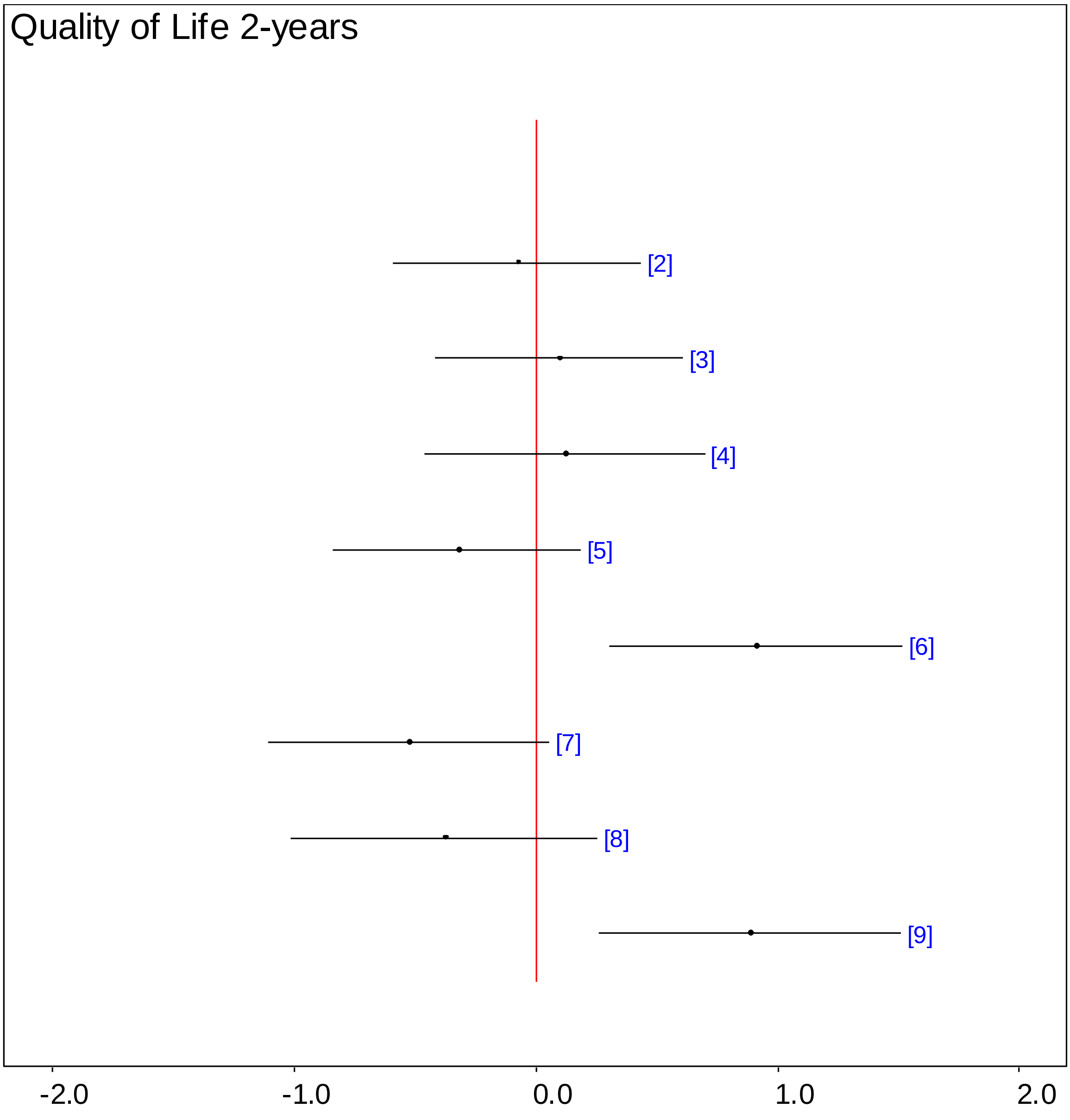

node	mean	sd	MC error	2.5%	median	97.5%
d[2]	-0.07122	0.2635	0.004246	-0.588	-0.06716	0.4361
d[3]	0.1026	0.2632	0.004239	-0.415	0.1065	0.6091
d[4]	0.1241	0.297	0.004279	-0.4596	0.1268	0.7007
d[5]	-0.3188	0.2636	0.004117	-0.8368	-0.3157	0.1897
d[6]	0.9172	0.3111	0.004283	0.3036	0.9192	1.52
d[7]	-0.523	0.2981	0.004289	-1.11	-0.521	0.05559
d[8]	-0.3739	0.3235	0.004287	-1.011	-0.372	0.2554
d[9]	0.8906	0.319	0.004292	0.2647	0.8932	1.512
Totresdev	19.14	6.431	0.01563	8.744	18.43	33.72

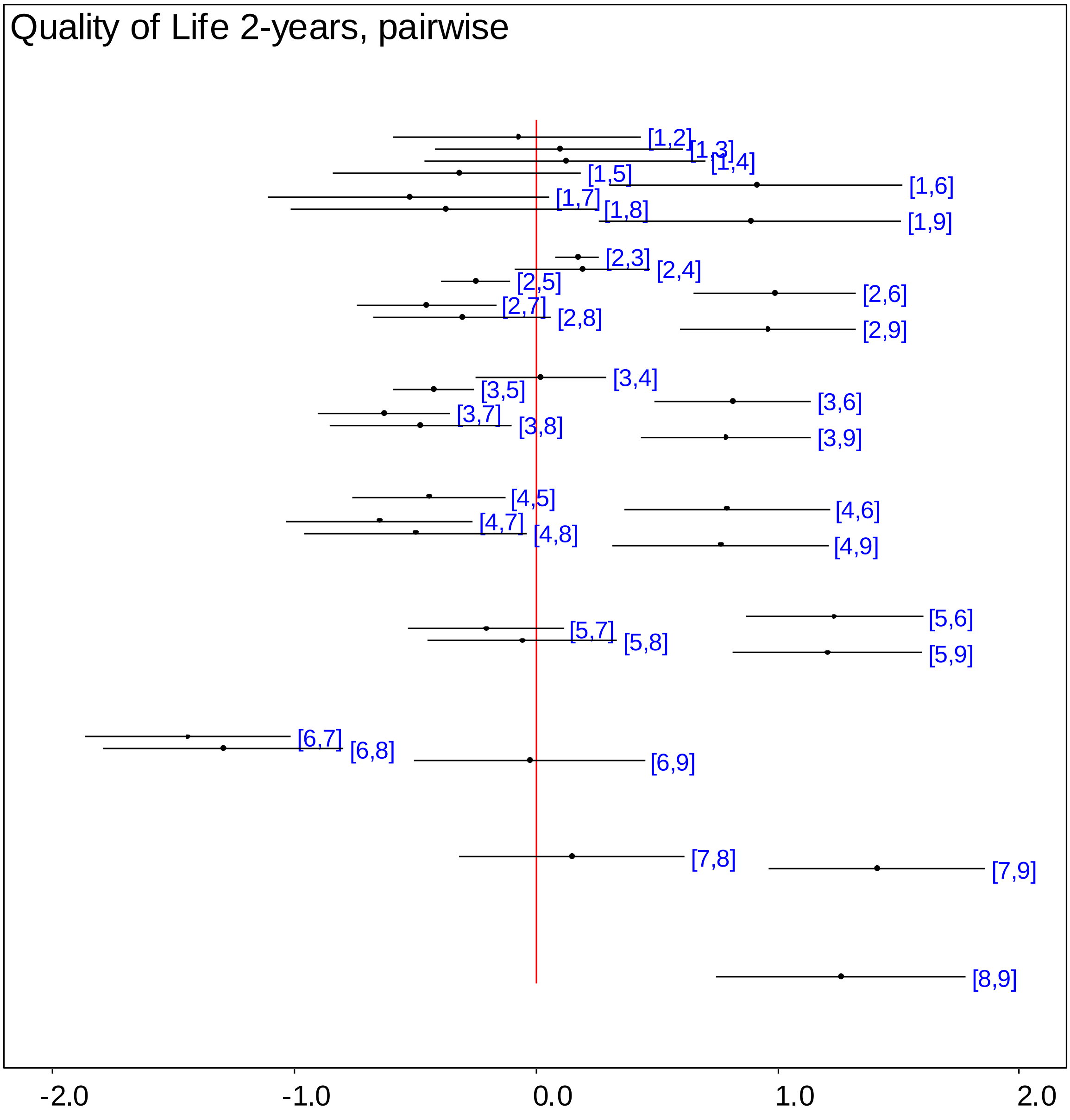

node	mean	sd	MC error	2.5%	median	97.5%
diff[1,2]	-0.07122	0.2635	0.004246	-0.588	-0.06716	0.4361
diff[1,3]	0.1026	0.2632	0.004239	-0.415	0.1065	0.6091
diff[1,4]	0.1241	0.297	0.004279	-0.4596	0.1268	0.7007
diff[1,5]	-0.3188	0.2636	0.004117	-0.8368	-0.3157	0.1897
diff[1,6]	0.9172	0.3111	0.004283	0.3036	0.9192	1.52
diff[1,7]	-0.523	0.2981	0.004289	-1.11	-0.521	0.05559
diff[1,8]	-0.3739	0.3235	0.004287	-1.011	-0.372	0.2554
diff[1,9]	0.8906	0.319	0.004292	0.2647	0.8932	1.512
diff[2,3]	0.1738	0.0459	7.244E-5	0.08395	0.1738	0.2639
diff[2,4]	0.1953	0.1437	2.442E-4	-0.0864	0.1955	0.4763
diff[2,5]	-0.2476	0.07381	1.755E-4	-0.3926	-0.2476	-0.1034
diff[2,6]	0.9884	0.1723	3.054E-4	0.6509	0.9881	1.326
diff[2,7]	-0.4517	0.1467	2.633E-4	-0.7393	-0.4519	-0.1633
diff[2,8]	-0.3027	0.1868	3.119E-4	-0.6695	-0.3024	0.06321
diff[2,9]	0.9619	0.1855	3.059E-4	0.5971	0.9622	1.326
diff[3,4]	0.02151	0.1386	2.298E-4	-0.2506	0.02183	0.2922
diff[3,5]	-0.4214	0.08635	1.969E-4	-0.5909	-0.4214	-0.2521
diff[3,6]	0.8146	0.166	2.868E-4	0.4901	0.8147	1.14
diff[3,7]	-0.6255	0.1392	2.386E-4	-0.8985	-0.6256	-0.3516
diff[3,8]	-0.4765	0.1925	3.292E-4	-0.8545	-0.4763	-0.09883
diff[3,9]	0.7881	0.1797	2.936E-4	0.434	0.7884	1.141
diff[4,5]	-0.4429	0.1615	3.244E-4	-0.7589	-0.443	-0.1265
diff[4,6]	0.7931	0.2162	3.769E-4	0.3705	0.7926	1.217
diff[4,7]	-0.647	0.1963	3.258E-4	-1.031	-0.647	-0.2616
diff[4,8]	-0.498	0.2358	3.971E-4	-0.9594	-0.4977	-0.03558
diff[4,9]	0.7665	0.2269	3.853E-4	0.3208	0.7666	1.212
diff[5,6]	1.236	0.1872	3.69E-4	0.8698	1.236	1.603
diff[5,7]	-0.2041	0.1639	3.435E-4	-0.5254	-0.2041	0.1171
diff[5,8]	-0.05506	0.2009	3.675E-4	-0.4503	-0.05507	0.3394
diff[5,9]	1.209	0.1996	3.746E-4	0.8175	1.21	1.6
diff[6,7]	-1.44	0.2167	3.847E-4	-1.865	-1.44	-1.014
diff[6,8]	-1.291	0.254	4.41E-4	-1.79	-1.291	-0.7932
diff[6,9]	-0.02654	0.2444	4.137E-4	-0.5061	-0.02621	0.4528
diff[7,8]	0.1491	0.2373	4.155E-4	-0.3164	0.1484	0.6156
diff[7,9]	1.414	0.2275	3.764E-4	0.9672	1.414	1.861
diff[8,9]	1.265	0.2633	4.406E-4	0.7486	1.264	1.779

QoL 2-years physical

6 studies

Legend:

1 LI
2 LSG
3 LRYGB
4 BPD-DS
5 LAGB
6 Distal-GB

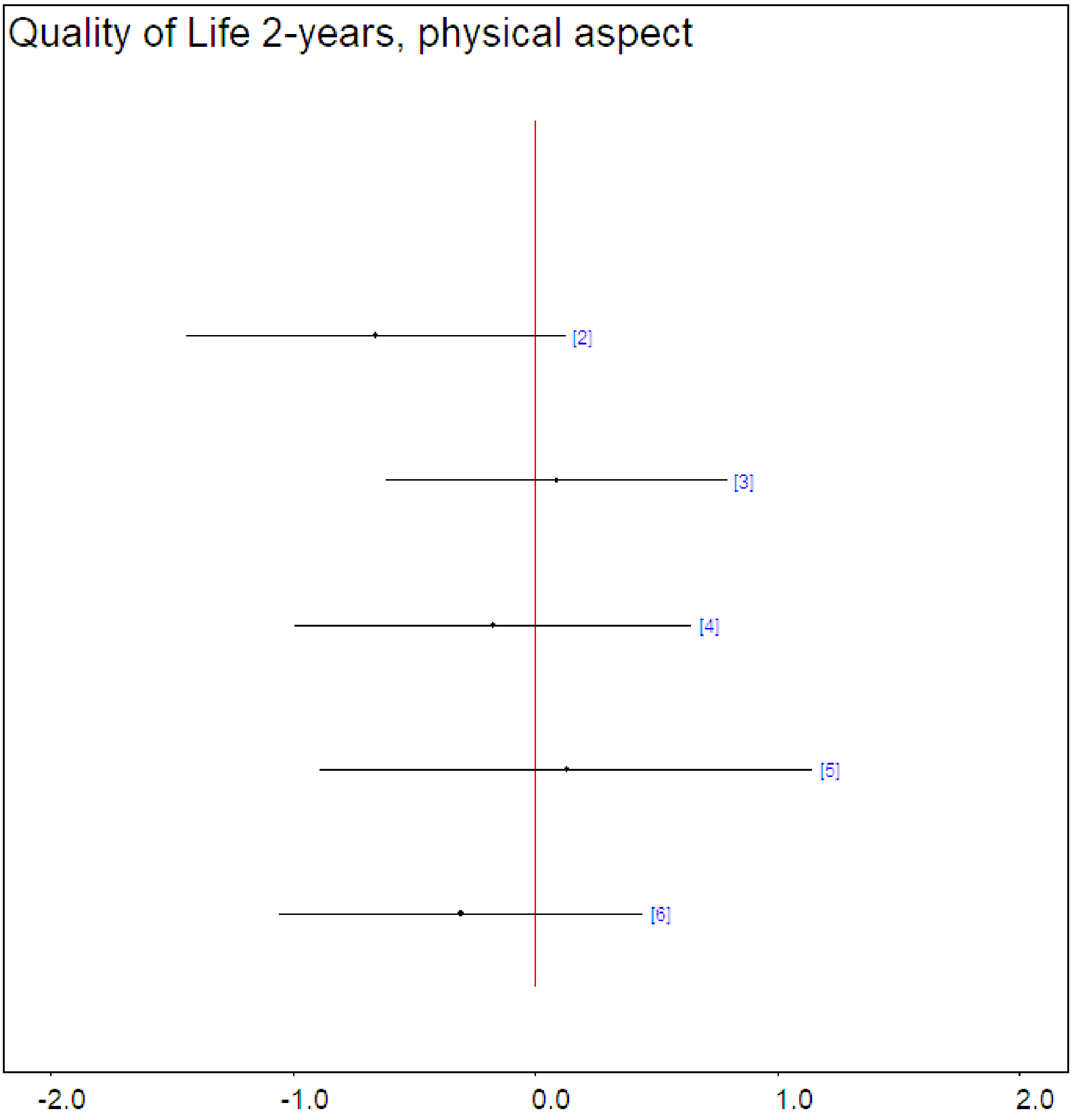

node	mean	sd	MC error	2.5%	median	97.5%
d[2]	-0.6613	0.4008	0.003394	-1.446	-0.6614	0.1234
d[3]	0.08599	0.3584	0.00316	-0.6157	0.08609	0.7897
d[4]	-0.1762	0.4178	0.003379	-0.9956	-0.1761	0.6436
d[5]	0.1266	0.518	0.003542	-0.8901	0.1265	1.143

d[6]	-0.3069	0.3833	0.003267	-1.057	-0.3071	0.4458

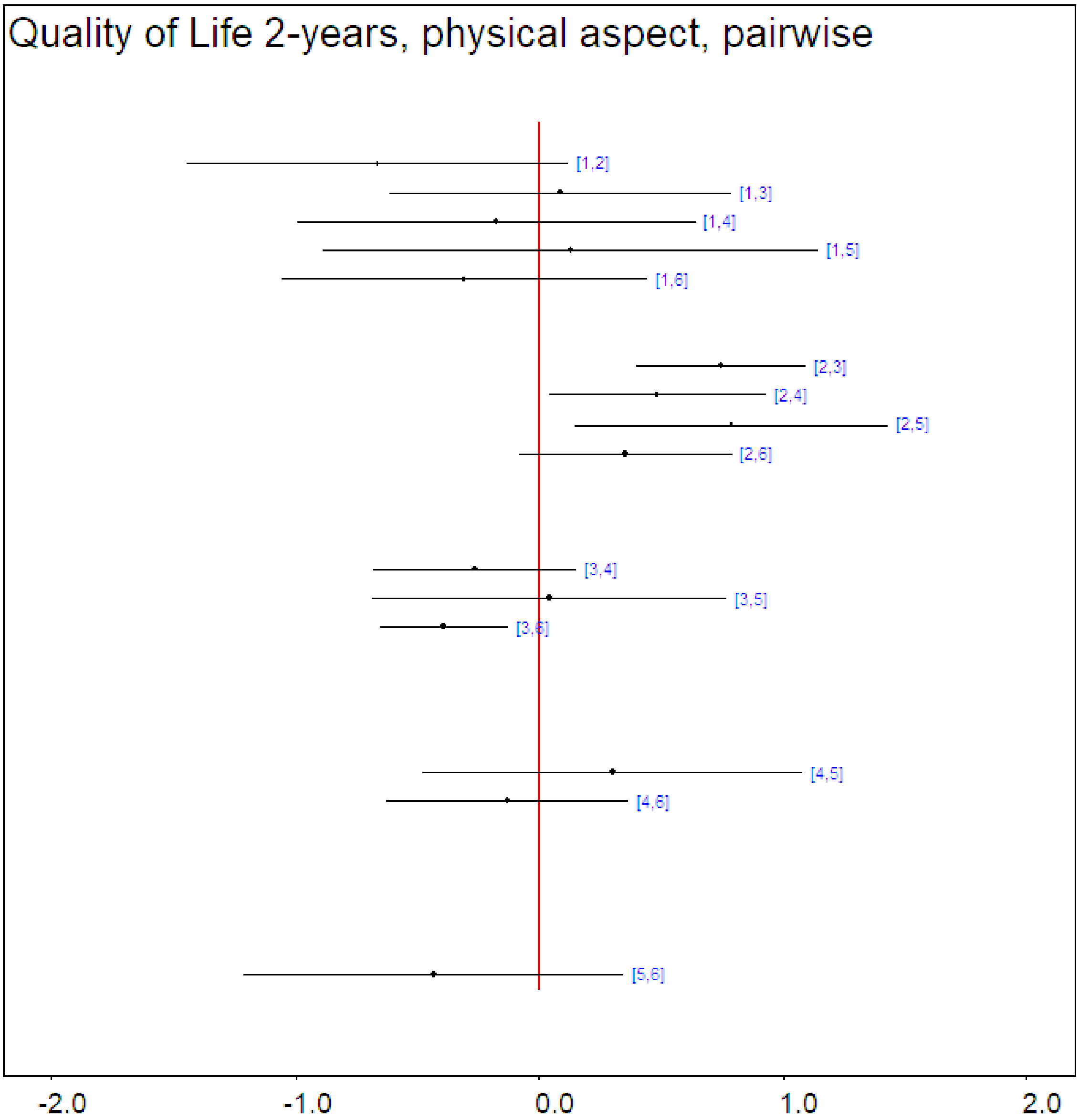

node	mean	sd	MC error	2.5%	median	97.5%
diff[1,2]	-0.6613	0.4008	0.003394	-1.446	-0.6614	0.1234
diff[1,3]	0.08599	0.3584	0.00316	-0.6157	0.08609	0.7897
diff[1,4]	-0.1762	0.4178	0.003379	-0.9956	-0.1761	0.6436
diff[1,5]	0.1266	0.518	0.003542	-0.8901	0.1265	1.143
diff[1,6]	-0.3069	0.3833	0.003267	-1.057	-0.3071	0.4458
diff[2,3]	0.7473	0.1782	4.022E-4	0.398	0.7471	1.097
diff[2,4]	0.4852	0.2273	3.558E-4	0.03917	0.4856	0.9313
diff[2,5]	0.7879	0.3281	5.542E-4	0.1455	0.7879	1.432
diff[2,6]	0.3544	0.2238	4.629E-4	-0.08501	0.3545	0.7929

diff[3,4]	-0.2621	0.2134	4.088E-4	-0.6814	-0.2616	0.1552
diff[3,5]	0.04057	0.3734	7.996E-4	-0.69	0.04072	0.7723
diff[3,6]	-0.3929	0.135	2.242E-4	-0.6582	-0.3927	-0.1286
diff[4,5]	0.3027	0.3987	7.441E-4	-0.4769	0.3027	1.084
diff[4,6]	-0.1308	0.2527	4.729E-4	-0.6254	-0.1302	0.3655
diff[5,6]	-0.4335	0.3972	8.267E-4	-1.212	-0.4338	0.3453

Qol 2-years emotional

4 studies

Legend:

1 LSG
2 LRYGB
3 BPD-DS
4 LAGB
5 Distal-GB

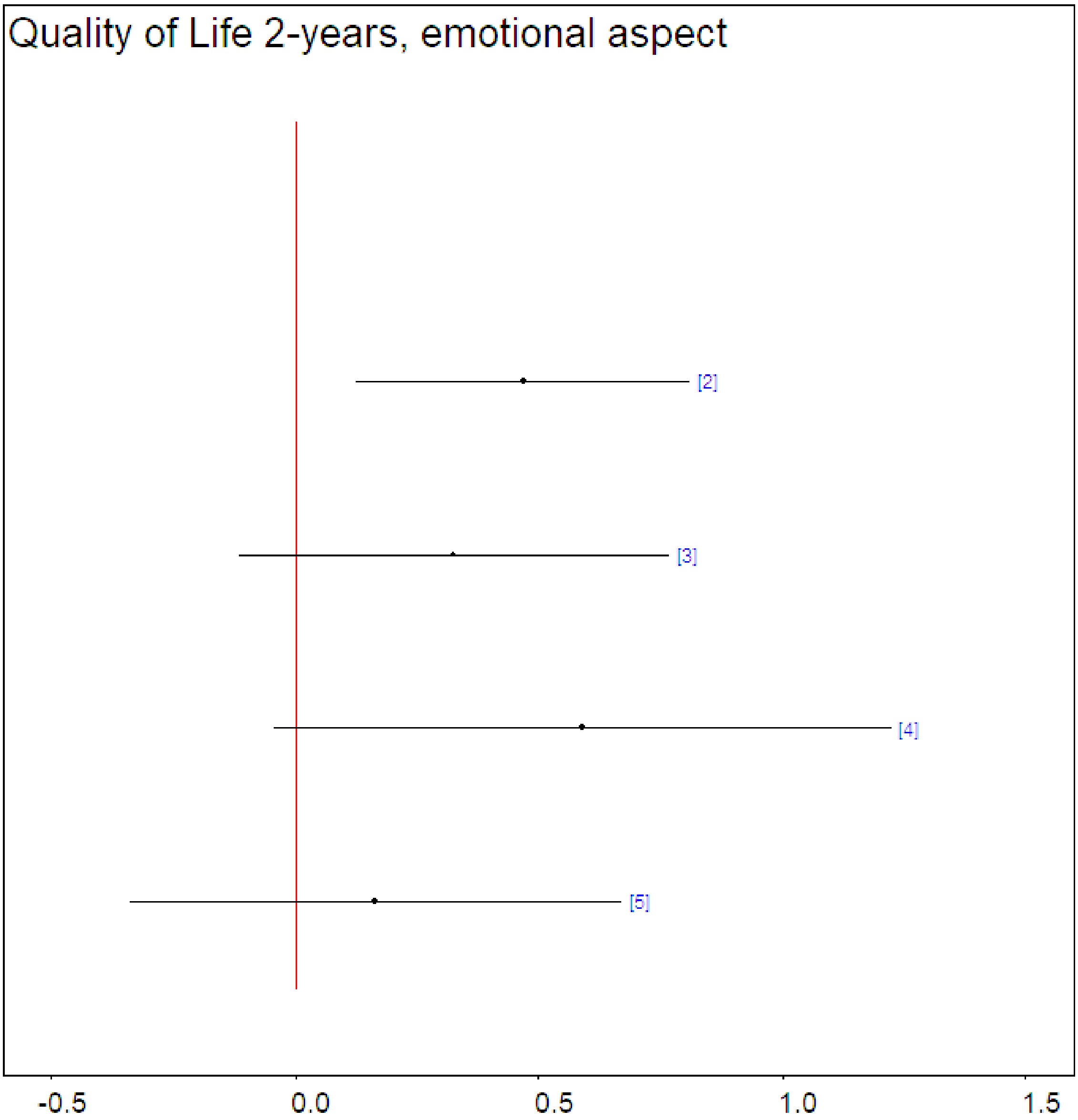

node	mean	sd	MC error	2.5%	median	97.5%
d[2]	0.4681	0.1751	5.344E-4	0.1253	0.4681	0.8109
d[3]	0.3252	0.2256	5.337E-4	-0.116	0.3251	0.7681
d[4]	0.5897	0.3231	5.287E-4	-0.04418	0.5897	1.223

d[5]	0.1633	0.2574	7.573E-4	-0.3398	0.1631	0.6696

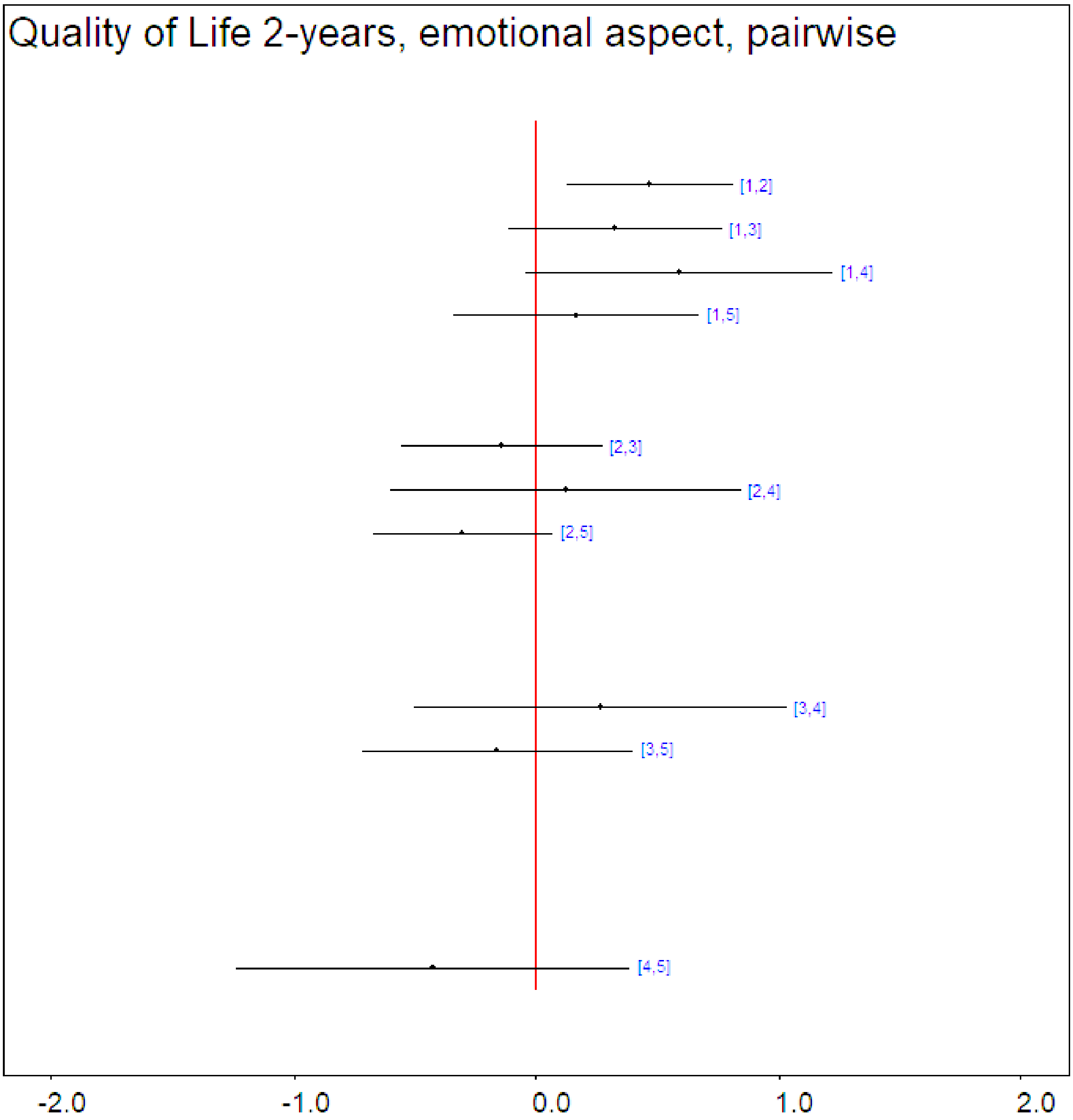

node	mean	sd	MC error	2.5%	median	97.5%
diff[1,2]	0.4681	0.1751	5.344E-4	0.1253	0.4681	0.8109
diff[1,3]	0.3252	0.2256	5.337E-4	-0.116	0.3251	0.7681
diff[1,4]	0.5897	0.3231	5.287E-4	-0.04418	0.5897	1.223
diff[1,5]	0.1633	0.2574	7.573E-4	-0.3398	0.1631	0.6696
diff[2,3]	-0.1429	0.2122	3.315E-4	-0.5595	-0.1429	0.273
diff[2,4]	0.1216	0.3673	7.459E-4	-0.5979	0.1217	0.8425
diff[2,5]	-0.3047	0.1891	3.226E-4	-0.6758	-0.3045	0.06634
diff[3,4]	0.2645	0.3939	7.479E-4	-0.5085	0.2642	1.034
diff[3,5]	-0.1619	0.2838	5.236E-4	-0.7189	-0.1619	0.3957
diff[4,5]	-0.4264	0.4134	9.218E-4	-1.235	-0.427	0.3841

QoL 2-years general health

4 studies

Legend:

1 LSG
2 LRYGB
3 BPD-DS
4 LAGB
5 Distal-GB

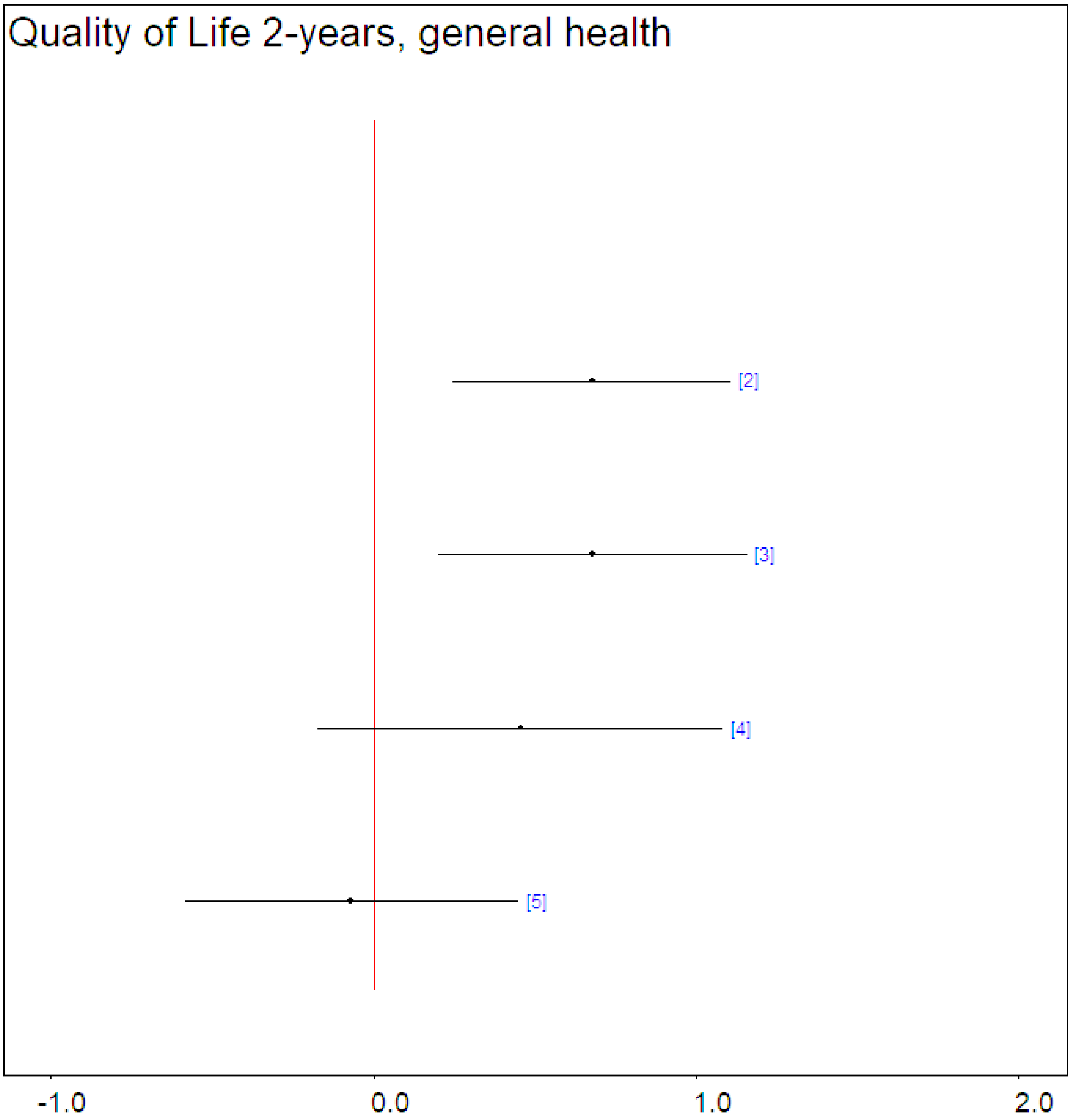

node	mean	sd	MC error	2.5%	median	97.5%
d[2]	0.6739	0.2206	9.61E-4	0.2431	0.6738	1.106
d[3]	0.6771	0.2451	7.711E-4	0.1983	0.6769	1.159
d[4]	0.4536	0.3201	5.238E-4	-0.1744	0.4536	1.081

d[5]	-0.07028	0.2628	0.001128	-0.5839	-0.07059	0.4467

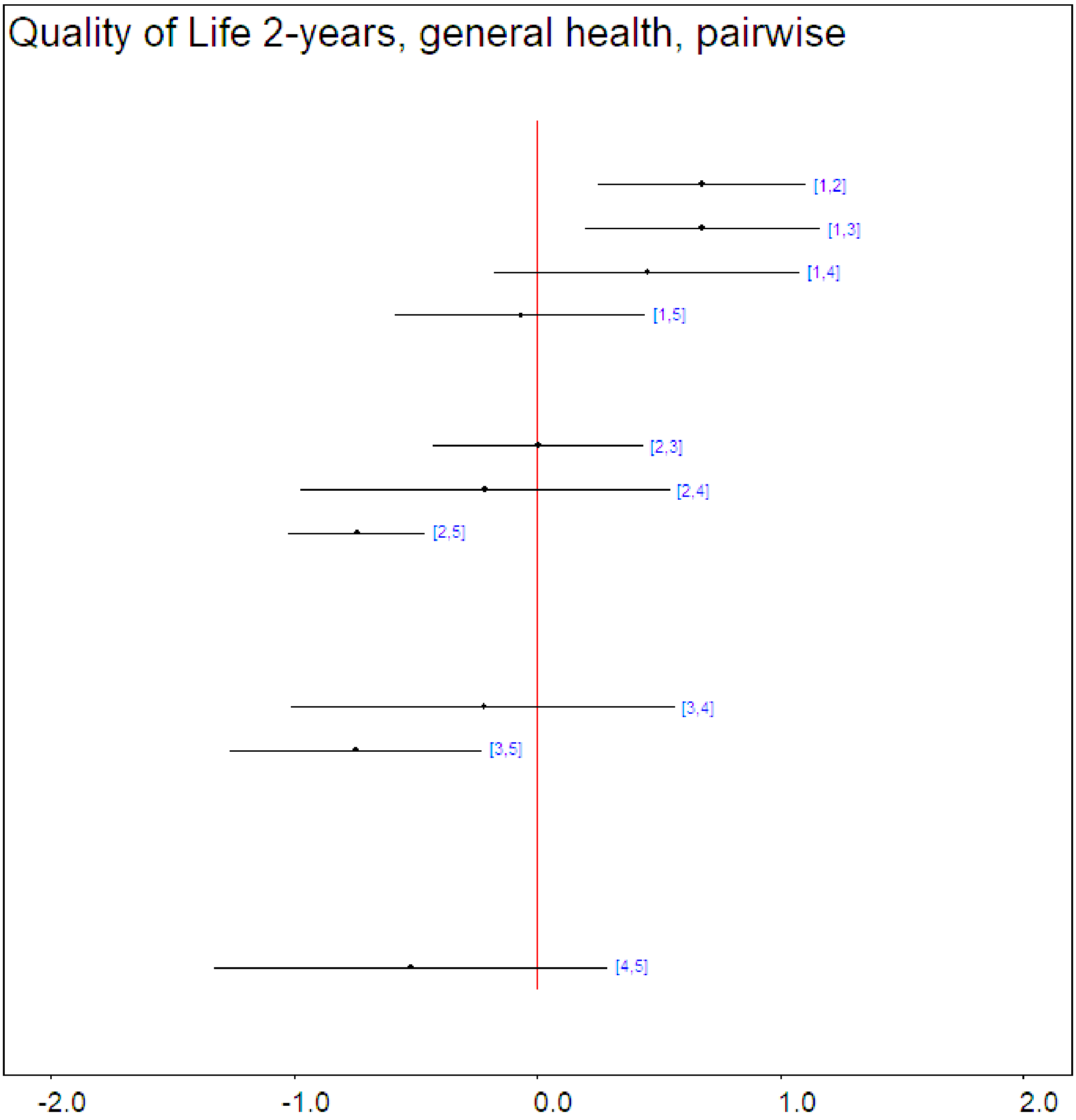

node	mean	sd	MC error	2.5%	median	97.5%
diff[1,2]	0.6739	0.2206	9.61E-4	0.2431	0.6738	1.106
diff[1,3]	0.6771	0.2451	7.711E-4	0.1983	0.6769	1.159
diff[1,4]	0.4536	0.3201	5.238E-4	-0.1744	0.4536	1.081
diff[1,5]	-0.07028	0.2628	0.001128	-0.5839	-0.07059	0.4467
diff[2,3]	0.003115	0.2204	4.158E-4	-0.4293	0.003302	0.4356
diff[2,4]	-0.2203	0.3884	0.001089	-0.9798	-0.2203	0.5412
diff[2,5]	-0.7442	0.1433	2.446E-4	-1.026	-0.744	-0.4629
diff[3,4]	-0.2234	0.4029	9.279E-4	-1.014	-0.224	0.5642
diff[3,5]	-0.7473	0.2625	5.761E-4	-1.263	-0.7472	-0.2321
diff[4,5]	-0.5239	0.4142	0.00124	-1.335	-0.5245	0.2883

QoL 2-years vitality

3 studies

Legend:

1 LSG
2 LRYGB
3 BPD-DS
4 LAGB
5 Distal-GB

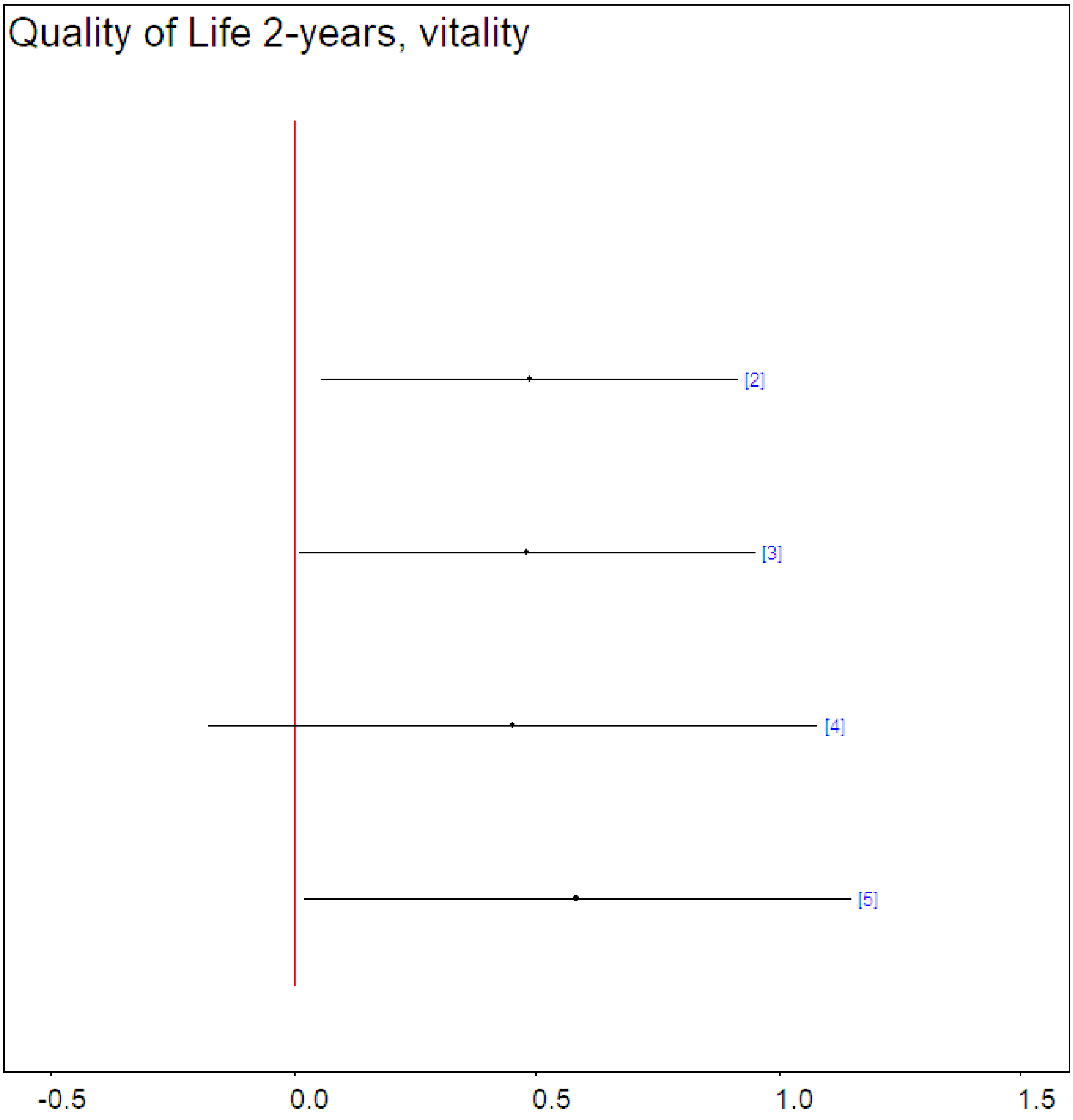

node	mean	sd	MC error	2.5%	median	97.5%
d[2]	0.4859	0.2188	7.917E-4	0.05769	0.4858	0.9149
d[3]	0.4794	0.2406	6.747E-4	0.008673	0.4791	0.9523
d[4]	0.4507	0.32	5.237E-4	-0.1772	0.4507	1.078
d[5]	0.5822	0.2881	0.001008	0.0191	0.5816	1.149

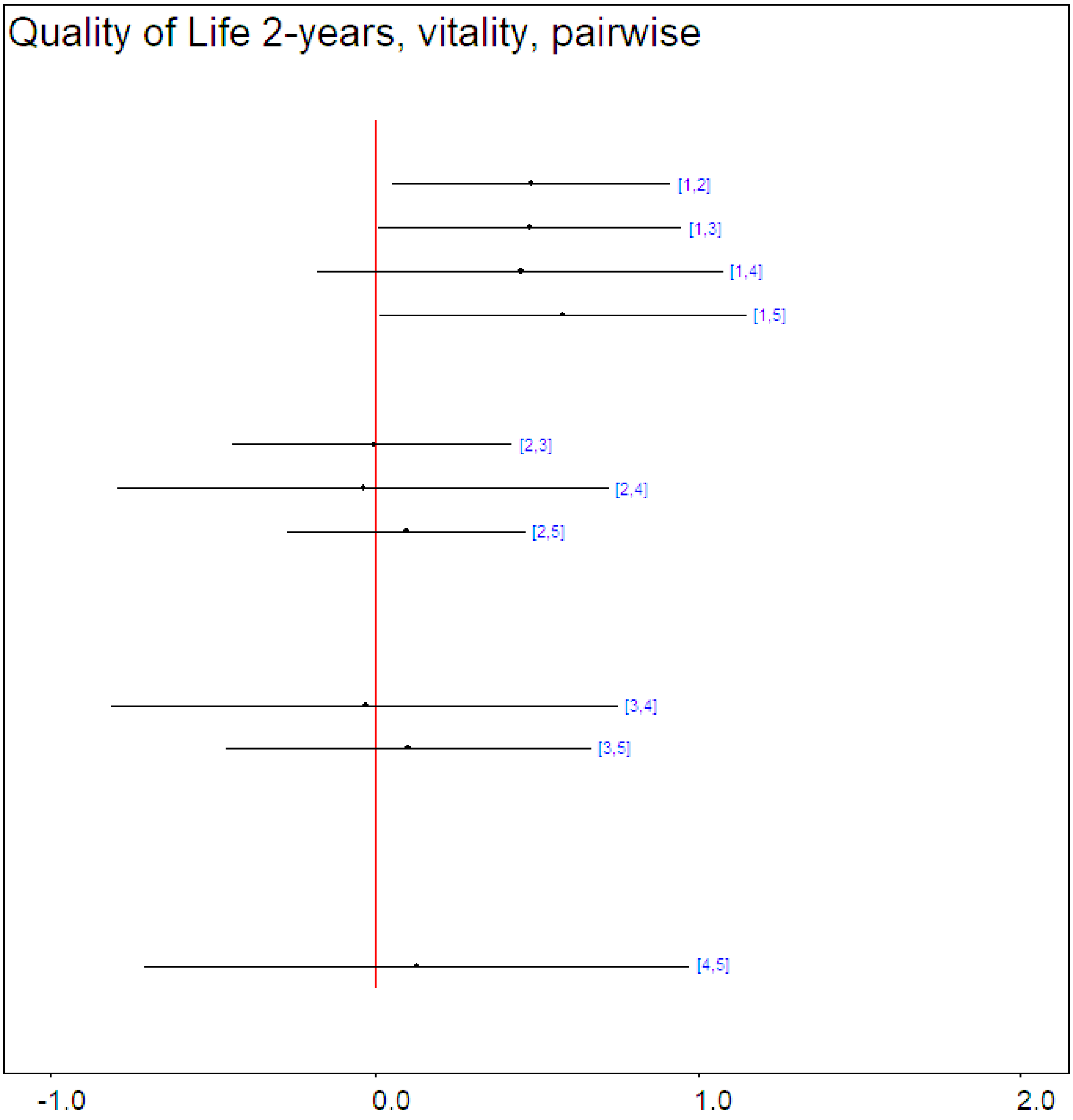

node	mean	sd	MC error	2.5%	median	97.5%
diff[1,2]	0.4859	0.2188	7.917E-4	0.05769	0.4858	0.9149
diff[1,3]	0.4794	0.2406	6.747E-4	0.008673	0.4791	0.9523
diff[1,4]	0.4507	0.32	5.237E-4	-0.1772	0.4507	1.078
diff[1,5]	0.5822	0.2881	0.001008	0.0191	0.5816	1.149
diff[2,3]	-0.006498	0.219	3.698E-4	-0.4365	-0.006304	0.4231
diff[2,4]	-0.03517	0.3874	9.432E-4	-0.7937	-0.03452	0.7251
diff[2,5]	0.09633	0.1881	3.209E-4	-0.2727	0.09659	0.4654
diff[3,4]	-0.02867	0.4001	8.501E-4	-0.8137	-0.02921	0.7525
diff[3,5]	0.1028	0.2882	5.824E-4	-0.4628	0.1029	0.6686
diff[4,5]	0.1315	0.4309	0.001133	-0.7118	0.1312	0.9757

QoL 2-years physical role

3 studies

Legend:

1 LSG
2 LRYGB
3 BPD-DS
4 LAGB
5 Distal-GB

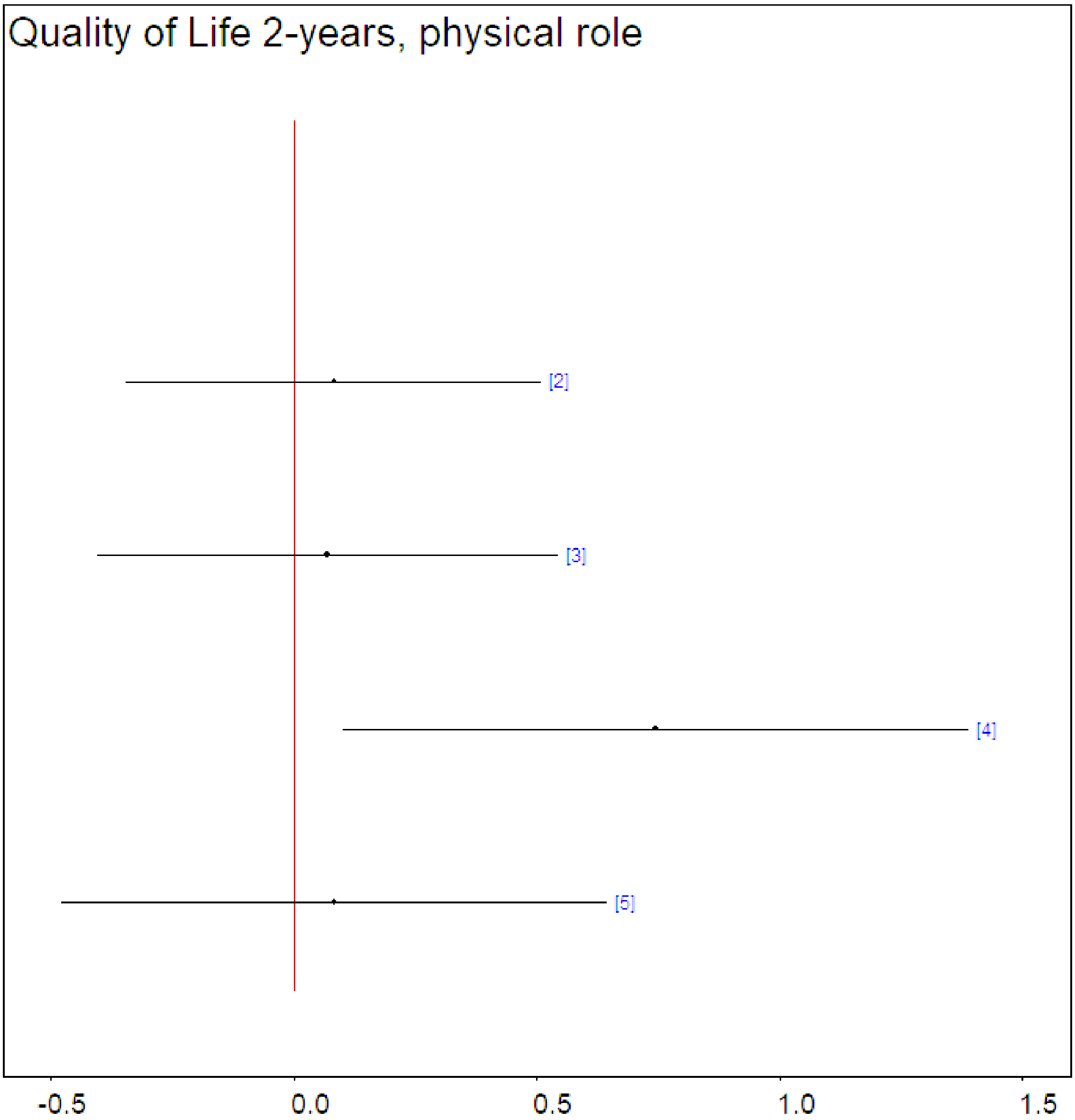

node	mean	sd	MC error	2.5%	median	97.5%
d[2]	0.08099	0.2179	7.848E-4	-0.3454	0.08079	0.5084
d[3]	0.06674	0.2418	6.725E-4	-0.406	0.0666	0.542
d[4]	0.745	0.3274	5.358E-4	0.1026	0.745	1.387
d[5]	0.08076	0.2874	0.001001	-0.4809	0.08013	0.6457

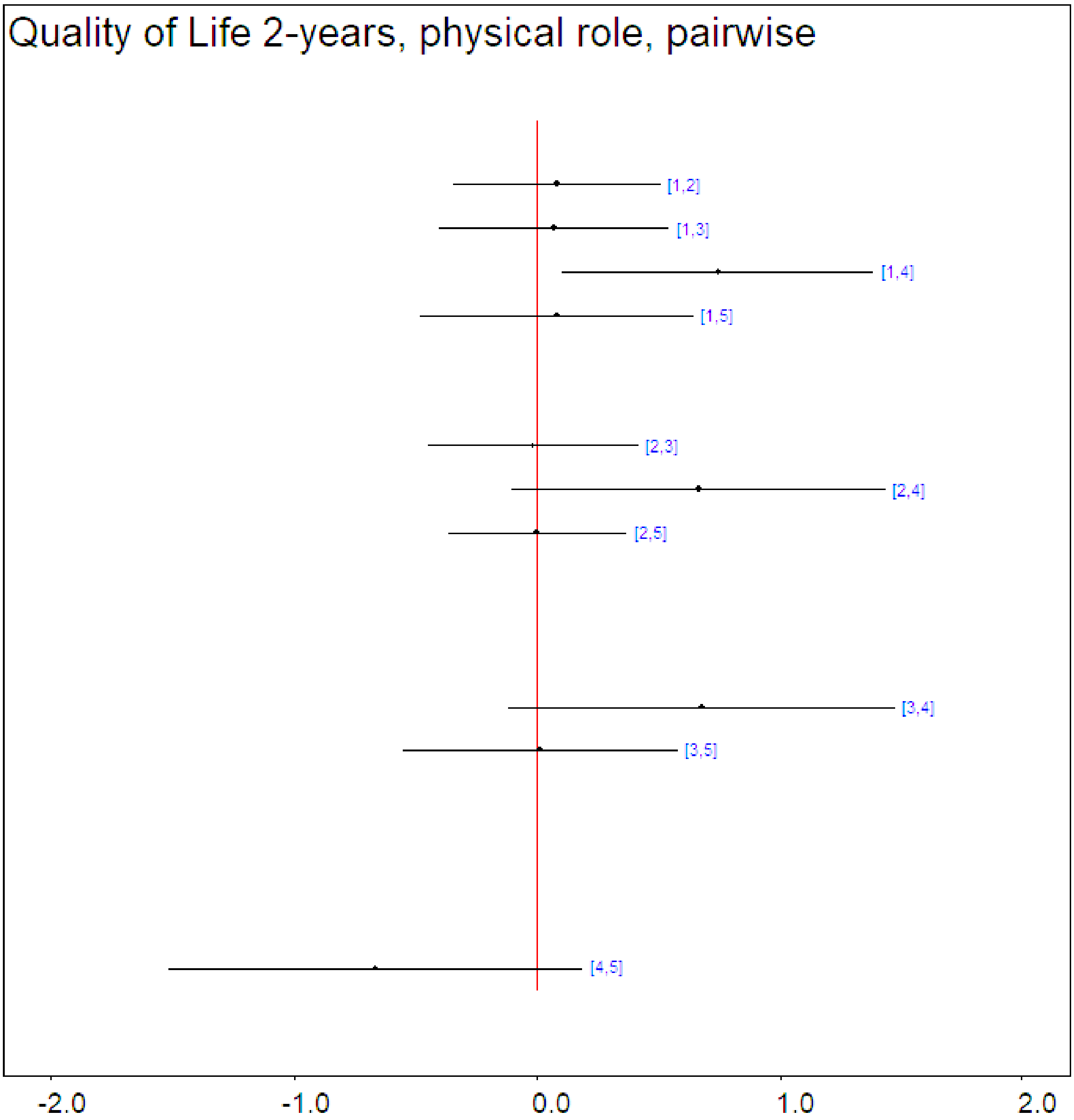

node	mean	sd	MC error	2.5%	median	97.5%
diff[1,2]	0.08099	0.2179	7.848E-4	-0.3454	0.08079	0.5084
diff[1,3]	0.06674	0.2418	6.725E-4	-0.406	0.0666	0.542
diff[1,4]	0.745	0.3274	5.358E-4	0.1026	0.745	1.387
diff[1,5]	0.08076	0.2874	0.001001	-0.4809	0.08013	0.6457
diff[2,3]	-0.01425	0.2206	3.711E-4	-0.4474	-0.01408	0.4183
diff[2,4]	0.664	0.393	9.441E-4	-0.1057	0.6645	1.435
diff[2,5]	-2.367E-4	0.1879	3.207E-4	-0.3691	2.827E-5	0.3686
diff[3,4]	0.6783	0.4068	8.558E-4	-0.1196	0.6776	1.472
diff[3,5]	0.01401	0.2894	5.823E-4	-0.5536	0.01414	0.5821
diff[4,5]	-0.6642	0.4359	0.001133	-1.517	-0.6647	0.1901

QoL 2-years social

5 studies

Legend:

1 LSG
2 LRYGB
3 BPD-DS
4 LAGB
5 Distal-GB

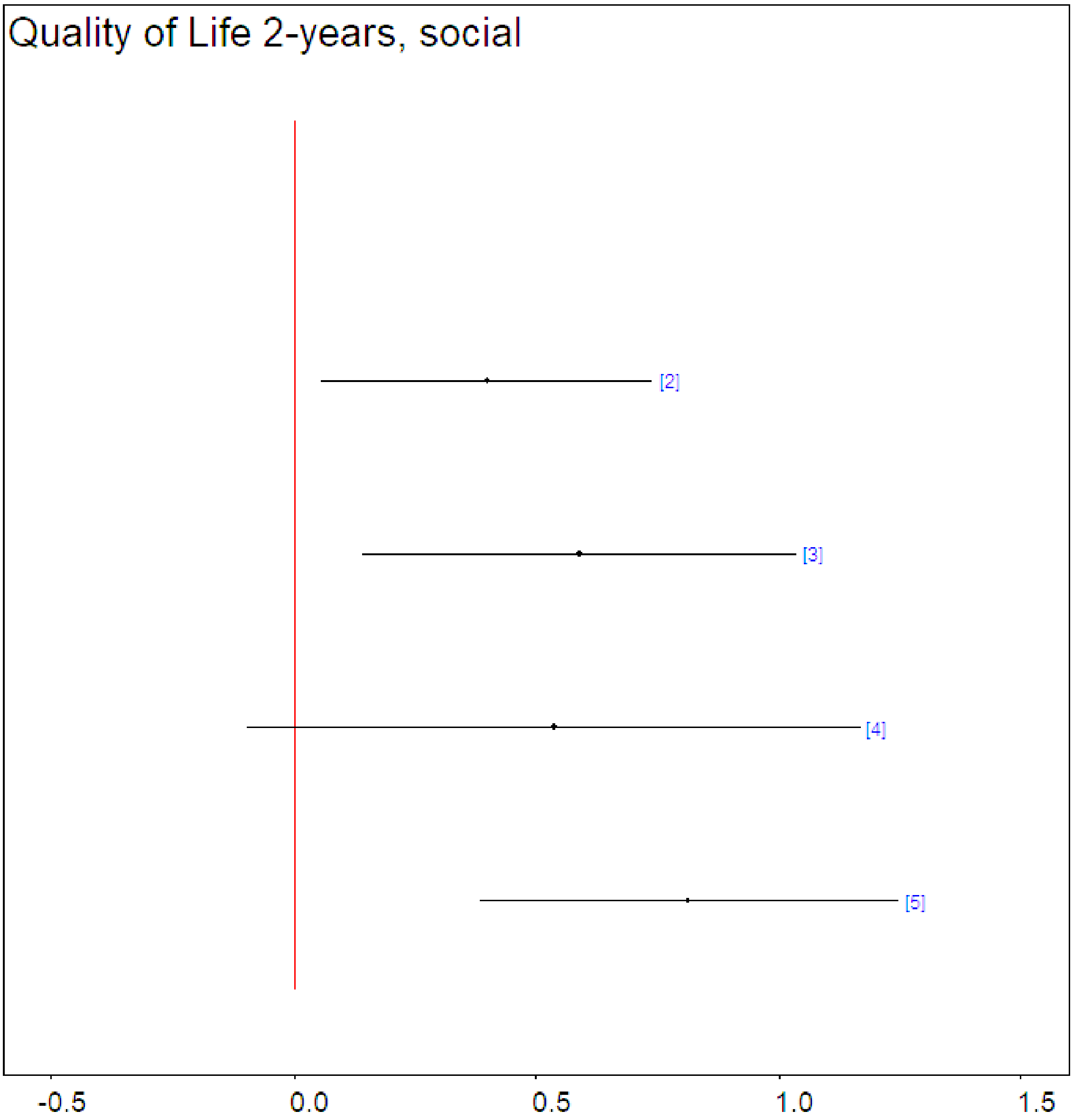

node	mean	sd	MC error	2.5%	median	97.5%
d[2]	0.3975	0.1747	6.542E-4	0.05601	0.3972	0.7399
d[3]	0.5883	0.2285	5.921E-4	0.1417	0.5882	1.037
d[4]	0.536	0.3218	5.266E-4	-0.09535	0.536	1.167

d[5]	0.8139	0.2204	8.136E-4	0.3837	0.8137	1.247

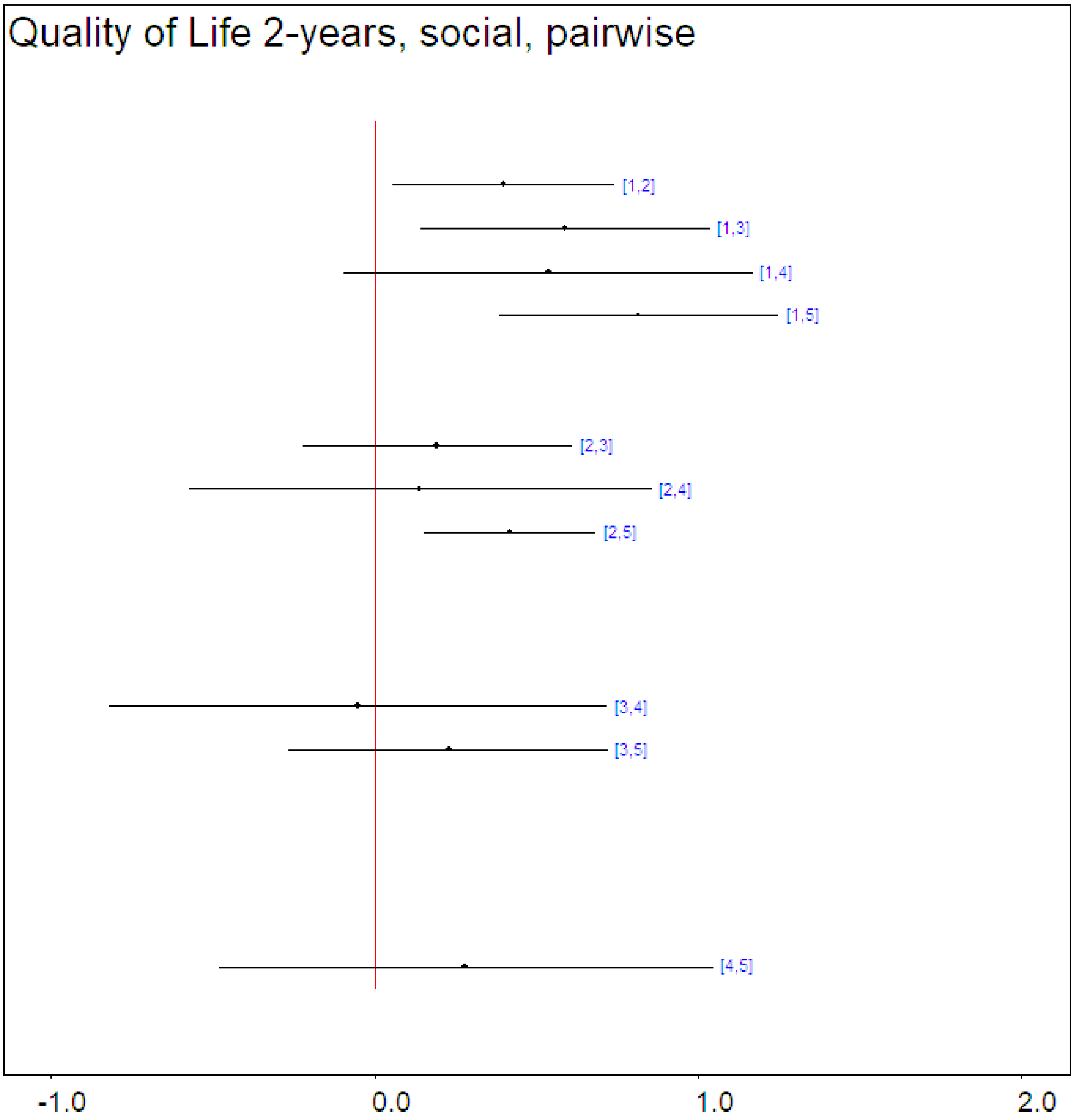

node	mean	sd	MC error	2.5%	median	97.5%
diff[1,2]	0.3975	0.1747	6.542E-4	0.05601	0.3972	0.7399
diff[1,3]	0.5883	0.2285	5.921E-4	0.1417	0.5882	1.037
diff[1,4]	0.536	0.3218	5.266E-4	-0.09535	0.536	1.167
diff[1,5]	0.8139	0.2204	8.136E-4	0.3837	0.8137	1.247
diff[2,3]	0.1908	0.2135	3.603E-4	-0.2283	0.1909	0.6097
diff[2,4]	0.1385	0.366	8.342E-4	-0.5782	0.1386	0.8563
diff[2,5]	0.4164	0.1349	2.301E-4	0.1517	0.4166	0.6811
diff[3,4]	-0.05229	0.3945	7.889E-4	-0.8254	-0.05261	0.7188
diff[3,5]	0.2256	0.2522	4.934E-4	-0.2697	0.2256	0.7206
diff[4,5]	0.2779	0.3902	9.663E-4	-0.4853	0.2776	1.044

QoL 2-years mental

3 studies

Legend:

1 LSG
2 LRYGB
3 BPD-DS
4 LAGB
5 Distal-GB

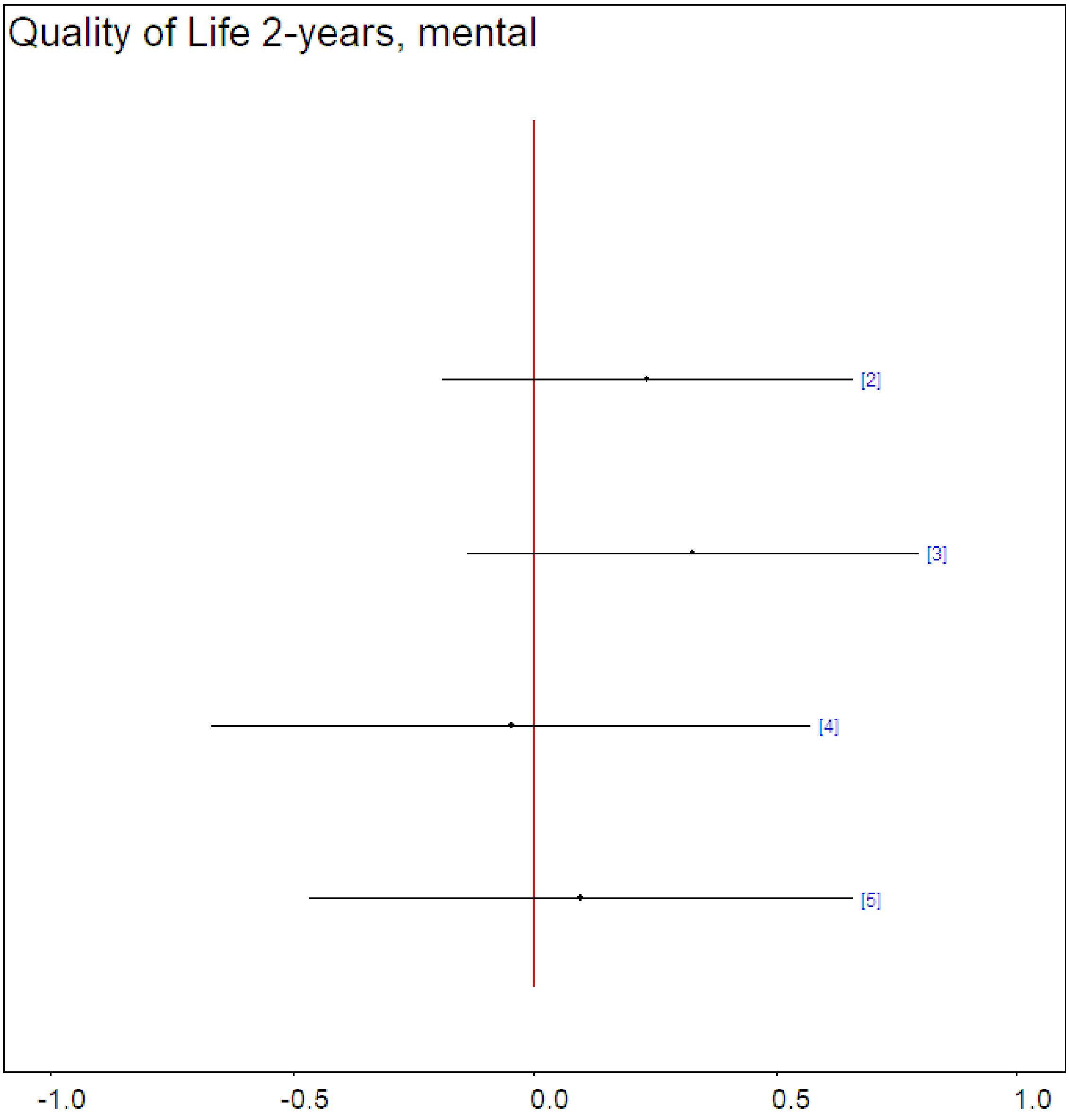

node	mean	sd	MC error	2.5%	median	97.5%
d[2]	0.2341	0.2172	7.8E-4	-0.1909	0.234	0.6601
d[3]	0.3271	0.2384	6.615E-4	-0.1392	0.327	0.7959
d[4]	-0.04757	0.3157	5.167E-4	-0.6671	-0.04759	0.5717
d[5]	0.09554	0.287	9.97E-4	-0.465	0.09492	0.6598

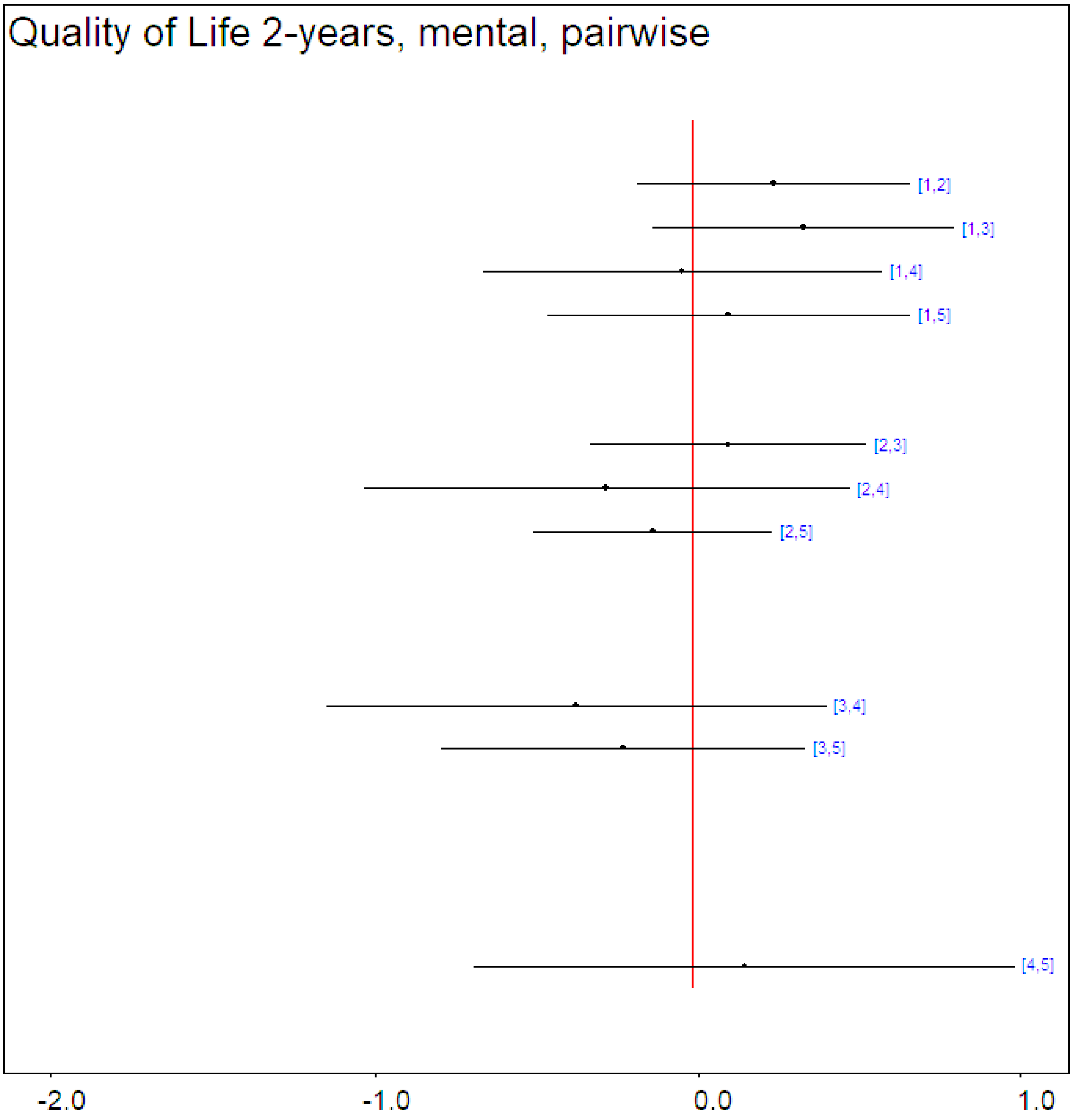

node	mean	sd	MC error	2.5%	median	97.5%
diff[1,2]	0.2341	0.2172	7.8E-4	-0.1909	0.234	0.6601
diff[1,3]	0.3271	0.2384	6.615E-4	-0.1392	0.327	0.7959
diff[1,4]	-0.04757	0.3157	5.167E-4	-0.6671	-0.04759	0.5717
diff[1,5]	0.09554	0.287	9.97E-4	-0.465	0.09492	0.6598
diff[2,3]	0.09297	0.2187	3.704E-4	-0.3364	0.09319	0.522
diff[2,4]	-0.2817	0.383	9.297E-4	-1.032	-0.2811	0.4699
diff[2,5]	-0.1386	0.1882	3.211E-4	-0.5079	-0.1383	0.2307
diff[3,4]	-0.3747	0.3954	8.355E-4	-1.15	-0.3753	0.3975
diff[3,5]	-0.2316	0.2881	5.834E-4	-0.7966	-0.2315	0.3339
diff[4,5]	0.1431	0.4269	0.00112	-0.6926	0.1429	0.9798

QoL 2-years bodily pain

3 studies

Legend:

1 LSG
2 LRYGB
3 BPD-DS
4 LAGB
5 Distal-GB

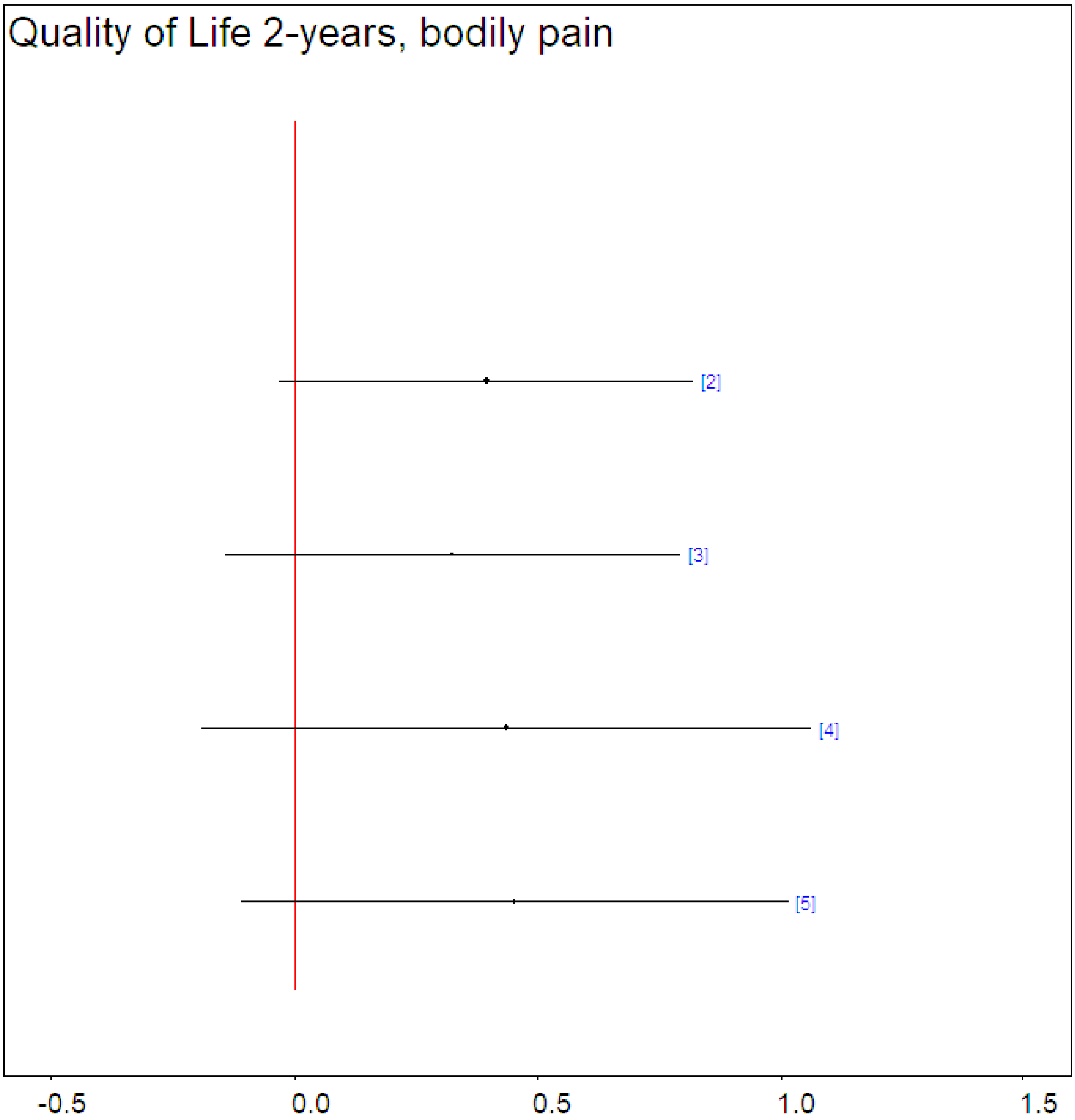

node	mean	sd	MC error	2.5%	median	97.5%
d[2]	0.3936	0.2176	7.841E-4	-0.03226	0.3935	0.8206
d[3]	0.3248	0.2394	6.673E-4	-0.1434	0.3246	0.7954
d[4]	0.4368	0.3197	5.233E-4	-0.1906	0.4368	1.064
d[5]	0.4518	0.2872	0.001001	-0.1094	0.4512	1.016

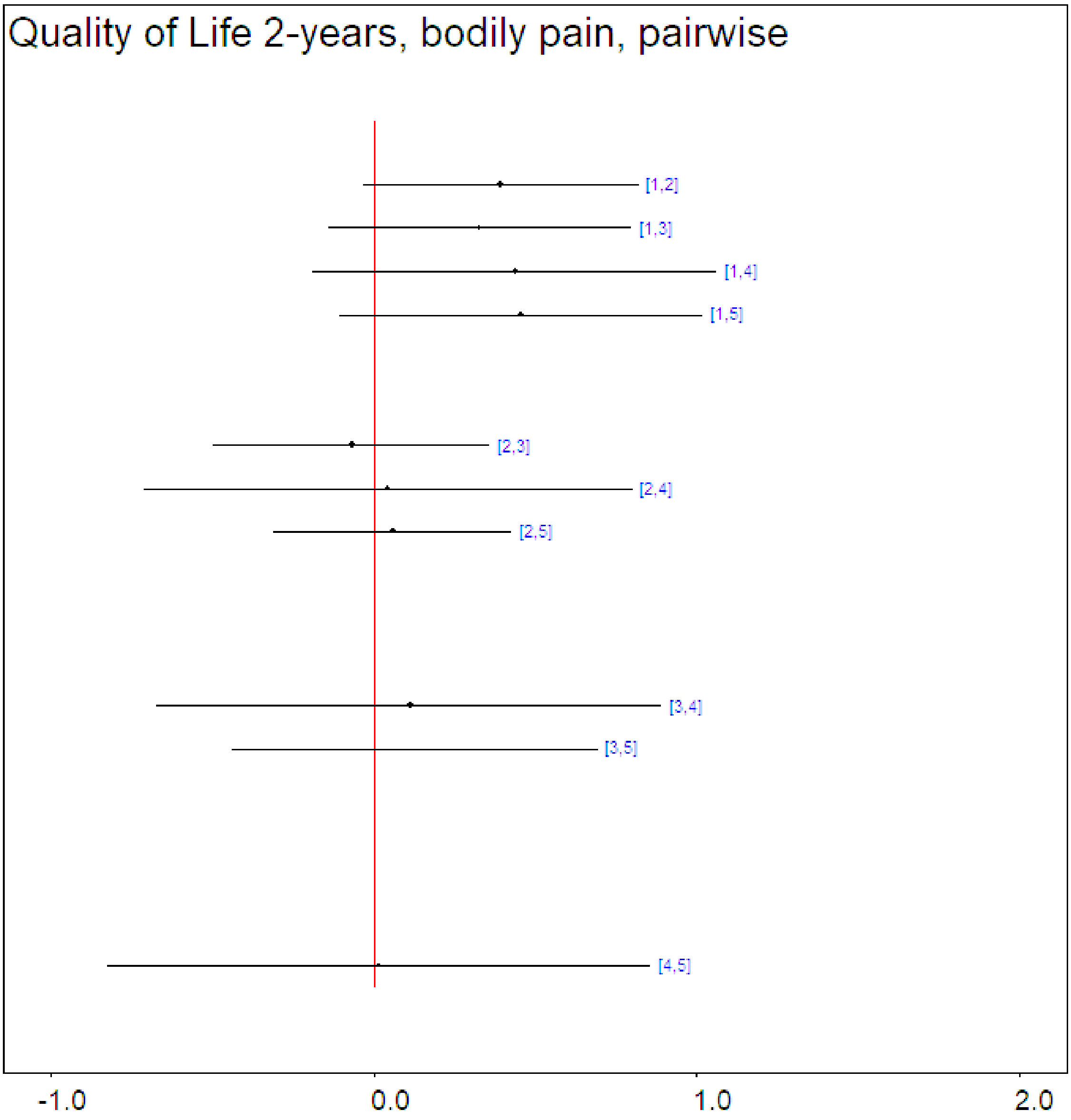

node	mean	sd	MC error	2.5%	median	97.5%
diff[1,2]	0.3936	0.2176	7.841E-4	-0.03226	0.3935	0.8206
diff[1,3]	0.3248	0.2394	6.673E-4	-0.1434	0.3246	0.7954
diff[1,4]	0.4368	0.3197	5.233E-4	-0.1906	0.4368	1.064
diff[1,5]	0.4518	0.2872	0.001001	-0.1094	0.4512	1.016
diff[2,3]	-0.06887	0.2187	3.696E-4	-0.4984	-0.06867	0.3601
diff[2,4]	0.04319	0.3866	9.366E-4	-0.7137	0.04378	0.8019
diff[2,5]	0.05816	0.188	3.207E-4	-0.3107	0.05842	0.4271
diff[3,4]	0.1121	0.3992	8.44E-4	-0.6711	0.1115	0.8915
diff[3,5]	0.127	0.288	5.821E-4	-0.438	0.1271	0.6924
diff[4,5]	0.01497	0.4301	0.001127	-0.8269	0.01458	0.8577

Total QoL 3-years

9 studies

Legend:
1 LI
2 LSG
3 LRYGB
4 BPD-DS
5 LAGB
6 Banded-GB

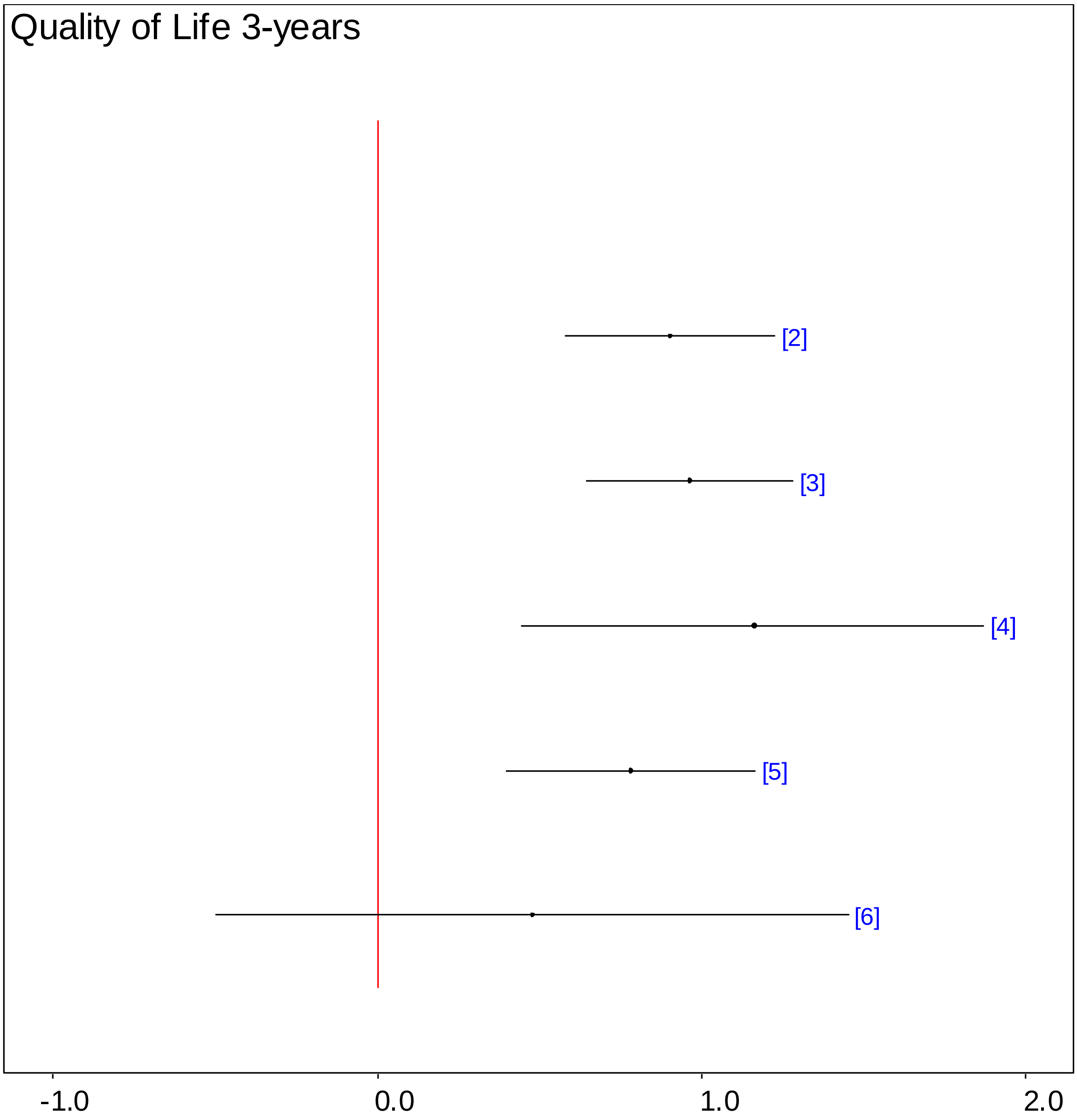

node	mean	sd	MC error	2.5%	median	97.5%
d[2]	0.9037	0.1658	0.001099	0.581	0.9032	1.23
d[3]	0.9646	0.1629	0.001065	0.6475	0.9641	1.285
d[4]	1.161	0.3646	0.001554	0.4455	1.162	1.874
d[5]	0.7831	0.1963	0.00117	0.3998	0.7828	1.168

d[6]	0.479	0.4985	0.00187	-0.4994	0.4784	1.455
totresdev	15.94	6.051	0.04308	6.006	15.3	28.59

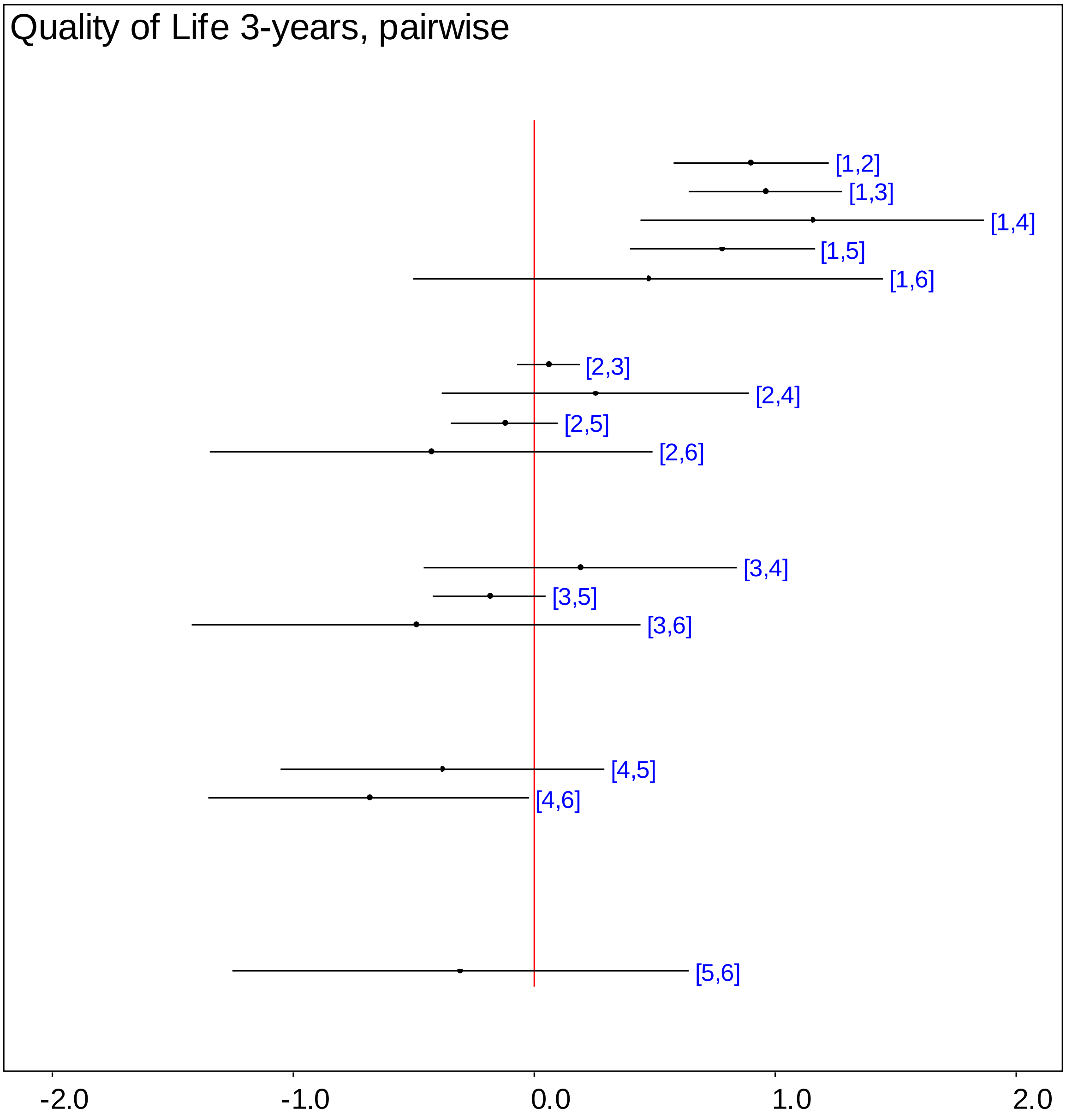

node	mean	sd	MC error	2.5%	median	97.5%
diff[1,2]	0.9037	0.1658	0.001099	0.581	0.9032	1.23
diff[1,3]	0.9646	0.1629	0.001065	0.6475	0.9641	1.285
diff[1,4]	1.161	0.3646	0.001554	0.4455	1.162	1.874
diff[1,5]	0.7831	0.1963	0.00117	0.3998	0.7828	1.168
diff[1,6]	0.479	0.4985	0.00187	-0.4994	0.4784	1.455
diff[2,3]	0.0609	0.06682	9.55E-5	-0.06991	0.06091	0.1922
diff[2,4]	0.2577	0.3251	9.344E-4	-0.3802	0.2578	0.8937
diff[2,5]	-0.1206	0.1127	1.852E-4	-0.3416	-0.1205	0.1003
diff[2,6]	-0.4247	0.4704	0.00136	-1.347	-0.4248	0.4979
diff[3,4]	0.1968	0.332	9.645E-4	-0.4544	0.1973	0.8462
diff[3,5]	-0.1815	0.1196	2.091E-4	-0.4164	-0.1815	0.05293
diff[3,6]	-0.4856	0.4749	0.001387	-1.417	-0.4861	0.446

diff[4,5]	-0.3783	0.3439	9.662E-4	-1.052	-0.3783	0.2946
diff[4,6]	-0.6823	0.3403	5.791E-4	-1.35	-0.6819	-0.01565
diff[5,6]	-0.304	0.4836	0.001383	-1.25	-0.3048	0.6438

Total QoL 5-years

7 studies

Legend:

1 LI
2 LSG
3 LRYGB
4 BPD-DS
5 OAGB

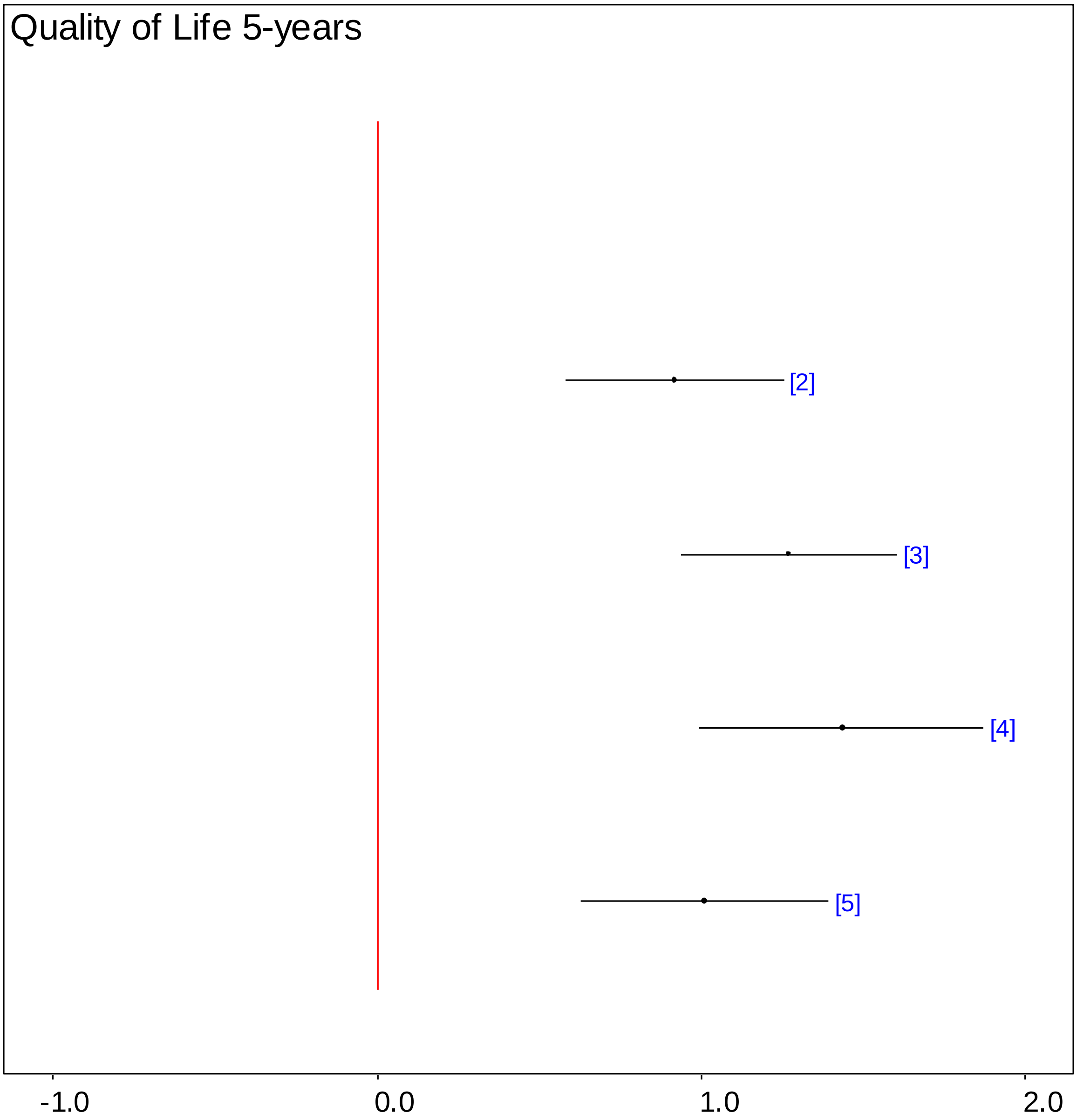

node	mean	sd	MC error	2.5%	median	97.5%
d[2]	0.9174	0.1713	0.001069	0.5841	0.9168	1.255
d[3]	1.269	0.1704	0.001085	0.9371	1.269	1.605
d[4]	1.434	0.2234	0.001188	0.9964	1.433	1.873

d[5]	1.009	0.1956	0.001154	0.6285	1.009	1.395
totresdev	9.02	4.253	0.007132	2.703	8.361	19.02

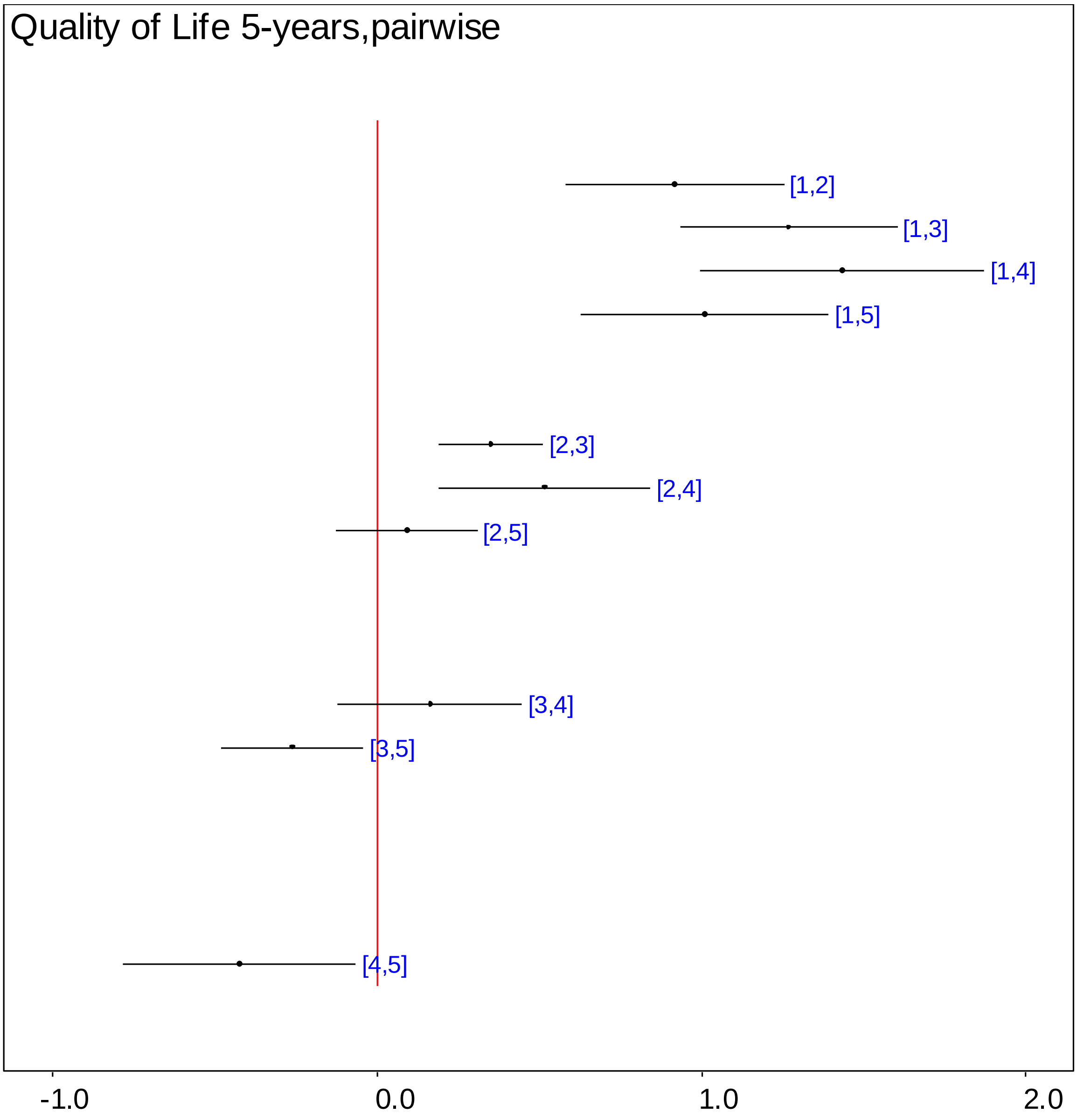

node	mean	sd	MC error	2.5%	median	97.5%
diff[1,2]	0.9174	0.1713	0.001069	0.5841	0.9168	1.255
diff[1,3]	1.269	0.1704	0.001085	0.9371	1.269	1.605
diff[1,4]	1.434	0.2234	0.001188	0.9964	1.433	1.873
diff[1,5]	1.009	0.1956	0.001154	0.6285	1.009	1.395
diff[2,3]	0.3518	0.08225	1.211E-4	0.1909	0.3519	0.5127
diff[2,4]	0.5164	0.1663	3.051E-4	0.1912	0.5166	0.8421
diff[2,5]	0.09205	0.1111	1.837E-4	-0.1261	0.09196	0.3104
diff[3,4]	0.1646	0.1448	2.446E-4	-0.1199	0.1648	0.4485
diff[3,5]	-0.2598	0.1116	1.879E-4	-0.4782	-0.2598	-0.04122
diff[4,5]	-0.4243	0.1827	3.236E-4	-0.7836	-0.4245	-0.06665
